# CsPbBr_3_ Perovskite‐Based Heterostructures in Photocatalysis: Mechanisms, Stability, and Multifunctional Performance

**DOI:** 10.1002/advs.202507747

**Published:** 2025-07-12

**Authors:** Kuanxin Lv, Zhenzhen Li, Xing Huang, Zetong Cheng, Zhongyan Wang, Hang Zhao

**Affiliations:** ^1^ College of Metallurgy and Energy North China University of Science and Technology Tangshan Hebei 063210 China

**Keywords:** CsPbBr_3_ perovskite, heterostructures, optimization strategies, photocatalysis, stability

## Abstract

CsPbBr_3_ perovskite stands out as a promising photocatalyst due to its strong visible‐light absorption and advantageous band positions, yet its practical application is constrained by rapid charge recombination and poor aqueous stability. This review systematically explores how heterostructure engineering, which encompasses Type‐II, Z‐scheme, and S‐scheme architectures, overcomes these limitations by optimizing interfacial charge dynamics and enhancing material durability. The underlying mechanisms of band alignment, charge transfer pathways, and redox potential retention in heterostructures, alongside strategies for activity modulation and stability enhancement are analyzed. By integrating insights from structural design to functional performance, the review illuminates how CsPbBr_3_‐based heterostructures address critical challenges in photocatalysis, offering a comprehensive framework for advancing sustainable solutions in energy conversion and environmental remediation.

## Graphical abstract

This review synthesizes breakthroughs in CsPbBr_3_ heterostructure photocatalysis: 1) **Mechanistic insights** on band alignment engineering (Type‐II/Z/S‐scheme) for suppressed charge recombination; 2) **Stabilization strategies** via interfacial encapsulation and defect passivation overcoming hydrolysis; 3) **Multifunctional design principles** enabling efficient CO_2_ conversion, H_2_ evolution, pollutant degradation, and organic synthesis.

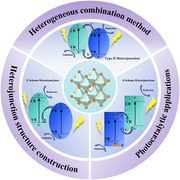

## 1 Introduction

Photocatalysis has emerged as a promising strategy due to its ability to harness solar energy for chemical transformations, pollution degradation, and clean fuel production under mild conditions.^[^ ^1^, ^2^ ^]^ At its core, photocatalysis relies on semiconductors that absorb photons to generate electron‐hole pairs, which then drive redox reactions at the material surface.^[^ ^3^, ^4^ ^]^ This process was first reported in 1972 when Fujishima and Honda demonstrated water splitting on TiO_2_ under UV light, marking the birth of photocatalytic research.^[^ ^5^ ^]^ Subsequent decades witnessed extensive exploration of metal oxides (e.g., TiO_2_, ZnO), chalcogenides (e.g., CdS), and carbon nitrides (g‐C_3_N_4_).^[^ ^6^, ^7^, ^8^, ^9^, ^10^ ^]^ Despite progress, traditional photocatalysts suffer from intrinsic limitations, including restricted light absorption, rapid charge recombination, and insufficient redox potentials under visible light.^[^ ^11^ ^]^ As summarized in **Table** **1**, representative non‐perovskite materials exhibit moderate performance in organic pollutant degradation and hydrogen production, yet their practical applicability is constrained by suboptimal carrier utilization and stability issues.^[^ ^12^, ^13^, ^14^, ^15^, ^16^, ^17^, ^18^, ^19^ ^]^ These bottlenecks necessitate the exploration of advanced materials with tailored optoelectronic properties and enhanced stability.

**Table 1:** Comparison and summary of the photocatalytic performance of non‐perovskite materials.

No.	Sample	Application	Synthesis method	Photocatalytic performance	Refs.
1	Bi‐TiO_2_	Degradation of phenol wastewater	Hydrothermal synthesis	Degradation efficiency of phenol is 86.4% (5 mg L^−1^ phenol, 180 min)	[12]
2	MoS_2_/SrTiO_3_	Degradation of RhB wastewater	Two‐step hydrothermal synthesis	Degradation efficiency of RhB is 92.15% (5 mg L^−1^ RhB, 50 min)	[13]
3	Cu‐BiVO_4/_MMTNs	Degradation of phenol wastewater	Hydrothermal method	Degradation efficiency of phenol is 99.32% (20 mg L^−1^ phenol, 140 min)	[14]
4	CdS(h/c)	H_2_ production	Hydrothermal synthesis	H_2_ evolution rate is 4.9 mmol g^−1^ h^−1^ (5% concentration ammonia solution)	[15]
5	rGO‐ZnO‐CdSe	Degradation of BR46 dye wastewater	Hydrothermal synthesis	Degradation efficiency of phenol is 94.59% (14.05 mg L^−1^ BR46 dye, 240 min)	[16]
6	g‐C_3_N_4_/MoS_2_	Degradation of RhB wastewater	Solution thermal calcination	Degradation efficiency of RhB is 99.4% (10 mg L^−1^ RhB, 90 min)	[17]
7	rGO‐TiO_2_‐g‐C_3_N_4_	Degradation of MB wastewater	Green synthesis	Degradation efficiency of MB is 98.5% (20 mg L^−1^ MB, 120 min)	[18]
8	CdS/MoC	H_2_ production	Facile physical mixing	H_2_ evolution rate is 224.5 µmol g^−1^ h^−1^ (10 vol% lactic acid solution)	[19]

Abbreviations: MB, methylene blue; MMTNs, montmorillonite nanosheets; RhB, rhodamine B.

Metal halide perovskites have revolutionized optoelectronics, achieving a meteoric rise in photovoltaic efficiency from 3.8% to 27% in recent years.^[^ ^20^, ^21^ ^]^ Beyond solar cells, their outstanding properties‐tunable bandgaps, high absorption coefficients, and ultralong carrier diffusion lengths‐position them as promising candidates for photocatalytic applications.^[^ ^22^, ^23^ ^]^ The organic–inorganic hybrid perovskites, such as CH_3_NH_3_PbI_3_ and CH_3_NH_3_PbBr_3_ have demonstrated exceptional efficiency in photovoltaic applications but suffer from inherent stability vulnerabilities that severely limit their photocatalytic potential.^[^ ^24^, ^25^ ^]^ Organic cations (MA^+^ or FA^+^) decompose under thermal/light stress, releasing volatile amines and halides, which accelerates structural collapse.^[^ ^26^, ^27^ ^]^ Critically, these materials undergo rapid hydrolysis in humid environments, degrading the perovskite phase into inactive PbI_2_/PbBr_2_.^[^ ^28^ ^]^ Such vulnerabilities not only compromise the long‐term durability of hybrid perovskites but also restrict their applicability in aqueous photocatalytic reactions where moisture and light exposure are inevitable.

To address these stability challenges, all‐inorganic perovskites (CsPbX_3_, X = I, Br, Cl) have emerged prominently in the field of photocatalysis. Cubic CsPbI_3_ exhibits a photoactive black phase (α‐phase, 1.73 eV) theoretically capable of efficient light absorption, but it readily transforms into a non‐perovskite δ‐phase (2.8 eV) under low‐temperature and high‐humidity conditions, leading to significant degradation in photocatalytic activity.^[^ ^29^, ^30^ ^]^ In contrast, CsPbBr_3_ stands out owing to its unique structural characteristics. The orthorhombic phase (Pbnm) of CsPbBr_3_ exhibits excellent thermal stability with no significant decomposition below 690 K and superior moisture resistance.^[^ ^31^, ^32^, ^33^, ^34^ ^]^ This remarkable stability stems from the strong ionic bonding between Cs+ and Br‐ ions and the lattice stabilization mediated by an optimal Goldschmidt tolerance factor (*t* = 0.82).^[^ ^35^ ^]^ More significantly, CsPbBr_3_ exhibits a theoretically calculated bulk bandgap of 2.36 eV in its orthorhombic phase, with the conduction band minimum (CBM) at ≈−1.3 V versus NHE and the valence band minimum (VBM) at +1.0 V versus NHE.^[^ ^36^, ^37^, ^38^ ^]^ The highly negative conduction band potential of CsPbBr_3_ at −1.3 V versus NHE provides a robust thermodynamic driving force for fuel generation, significantly exceeding the reduction potentials required for CO_2_‐to‐CO conversion (−0.52 V vs NHE)^[^ ^39^ ^]^ and hydrogen evolution (0 V vs NHE).^[^ ^40^ ^]^ Meanwhile, the VBM at +1.0 V versus NHE, while thermodynamically insufficient for direct water oxidation to ·OH (E > +1.23 V vs NHE), enables hole‐mediated oxidation via surface chemistry or heterostructure design.^[^ ^41^ ^]^ This can be achieved by circumventing the O─H bond cleavage barrier through defect engineering to activate localized oxidative pathways and band alignment engineering to elevate the effective valence band potential above +1.23 V. Despite these merits, pristine CsPbBr_3_ faces critical limitations including ultrafast charge recombination at surface defects and ion migration‐induced instability under operational conditions.^[^ ^42^, ^43^, ^44^ ^]^ Moreover, targeted applications often require precise band alignment engineering to optimize the energetic landscape for specific redox processes. To provide a comprehensive overview of these stability and performance differences across halide perovskite materials, **Table** **2** presents a detailed comparative analysis of key properties, advantages, challenges, and application scope for representative organic–inorganic hybrid and all‐inorganic perovskites.

**Table 2:** Comparative properties and applications of halide perovskite materials.

Material	Bandgap [eV]	Stability	Major limitations	Primary Applications
MAPbBr_3_^[^ ^24^, ^45^ ^]^	≈2.3	Decomposes >100–130 °C; Highly sensitive to moisture	Hygroscopic; Volatile MA^+^	Photovoltaics
FAPbBr_3_^[^ ^45^, ^46^ ^]^	≈2.2	Highly sensitive to moisture	Moisture degradation FA⁺ decomposition	Photovoltaics
CsPbI_3_^[^ ^29^, ^47^ ^]^	1.7 (α‐phase); >2.8 (δ‐phase)	Thermally stable; Sensitive to moisture	Phase instability (α→δ transition)	Photovoltaics (stability‐limited)
CsPbBr_3_^[^ ^32^, ^33^ ^]^	≈2.36	Superior stability	Charge recombination	Photocatalysis: CO_2_ reduction; Water splitting

To overcome these limitations, constructing CsPbBr_3_‐based heterostructures has emerged as a transformative strategy, synergizing the advantages of CsPbBr_3_ with complementary materials to address interfacial charge dynamics and stability simultaneously.^[^ ^48^, ^49^, ^50^, ^51^ ^]^ Heterostructure engineering enables precise control over charge transfer pathways and defect passivation, unlocking unprecedented photocatalytic performance. For instance, Type‐II heterostructures like CsPbBr_3_/g‐C_3_N_4_ spatially separate electrons and holes, extending carrier lifetimes from 32 µs (pristine CsPbBr_3_) to 60 µs.^[^ ^52^ ^]^ The Z‐scheme architecture, such as Ag/CsPbBr_3_/Bi_2_WO_6_, maintained a strong redox potential and suppressed recombination, achieving a 93.9% pollutant degradation rate within 120 min, which is 4.41 times higher than that of individual components.^[^ ^53^ ^]^ Additionally, S‐scheme heterostructures like BiOBr/Bi‐doped CsPbBr_3_ demonstrate exceptional CO_2_ reduction performance through built‐in electric field‐driven charge separation, achieving 151.56 µmol g^−1^ h^−1^ with 93.6% selectivity in CO_2_ reduction, representing a 3.62‐fold enhancement over pure BiOBr, where Bi doping optimizes band alignment and facilitates efficient interfacial charge transfer.^[^ ^54^ ^]^ These promising results highlight the tremendous potential of rationally designed CsPbBr_3_‐based heterostructures in overcoming the inherent limitations of pristine materials, while opening new avenues for enhanced performance across various photocatalytic applications.

In this review, we systematically examined the synthesis methodologies, heterostructure optimization strategies, and photocatalytic mechanisms of CsPbBr_3_‐based materials. Moreover, we focus particularly on the major challenges facing CsPbBr_3_ in various applications, including water splitting, pollutant degradation, CO_2_ reduction, and photocatalytic organic synthesis.

## 2 Crystal Nature of Perovskite

### 2.1 Structural Framework and Phase Transition Behavior

CsPbBr_3_ exhibits a prototypical three‐dimensional perovskite structure, consisting of corner‐sharing [PbBr_6_]^4‐^ octahedra forming a 3D network, with Cs^+^ ions occupying the interstitial sites. At room temperature, CsPbBr_3_ crystallizes in the orthorhombic system (space group Pbnm) with lattice parameters *a* = 8.37 Å, *b* = 8.43 Å, and *c* = 12.01 Å.^[^ ^55^, ^56^ ^]^ Thermodynamically, the stability of CsPbBr_3_ is supported by its formation enthalpy from binary halides (ΔH = −11.64 ± 1.17 kJ mol^−1^).^[^ ^57^ ^]^ The negative enthalpy indicates an exothermic formation process, confirming intrinsic thermodynamic stability under ambient conditions.^[^ ^58^ ^]^ Moreover, the structural stability and phase behavior of perovskites are governed by the Goldschmidt tolerance factor (t),^[^ ^59^ ^]^ defined as follows:

(1) $$t=\left(r_{A}+r_{X}\right)/\left[\sqrt{2}\left(r_{B}+r_{X}\right)\right]$$

where *r*_A_, *r*_B_, and *r*_X_ are the ionic radii of the A‐site cation (Cs^+^), B‐site cation (Pb^2+^), and anion (Br^−^), respectively. This parameter quantifies the geometric compatibility of ions in the perovskite lattice, predicting phase transitions based on octahedral tilting.^[^ ^59^ ^]^ For CsPbBr_3_ (*t* = 0.82), the value falls within the orthorhombic stability range at room temperature, as illustrated in **Table** **3**, consistent with its observed Pbnm symmetry.^[^ ^60^ ^]^ This suboptimal *t* value prevents cubic phase stabilization due to significant PbBr6 octahedral distortion, which is only alleviated at elevated temperatures where thermal energy suppresses tilting and triggers phase transitions to higher‐symmetry structure.

**Table 3:** Phase Transitions and Structural Characteristics of CsPbBr_3_ Perovskite.

t Range	Stable Phase	Space Group	Structural Implications	Temperature
T≈1.0	Cubic	Pm3̄m	Ideal perovskite; high symmetry	>403 K
0.9<t<1.0	Tetragonal	P4/mbm	Moderate octahedral tilting	361–403 K
0.7<t<0.9	Orthorhombic	Pbnm	Severe octahedral tilting	<361 K

**Figure 1:**
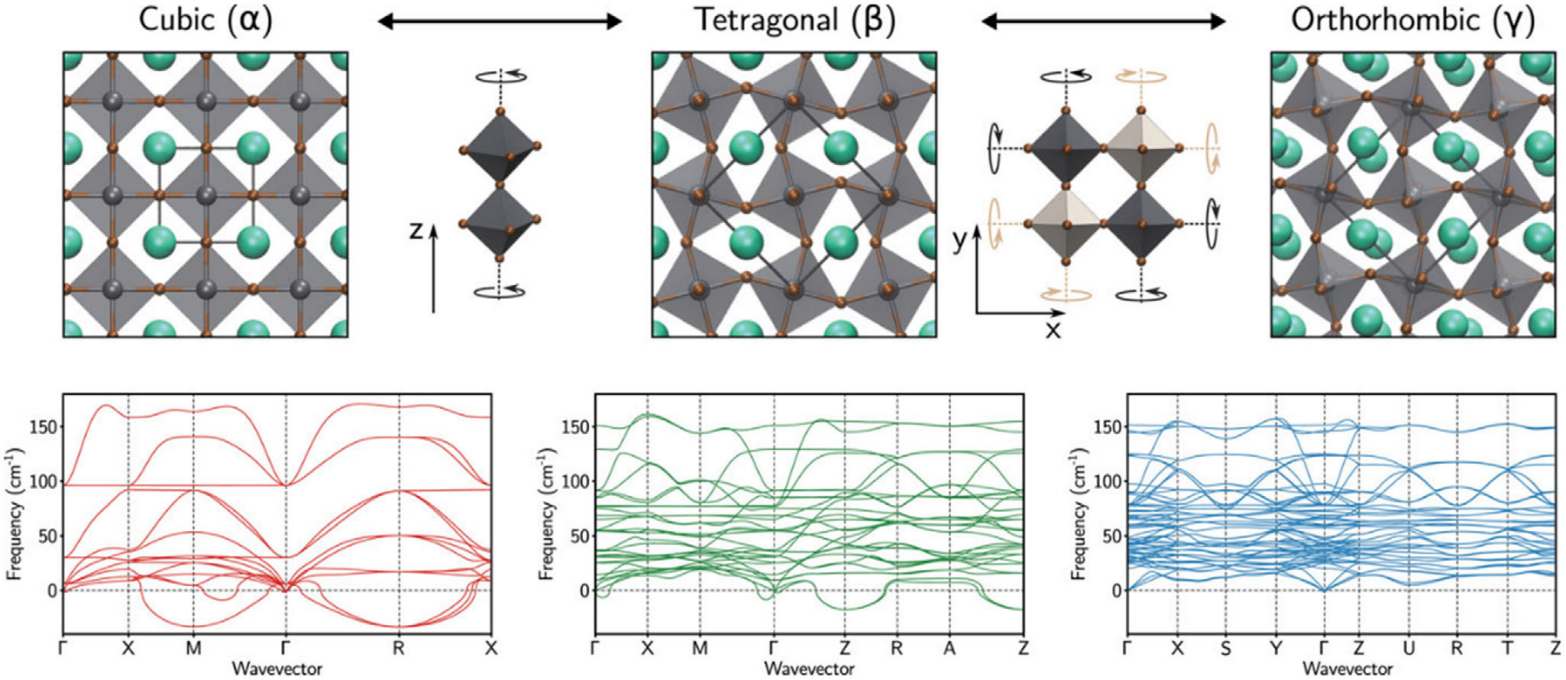
Structural phases of CsPbBr_3_ perovskite and their corresponding phonon properties. Reproduced with permission.^[^ ^55^ ^]^ Copyright 2023, AIP.

Recent studies reveal that the dynamic tilting of [PbBr_6_]^4−^ octahedra plays a critical role in stabilizing the orthorhombic phase under ambient conditions, with Br^−^ ions exhibiting anisotropic thermal vibrations along the *c*‐axis (**Figure** **1**).^[^ ^55^, ^63^, ^64^ ^]^ Upon increasing temperature, CsPbBr_3_ undergoes two sequential structural phase transitions that significantly alter its crystallographic and electronic properties. The initial transition occurs within the temperature range of 361–403 K (88–130 °C), wherein the material transforms from the orthorhombic phase (space group Pbnm) to a tetragonal phase (space group P4/mbm). High‐resolution synchrotron‐based grazing‐incidence wide‐angle X‐ray scattering measurements, corroborated by molecular dynamics simulations, reveal that this transition is accompanied by an ≈2.5% contraction in unit‐cell volume.^[^ ^64^ ^]^ Concomitantly, the tilting angle of [PbBr_6_]^4‐^ octahedra decreases from ≈8.5° to 4.2°, while preserving the in‐phase rotational symmetry along the crystallographic *c*‐axis. This coherent lattice distortion manifests through coordinated atomic displacements that maintain structural coherence within the tetragonal phase through thermally activated processes.

**Figure 2:**
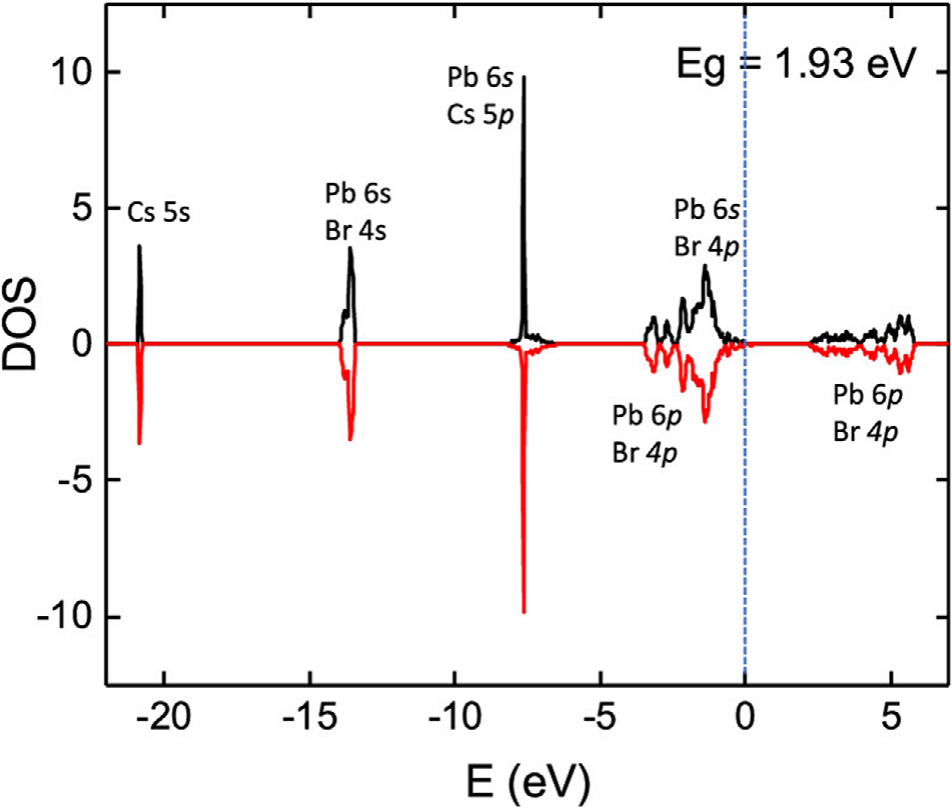
Total DOS for CsPbBr_3_ depicting a band gap energy of 1.93 eV. The black curves represent spin‐up states, while the red curves represent spin‐down states. A vertical dashed line indicates the Fermi level. Reproduced under the terms of the Creative Commons Attribution 4.0 International License (CC BY 4.0).^[^ ^64^ ^]^ Copyright 2024, Copyright Khan et al.

As the temperature exceeds 403 K (≈130 °C), a second phase transition initiates, transforming the structure to the cubic phase (space group Pm3̄m). This higher‐symmetry phase is characterized by the complete suppression of octahedral tilting and the establishment of maximal crystallographic symmetry. Ab initio density functional theory calculations demonstrate that above 410 K, the collapse of dynamic octahedral tilting mechanisms leads to a significant narrowing of the electronic bandgap from ≈2.3 eV in the orthorhombic phase to ≈1.9 eV in the cubic phase, corresponding to a red shift of ≈0.4 eV.^[^ ^64^ ^]^ This thermally‐induced bandgap modulation is directly correlated with enhanced lattice symmetry and modified orbital overlap between the Pb 6s and Br 4p states, as the DOS results shown in **Figure** **2**.^[^ ^64^ ^]^

To sum up, temperature‐driven structural transitions in CsPbBr_3_, governed by thermal expansion and octahedral rotation dynamics, determine its carrier mobility, bandgap tunability, and structural stability, providing crucial guidance for designing efficient photocatalysts with optimized charge separation and operational durability.

### 2.2 Defect Tolerance Mechanism and Electronic Structure

The defect tolerance of CsPbBr_3_, originating from its unique electronic structure and ion‐electron coupling, ensures robust optoelectronic performance under high defect concentrations. This intrinsic property is critical for photocatalytic applications, as it facilitates efficient charge separation and preserves catalytic activity by suppressing deep‐level trap‐induced energy losses. As reported in seminal studies,^[^ ^65^, ^66^ ^]^ halide vacancies (*V*_Br_) and lead vacancies (*V*_Pb_) are the dominant intrinsic defects. Under Br‐rich conditions, *V*_Br_ exhibits exceptionally low formation energies (0.25–0.4 eV), while *V*_Pb_ forms readily due to the high mobility of Pb^2+^ ions under thermal or photoexcitation.

Previous studies reveal that most intrinsic defects, such as *V*_Br_ and *V*_Pb_, generate shallow energy levels (<0.5 eV) near the CBM or VBM, avoiding the formation of deep non‐radiative recombination centers.^[^ ^65^, ^66^, ^67^ ^]^ This shallow‐level characteristic is further enhanced by spin‐orbit coupling (SOC), which promotes spatial delocalization of defect‐related electronic states. First‐principles calculations show that SOC broadens defect wavefunctions, reducing the formation of localized traps and minimizing carrier confinement, a key factor in suppressing non‐radiative recombination.^[^ ^68^ ^]^ Additionally, strong hybridization between Pb 6p and Br 4p orbitals creates dispersive, shallow energy bands where defect states extend across neighboring lattice sites.^[^ ^69^ ^]^ This orbital delocalization reduces the carrier trapping probability by blurring the spatial localization of defect states, which is a key factor in the tolerance of CsPbBr_3_ to structural disorder.

Despite the inherent bulk defect tolerance of CsPbBr_3_, surface and interfacial defects remain key non‐radiative recombination pathways, necessitating targeted passivation strategies. Ligand engineering has emerged as an effective approach to regulate surface defect states due to its capability to passivate undercoordinated surface ions, particularly dangling Pb^2+^ and Br^‐^ bonds through coordinate bond formation, which eliminates deep trap states and reduces non‐radiative recombination centers, thereby enhancing photoluminescence quantum yield and extending carrier lifetimes. Additionally, ligand engineering improves surface stability against environmental degradation by forming protective molecular barriers that prevent moisture and oxygen infiltration, thus maintaining structural integrity under operational conditions.^[^ ^70^, ^71^ ^]^ Rogach et al. demonstrated that surface Br^‐^ ions forming hydrogen bonds with organic ligands create Br‐rich passivation layers capable of suppressing Br vacancy formation.^[^ ^70^ ^]^ In CsPbBr_3_ nanocrystals (NCs) terminated with bidentate Lewis base ligands such as 1,4‐bis(diphenylphosphino)butane (DBPP), electron donation from the ligand to the NCs modulates electron density and energy levels, thereby enhancing photocatalytic CO_2_ reduction performance. Interfacial defect engineering via heterostructure construction further addresses non‐radiative recombination. For instance, Gao et al. demonstrated that confinement of ultrafine CsPbBr_3_ nanocrystals within extra‐large‐pore zeolite (ZEO‐1) creates a nanoscale platform that simultaneously achieves abundant exposed active sites, enhanced stability in acidic media, and band gap narrowing, resulting in a remarkable 157‐fold enhancement in photocatalytic hydrogen evolution rate (1734 µmol h^−1^ g^−1^) compared to bulk CsPbBr_3_.^[^ ^71^ ^]^

Therefore, the synergy between the inherent defect tolerance of CsPbBr_3_ perovskites and rational heterostructure engineering provides critical theoretical guidance for designing perovskite photocatalysts. By modulating bulk defect states and interfacial electronic structures, this approach enables the suppression of non‐radiative recombination while maintaining high catalytic activity, thereby paving the way for the development of efficient and stable photocatalytic systems.

### 2.3 Degradation Mechanisms of CsPbBr_3_ Perovskite

Although exhibiting superior thermo‐photostability over hybrid perovskites, CsPbBr_3_ suffers from critical degradation in polar environments such as aqueous media and organic solvents. This degradation dominates its practical photocatalytic performance, thus necessitating mechanistic studies on solvent‐induced degradation pathways.^[^ ^72^, ^73^ ^]^ The degradation in polar solvents proceeds through fundamentally different mechanisms compared to thermal or photo‐induced degradation, primarily involving solvation‐driven ion extraction and coordination‐induced lattice destabilization.^[^ ^74^ ^]^ Understanding these mechanisms is crucial for designing stable CsPbBr_3_‐based heterostructured photocatalysts that can operate effectively in realistic reaction environments.

#### 2.3.1 Aqueous Degradation Pathways

Protic solvents, including water and alcoholic solvents, induce systematic degradation of CsPbBr_3_ perovskites through distinct yet interrelated mechanisms governed by proton transfer and hydrogen‐bonding interactions. In aqueous environments, degradation follows a two‐stage process: initial adsorption of water molecules onto undercoordinated Pb^2+^ sites at the surface, followed by lattice intercalation that triggers phase transformation.^[^ ^75^ ^]^ Density functional theory calculations show that water molecules coordinate with Pb^2+^ via the hard–soft acid‐base principle, forming unstable hydrated complexes that decompose into CsPb_2_Br_5_ intermediates and CsBr byproducts.^[^ ^76^ ^]^ This intermediate phase further hydrolyzes to produce PbBr_2_, with its tetragonal structure exhibiting a reduced bandgap that accelerates decomposition.^[^ ^77^, ^78^ ^]^ Moreover, real‐time structural evolution during aqueous degradation has been captured through advanced in situ characterization techniques, with Ma et al. providing unprecedented atomic‐scale visualization of water‐induced degradation pathways through in situ liquid‐phase transmission electron microscopy, revealing that degradation initiates preferentially at surface defect sites, particularly halide vacancies, and progresses through progressive dissolution of [PbBr_6_]^4−^ octahedral units.^[^ ^79^ ^]^

Alcoholic solvents degrade CsPbBr_3_ via dual hydrogen‐bond donor/acceptor interactions, with hydroxyl groups disrupting Pb‐Br bonds through competitive coordination.^[^ ^80^, ^81^ ^]^ Notably, polar solvents bearing the same functional group (e.g., hydroxyl) exhibit progressively more severe degradation of CsPbBr_3_ as solvent polarity increases, a trend consistent with the enhanced disruption of Pb–Br frameworks by stronger dipole‐dipole interactions.^[^ ^82^ ^]^ This mechanistic behavior mirrors the degradation kinetics in water, where proton‐assisted dissolution accelerates ion mobility and lattice collapse.

#### 2.3.2 Polar Organic Solvent Degradation

CsPbBr_3_ degradation in polar aprotic solvents proceeds through coordination complex formation rather than simple ionic dissolution. Research has established that high donor number (DN) solvents coordinate more strongly with the Pb^2+^ center, which inhibits bromide coordination and disrupts perovskite crystallization.^[^ ^83^, ^84^, ^85^ ^]^ This coordination‐competition mechanism explains why highly coordinating solvents like DMSO (DN = 29.8) and DMF (DN = 26.6) cause rapid structural collapse through direct metal‐ligand interactions.^[^ ^86^, ^87^ ^]^ During degradation, polar aprotic solvents compete with bromide ligands for coordination sites on the lead center, resulting in the formation of solvated lead complexes while simultaneously releasing cesium and bromide ions into solution. For instance, DMSO molecules coordinate with the lead center to form [Pb(DMSO)_n_]^2+^ complexes, which further lead to complete disintegration of the perovskite crystal structure. The degradation rate correlates directly with solvent donor number and dielectric constant, with complete dissolution occurring within minutes in highly polar solvents.

Thus, understanding the degradation mechanisms of CsPbBr_3_ in polar solvents is crucial for guiding rational solvent selection and stability optimization in photocatalytic systems. Based on its degradation characteristics in polar solvents, solvent selection for CsPbBr_3_ photocatalysis must comprehensively consider factors such as polarity, proton activity, hydrogen‐bonding capacity, surface interactions, and reaction compatibility. Non‐polar and low‐polar solvents such as ethyl acetate and toluene are preferred, as they can minimize lattice disruption while maintaining catalytic activity.^[^ ^88^, ^89^, ^90^ ^]^ Combined with surface passivation^[^ ^91^, ^92^ ^]^ or solvent engineering strategies,^[^ ^93^, ^94^ ^]^ these approaches enable simultaneous enhancement of structural stability and photocatalytic efficiency, paving the way for practical applications of CsPbBr_3_‐based heterostructures in real‐world reaction environments.

## 3 Synthesis Methods of CsPbBr_3_

The synthesis methods of CsPbBr_3_ play a crucial role in defining its crystal structure, optical properties, and ultimately its performance in photocatalytic applications. As researchers aim to enhance the photocatalytic efficiency and stability of CsPbBr_3_, developing reliable and effective synthesis strategies becomes essential for tailoring its properties to meet specific requirements. These synthesis methods, such as hot injection, solvothermal processing, ion exchange, room‐temperature ligand‐assisted precipitation, template self‐assembly, ultrasound/microwave‐assisted techniques, and emulsion‐based approaches, exhibit unique advantages (e.g., high yield, precise morphology control, and green chemistry compatibility) but also face inherent limitations. Key trade‐offs involve compromised stability, procedural complexity, and scalability constraints, with a comprehensive comparison provided in **Table** **4**.

**Table 4:** Summary of the advantages and disadvantages of various synthesis methods.

No.	Synthesis method	Advantages	Disadvantages	Refs.
1	Hot injection method	Rapid response and high yield, suitable for doping and composite materials.	High requirements for injection speed, insufficient product stability	[95]
2	Solvothermal method	High crystallinity, fewer defects, controllable morphology, and excellent optoelectronic properties.	Solvent has strong toxicity, high dependence on temperature, and pressure	[96]
3	Ion Exchange method	Fast, efficient, low toxicity, and environmentally friendly, suitable for large‐scale preparation.	Inconsistent in uniformity of nanocrystals and difficulty in controlling size and morphology	[97]
4	Room temperature ligand‐assisted precipitation method	High yield and good dispersibility, easy to operate, fast, and efficient.	Strong dependence on ligand and solvent system, product purity	[98]
5	Template self‐assembly method	Precise control of morphology and size, green and mild conditions.	Complexity in template preparation/removal, high cost, poor repeatability	[99]
6	Ultrasound‐assisted synthesis method	Fast, efficient, green, and environmentally friendly.	High requirements for equipment and sensitivity to reaction conditions.	[100]
7	Microwave‐assisted synthesis method	Fast and efficient, green and environmentally friendly, heating evenly.	Equipment costs are high and difficult to scale up production.	[101]
8	Emulsion method	Green, environmentally friendly, low‐cost, and stable	Dependence of surface active agents on limited product purity and dispersibility.	[102]

### 3.1 Ion Exchange Method

**Figure 3:**
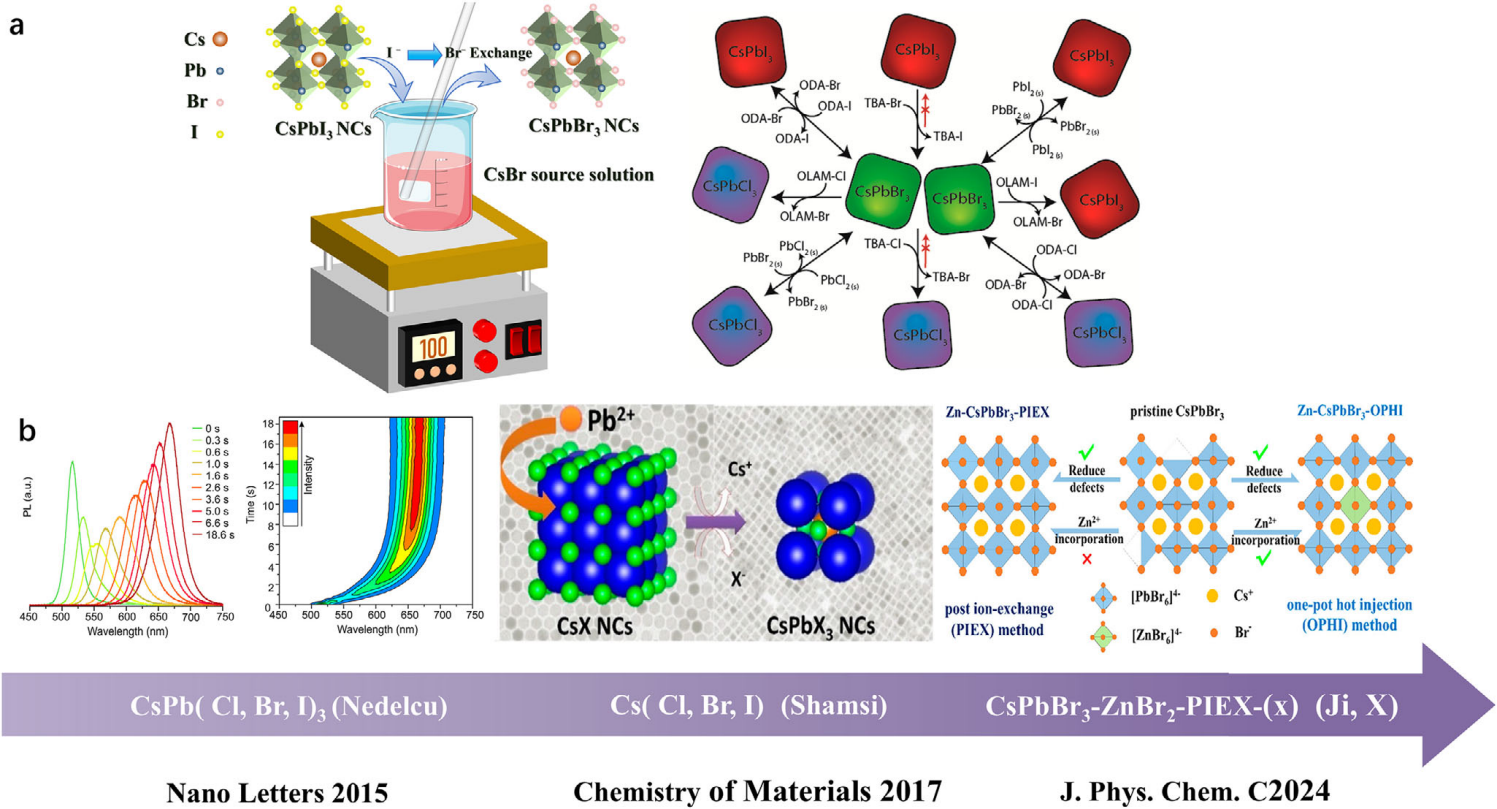
a) Schematic diagram of CsPbBr_3_ NCs synthesized by ion exchange method and overview of the Different Routes and Precursors for the Anion Exchange Reactions on CsPbX_3_ (X = Cl, Br, I) NCs. Reproduced under the terms of the Creative Commons Attribution 4.0 International License (CC BY 4.0).^[^ ^108^ ^]^ Copyright 2015, Copyright Akkerman et al. b) Key milestones in the development of CsPbBr_3_ perovskite synthesis via ion exchange method.

As shown in **Figure** **3**a, the ion exchange method is a simple and scalable synthetic technique, typically involving the pre‐synthesis of CsPbCl_3_ or CsPbI_3_ nanocrystals via hot injection.^[^ ^96^, ^103^ ^]^ These nanocrystals are then immersed in a bromide solution at moderate temperatures (60–100 °C), enabling efficient substitution of chloride or iodide ions with bromide ions while preserving the perovskite lattice integrity. Key parameters such as reaction time, halogen concentration, and temperature critically influence the final morphology and size of CsPbBr_3_ nanocrystals. This approach yields well‐dispersed nanocrystals and allows precise tuning of their bandgap (*E*_g_), valence band (VB), and conduction band (CB) potentials through the anion exchange process.^[^ ^104^ ^]^

As shown in Figure 3b, the key milestone in the synthesis of CsPbBr_3_ perovskite by the ion exchange method is demonstrated. In 2015, Nedelcu et al. pioneered the anion exchange synthesis of all‐inorganic perovskite CsPbX_3_ (X = Cl, Br, I), demonstrating control over luminescent wavelengths across the entire visible spectrum (410–700 nm) by adjusting halide ion ratios in solution at 40 °C.^[^ ^105^ ^]^ This method maintained high photoluminescence quantum yields (PLQYs) of 20%–80%, establishing the feasibility of optical property tuning via solution‐phase anion exchange and laying the foundation for subsequent research. Subsequently, Shamsi et al. developed a cation exchange strategy (Cs^+^→Pb^2+^), producing CsPbX_3_ nanocrystals with sizes ranging from 7–23 nm under ambient conditions.^[^ ^106^ ^]^ While this approach offered a novel room‐temperature synthesis pathway, abundant surface defects resulted in moderate PLQYs (32%–50%).

Xing et al. addressed this limitation by doping Zn^2+^ into cation‐exchanged CsPbBr_3_ nanocrystals, achieving a significant PLQY enhancement (up to 86%) through defect passivation.^[^ ^107^ ^]^ Specifically, the defect passivation mechanism involves Zn^2+^ ions effectively filling Pb vacancies and neutralizing undercoordinated surface ions, while forming PbBr^2‐^ terminated surface layers that suppress halide vacancies and reduce Urbach energy from 28 meV to 21–24 meV. These structural modifications modulate charge carrier dynamics through weaker non‐adiabatic coupling between conduction bands, resulting in extended carrier lifetimes and reduced non‐radiative recombination pathways that collectively eliminate trap states and improve photoluminescence efficiency. These works collectively demonstrate the versatility of ion exchange methods in tailoring CsPbBr_3_ nanostructures for applications requiring precise control over optical and electronic properties.

### 3.2 Solvothermal Method

**Figure 4:**
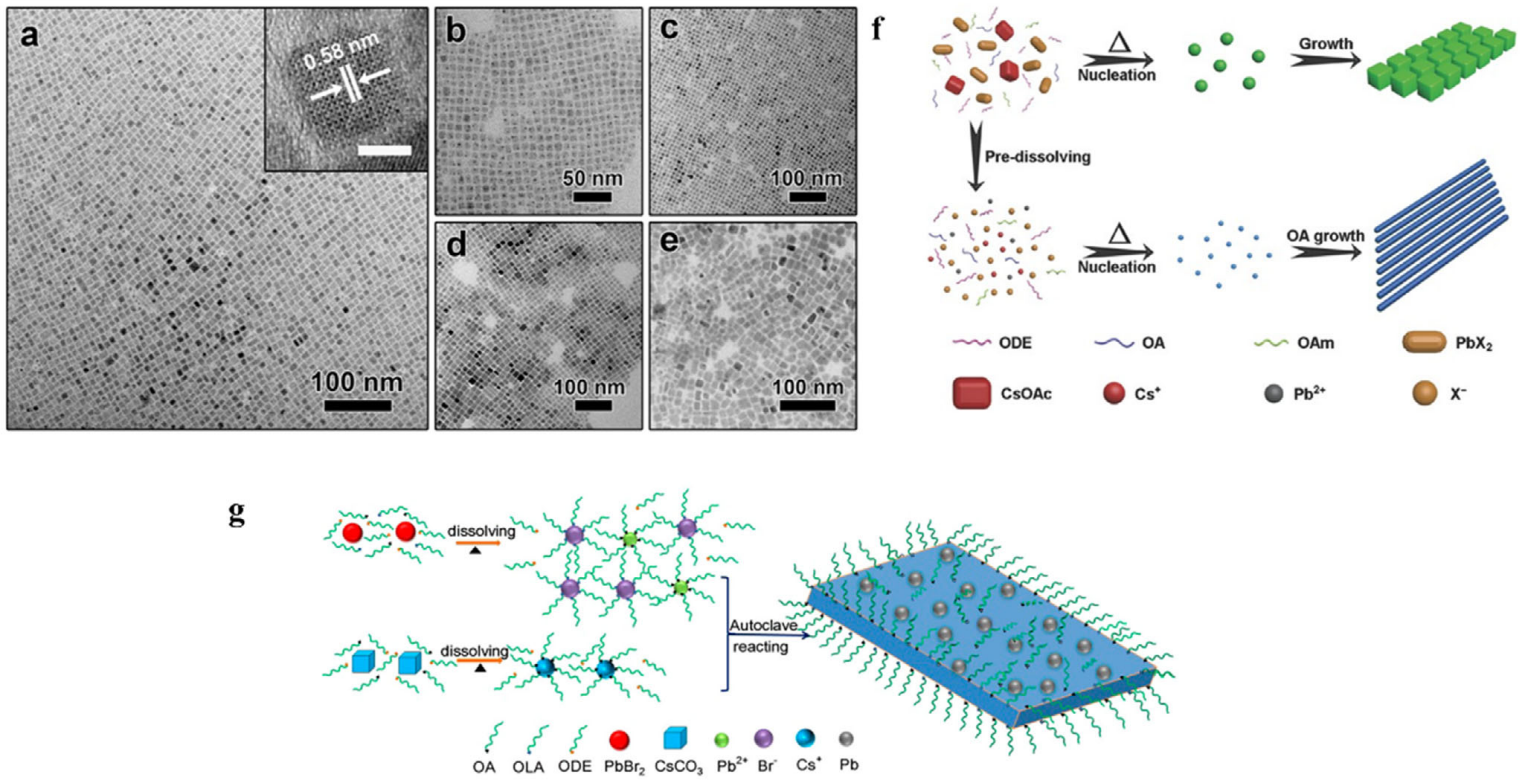
TEM images of a) CsPbBr_3_, b) CsPbCl_3_, c) CsPb(Cl/Br)_3_, d) CsPb(Br/I)_3_, and e) CsPbI_3_ nanocrystals. The inset in a) is the HRTEM image of CsPbBr_3_ nanocrystals. The scale bar in the inset is 5 nm. f) The proposed growth process of CsPbBr_3_ NCs without and with redissolving step. Reproduced with permission.^[^ ^110^ ^]^ Copyright 2017, Wiley‐VCH. g) Diagram of the solvothermal method process. Reproduced with permission.^[^ ^111^ ^]^ Copyright 2018, American Chemical Society.

The solvothermal method is a versatile synthetic approach for preparing CsPbBr_3_ quantum dots (QDs) and nanoparticles by transferring precursor solutions into a high‐pressure autoclave and reacting at 100–200 °C,^[^ ^109^ ^]^ where reaction temperature and organic ligand dosage enable precise control over nanostructure morphology and size.^[^ ^110^ ^]^ In 2017, Chen et al. employed this method to synthesize ultrathin, highly stable, and monodisperse CsPbBr_3_ nanowires (diameter ≈2.6 nm) with a simple protocol: precursor solutions containing cesium acetate (CsOAc) and lead halide (PbX_2_) were heated at 160 °C in a autoclave for 30 min, yielding nanowires with photoluminescent wavelengths spanning 410–700 nm, narrow emission half‐widths (12–36 nm), and high photoluminescence quantum yields (**Figure** **4**a–e). These nanocrystals comprise distinct halide compositions: 1) CsPbBr_3_, 2) CsPbCl_3_, 3) CsPb(Cl/Br)_3_, 4) CsPb(Br/I)_3_, and 5) CsPbI_3_.^[^ ^110^ ^]^ A critical discovery was the influence of precursor concentration on crystal morphology (Figure 4f), when precursors were heated without pre‐dissolution, low initial ion concentrations led to gradual dissolution and nucleation at elevated temperatures, forming nanocubes that grew into nanowires with capping ligands over extended reaction times. In contrast, pre‐dissolved precursors generated higher initial ion concentrations, promoting dense nucleation and smaller nanocrystal sizes. This work established a straightforward route to high‐quality CsPbBr_3_ nanocrystals and provided mechanistic insights into morphology control during perovskite synthesis.

Subsequently, Zhai et al. pioneered an inert‐gas‐free solvothermal strategy to fabricate CsPbBr_3_ nanoplates (NPLs), achieving precise tuning of lateral dimensions (while maintaining a constant thickness of ≈4.2 nm, within the quantum confinement regime) by adjusting reaction temperature and time, as illustrated in Figure 4g.^[^ ^111^ ^]^ This approach enabled emission wavelength tuning from 453 to 465 nm, demonstrating finer morphological control compared to earlier studies. Notably, the method facilitated large‐scale production of CsPbBr_3_ NPLs (≈0.192 g per batch), a critical milestone for industrial manufacturing of all‐inorganic perovskites. By eliminating the need for protective atmospheres and optimizing reaction kinetics, this work underscored the scalability and practicality of solvothermal method, bridging the gap between laboratory‐scale synthesis and commercial applications. The studies collectively highlight the solvothermal method as a powerful tool for tailoring CsPbBr_3_ nanostructures with precise control over optical, structural, and morphological properties, essential for advancing their use in optoelectronic and energy‐related technologies.

### 3.3 Hot Injection Method

The hot injection method is a classic wet‐chemical technique widely used for preparing high‐quality, monodisperse all‐inorganic perovskite CsPbBr_3_ nanocrystals. This approach involves rapidly injecting cesium and lead precursors into a high‐temperature solvent mixture of oleic acid, oleylamine, and octadecene, creating an instantaneous supersaturated environment that promotes rapid nucleation and controlled growth of nanocrystals. By adjusting the reaction temperature, precise control over morphology is achieved: lower temperatures favor the formation of nanoplates, while higher temperatures yield nanocubes.^[^ ^112^ ^]^

**Figure 5:**
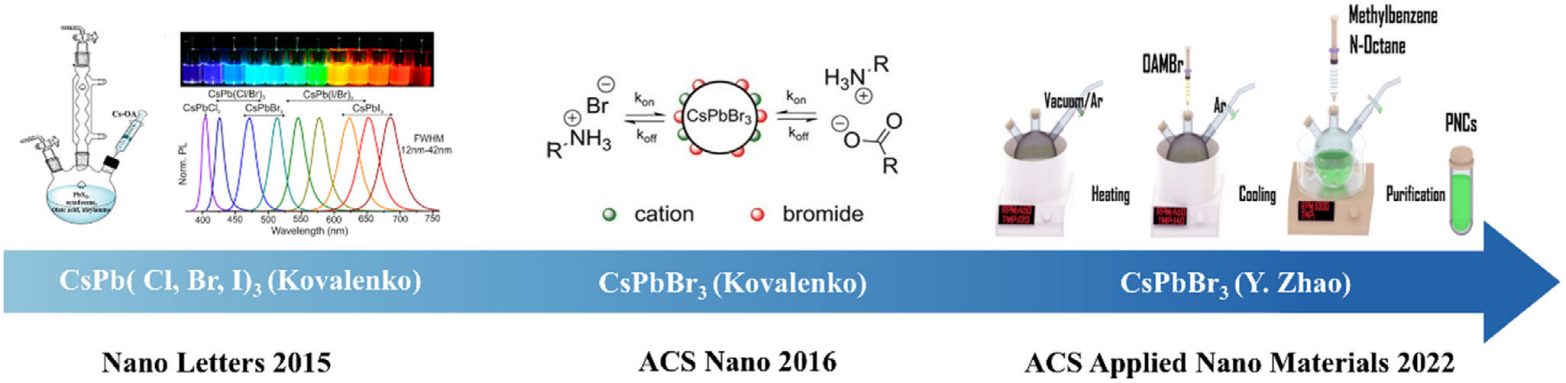
Key milestones in the development of CsPbBr_3_ perovskite synthesis via the hot injection method.

As summarized in **Figure** **5**, key milestones in the development of CsPbBr_3_ perovskite synthesis via the hot injection method highlight the significant progress made over the years. Kovalenko et al. pioneered the hot‐injection method for synthesizing CsPbX_3_ (X = Cl, Br, I) nanocrystals in 2015.^[^ ^113^ ^]^ This method involves the rapid injection of cesium oleate into a heated solution (140–200 °C) containing lead halide and ligands (oleylamine and oleic acid) under nitrogen protection. This method produced size‐uniform nanocrystals with tunable emission wavelengths spanning 410–700 nm via halogen composition adjustment, alongside high photoluminescence quantum yields (PLQYs, 50%–90%) and narrow emission bandwidths (12–42 nm). These findings established the hot injection approach as a foundational technique for all‐inorganic perovskite nanocrystal synthesis.

Subsequently, in 2016, the same group refined the protocol to generate more uniform cubic‐phase CsPbBr_3_ nanocrystals (≈8.4 nm) with enhanced optoelectronic properties, achieving a significant PLQY increase to 83%.^[^ ^114^ ^]^ Key advancements included using oleic acid (OA) and oleylamine (OAm) as dual ligands to dissolve metal halide precursors effectively and maintain surface stability through dynamic acid‐base equilibrium during high‐temperature reactions and purification. A critical improvement was the introduction of high‐boiling solvents (e.g., hexane, toluene) for washing nanocrystals, efficiently removing impurities like unreacted precursors or excess octadecene (ODE) and ensuring colloidal stability.

While the traditional hot injection method enables precise nanocrystal synthesis, large‐scale production of monodisperse colloidal nanocrystals remains challenging due to reliance on slow heat transfer and standard cooling procedures. Prolonged cooling leads to continued crystal growth, resulting in broad size distributions, yield loss, and compromised optical/structural integrity—limitations that hinder high‐end optoelectronic applications. Zhao et al. addressed these challenges with a modified hot injection strategy, the “solvent injection quenching” method.^[^ ^94^ ^]^ By rapidly injecting a cold solvent (low‐temperature toluene or n‐octane) after the high‐temperature reaction, intense instantaneous undercooling is induced, drastically increasing nucleation rates and suppressing subsequent crystal growth. This innovative preparation method produces CsPbBr_3_ nanocrystals with a ≈−100% photoluminescence quantum yield, significantly outperforming traditional methods, and achieves a single‐batch yield of 1.153 grams, marking a transformative advancement toward large‐scale production. The technique not only enhances production efficiency but also preserves nanocrystal quality, overcoming long‐standing bottlenecks in industrial‐scale synthesis and unlocking practical applications in optoelectronic devices. Collectively, these studies demonstrate the versatility of the hot‐injection method in tailoring CsPbBr_3_ nanocrystals, enabling precise control over their morphology, optical properties, and scalability.

### 3.4 Room‐Temperature Ligand‐Assisted Reprecipitation Method (LARP)

**Figure 6:**
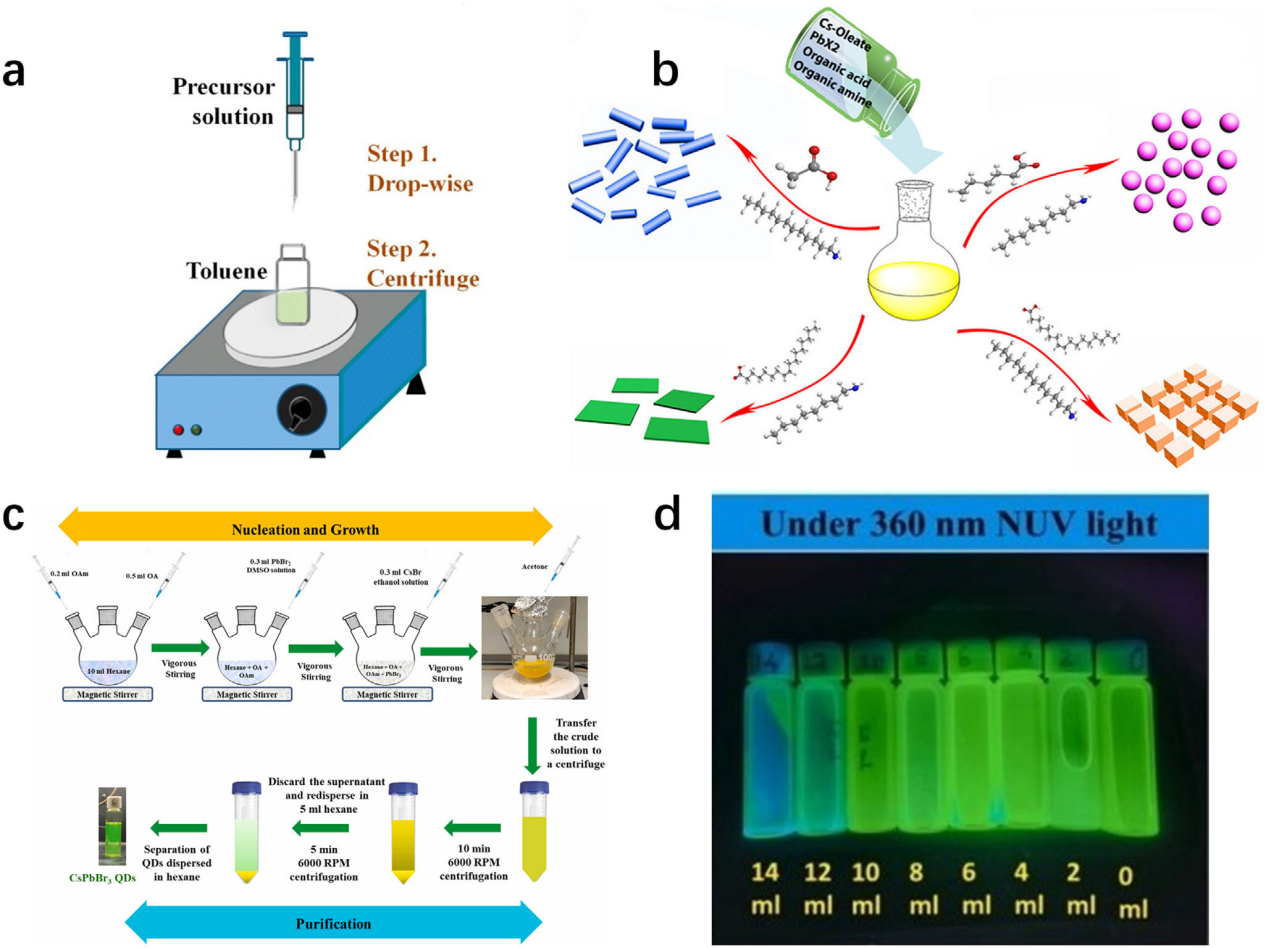
a) Schematic of LARP method (inset: starting materials in the precursor solution). Reproduced with permission.^[^ ^94^ ^]^ Copyright 2015, American Chemical Society. b) Tuning the dimensionality of inorganic CsPbX_3_ (X  =  Cl, Br, I) NCs by varying the organic ligands used. Hexanoic acid and octylamine for spherical QDs; oleic acid and dodecylamine for nanocubes; acetate acid and dodecylamine for NRs; oleic acid and octylamine for few‐unit‐cell‐thick NPLs. Reproduced with permission.^[^ ^117^ ^]^ Copyright 2016, American Chemical Society. c) Schematic of the Emulsion LARP depicting the basic steps involved in the synthesis of colloidal CsPbBr_3_ NCs. d) Photographs of different samples of CsPbBr_3_ NCs taken under 360 nm UV light. Reproduced with permission.^[^ ^118^ ^]^ Copyright 2022, Elsevier.

While hot injection and thermal‐solvent methods produce high‐quality CsPbBr_3_ nanocrystals with excellent optical properties, they often require high temperatures (up to 200–300 °C), strict inert atmospheres, and precise control over reaction kinetics, limiting reproducibility in large‐scale production. In contrast, the room‐temperature ligand‐assisted reprecipitation (LARP) method offers distinct advantages for commercial synthesis, featuring simplicity, efficiency, and operability under ambient conditions without specialized equipment or rigorous environmental control.^[^ ^115^, ^116^ ^]^ Additionally, LARP enables rapid reaction kinetics, high yields, and tunable particle size/morphology via ligand concentration and solvent system adjustment (**Figure** **6**b), making it highly adaptable for industrial applications.

As illustrated in Figure 6a, the LARP process involves preparing cesium oleate (Cs‐oleate) and lead bromide (PbBr_2_) dissolved in N, N‐dimethylformamide (DMF), followed by injecting the precursor solution into nonpolar solvents like toluene or 1‐octadecene (ODE) under stirring to form CsPbBr_3_ nanocrystals. Sun et al. first demonstrated this approach in 2016 for synthesizing all‐inorganic perovskite nanocrystals (CsPbX_3_, X = Cl, Br, I), achieving unprecedented control over morphology‐including 0D spherical quantum dots, 1D nanorods, 2D nanosheets, and cubic nanocrystals‐by tailoring organic acid and amine ligands.^[^ ^117^ ^]^ This breakthrough eliminated the reliance on high‐temperature hot injection, overcoming a critical bottleneck for commercial scalability and laying the foundation for practical CsPbBr_3_ nanocrystal applications. However, this method suffered from severe degradation and defects in nanocrystals due to high‐concentration polar solvents like DMF. Nair et al. developed an emulsion‐based LARP strategy (schematic in Figure 6c), leveraging the immiscibility of polar and nonpolar solvents to form a two‐phase emulsion.^[^ ^118^ ^]^ By adding a demulsifier to induce rapid crystallization, this approach significantly improved nanocrystal stability and optical performance. Moreover, adjusting acetone dosage enabled precise tuning of morphology and emission color, addressing the limitations of traditional LARP in photoluminescent color control. Zhu et al. successfully demonstrated the LARP method for fabricating CsPbBr_3_/TiO_2_ heterostructure‐type photocatalysts, using hexanoic acid and octylamine as protective ligands to achieve uniform loading of sub‐10 nm CsPbBr_3_ nanocrystals on TiO_2_ surfaces through heterogeneous nucleation processes.^[^ ^119^ ^]^ These advances highlight the balance LARP achieves between synthetic simplicity, morphological precision, and material quality, making it a promising and cost‐effective approach for the large‐scale preparation of CsPbBr_3_ nanomaterials.

### 3.5 Template‐Assisted Self‐Assembly Synthesis Method

**Figure 7:**
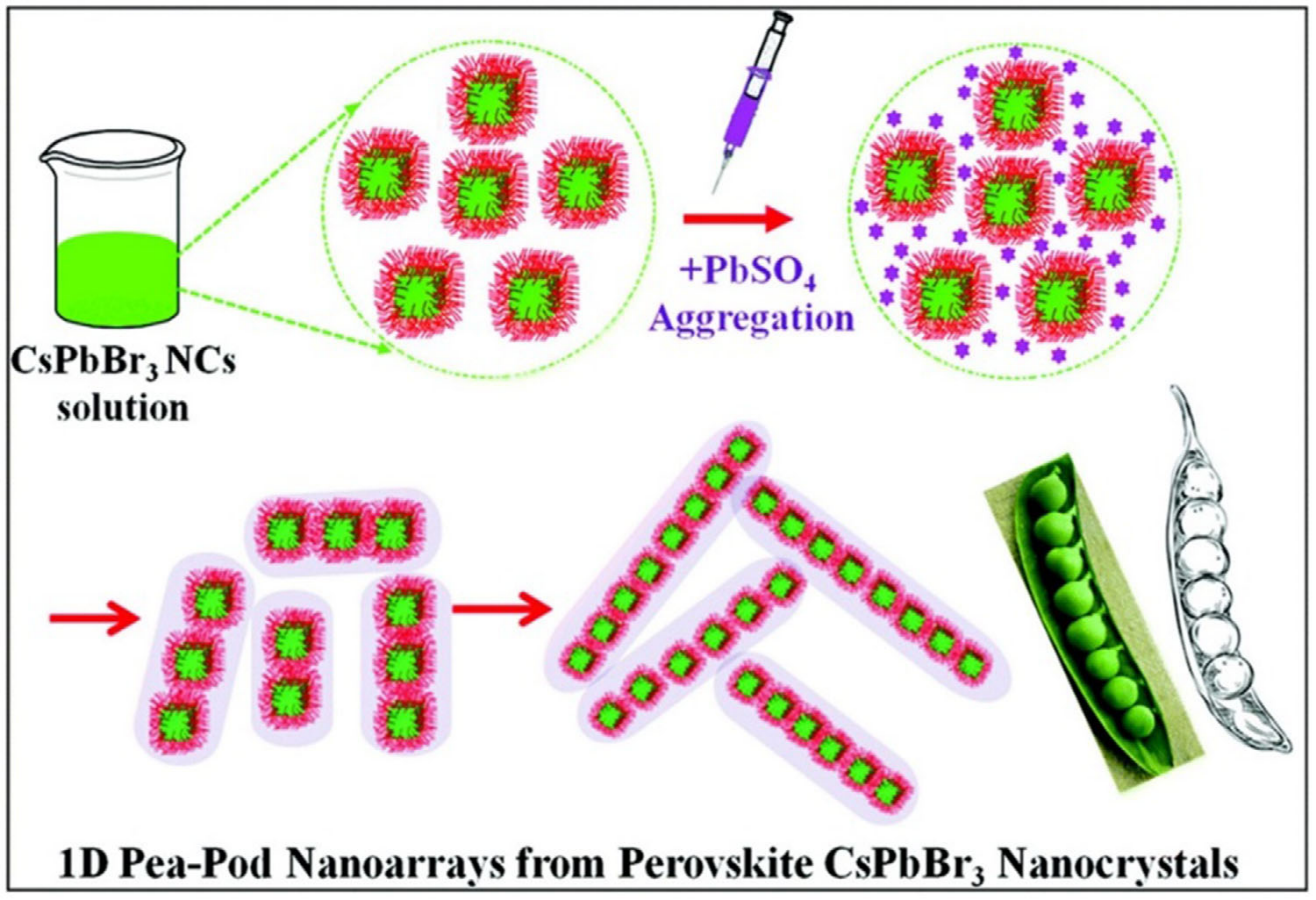
Schematic diagram of 1D pea‐pod like arrays of CsPbBr_3_ NCs embedded in PbSO_4_ shells. Reproduced with permission.^[^ ^122^ ^]^ Copyright 2017, Royal Society of Chemistry.

**Figure 8:**
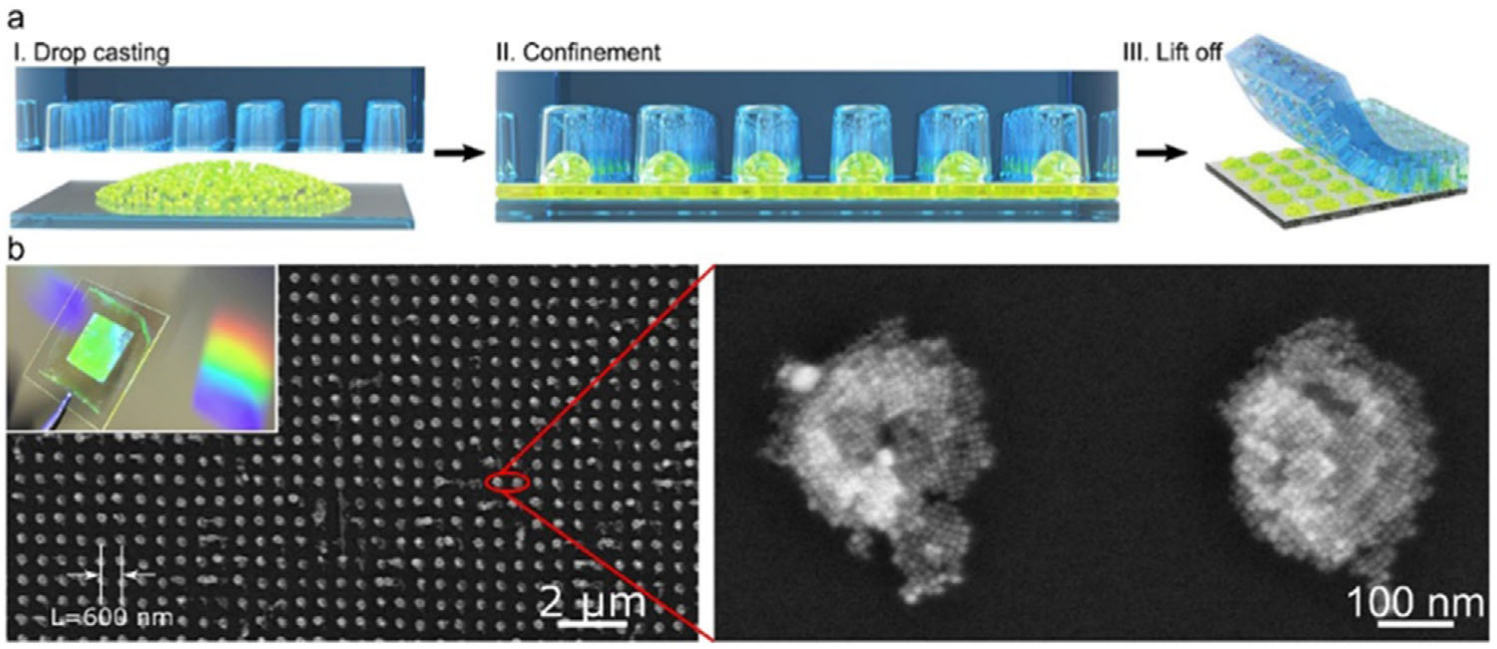
a) Schematic illustration of PDMS template‐assisted fabrication of 2D photonic supercrystals made of perovskite NCs. b) shows CsPbBr_3_ SC arrays on glass substrates with distinct white‐light diffraction, indicating high crystallinity and periodic structure. Reproduced with permission.^[^ ^121^ ^]^ Copyright 2020, Wiley‐VCH.

The template‐guided self‐assembly synthesis of all‐inorganic CsPbBr_3_ perovskites represents a promising strategy for achieving precise control over nanocrystal morphology, dimensions, and hierarchical architectures. By utilizing templating molecules that spontaneously organize into ordered structures under specific conditions, this approach provides a “mold” to confine crystal growth directions and tailor the final nanostructures.^[^ ^120^, ^121^ ^]^ Pan et al. pioneered this method in 2017 by employing PbSO_4_‐shell templates to fabricate pea‐pod‐like CsPbBr_3_ nanoarrays (**Figure** **7**), demonstrating exceptional control over nanocrystal spacing (2.2 nm) and uniformity, while simultaneously enhancing crystallinity and optical properties.^[^ ^122^ ^]^ Building upon this foundation, Liarte et al. advanced the technique by extending 1D superlattices to 2D photonic crystals using polydimethylsiloxane (PDMS) templates (**Figure** **8**).^[^ ^121^ ^]^ Their approach enabled the rapid fabrication of high‐quality 2D photonic crystals over 1 cm^2^ areas, showcasing superior light‐coupling efficiency, tunable photon propagation, and enhanced multiphoton absorption. The scalability and simplicity of this PDMS‐templated self‐assembly method offer a viable pathway for large‐scale production of photonic crystal architectures with optimized optoelectronic performance.

### 3.6 Ultrasonic‐Assisted Synthesis

**Figure 9:**
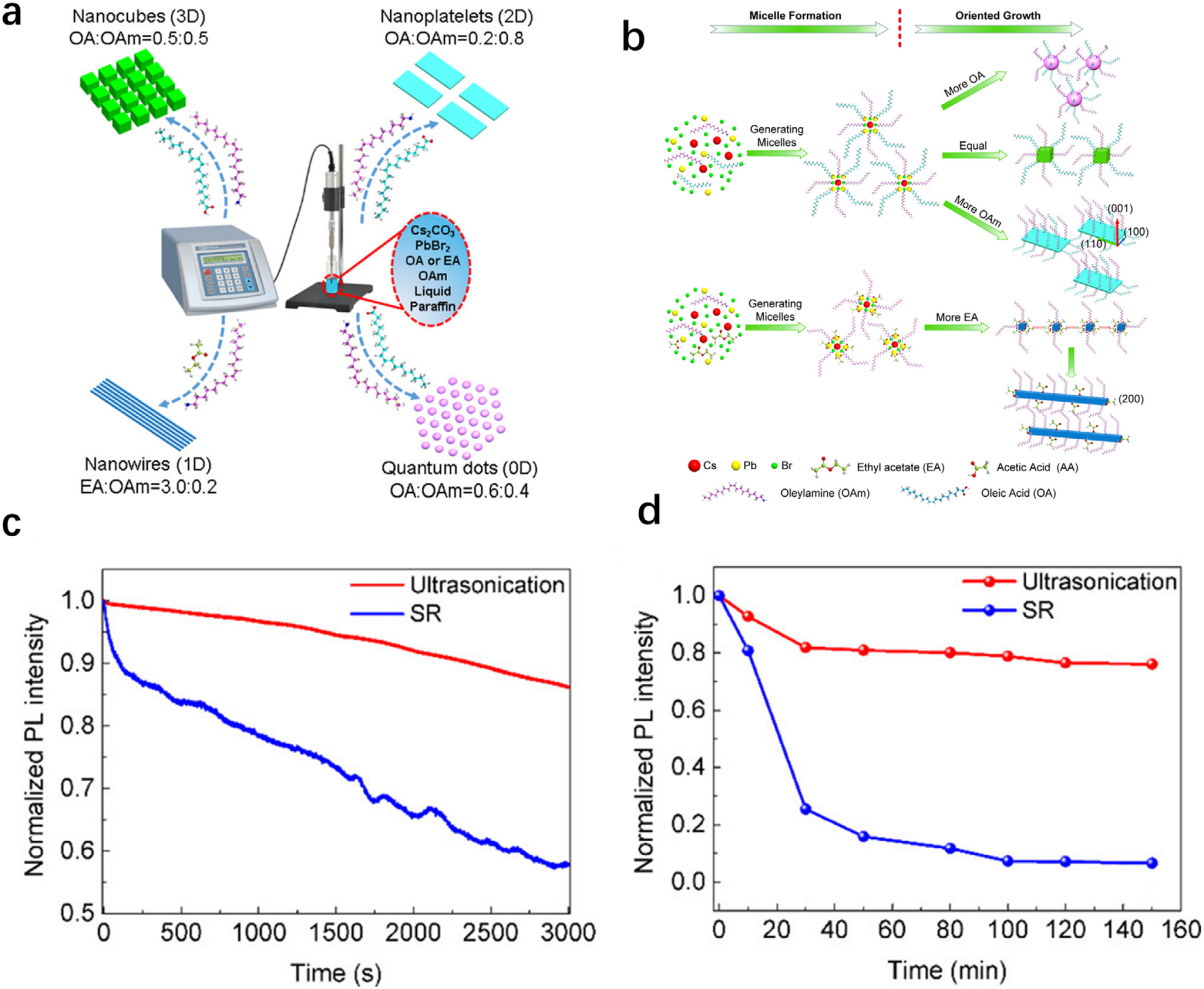
a) Illustration of synthesis of CsPbBr_3_ NCs with different shapes and sizes (3D nanocubes, 2D nanoplatelets, 1D NWs, and 0D Quantum Dots). b) Formation mechanism of CsPbBr_3_ NCs with different shapes and sizes with ligand assistance. c) Photostability of CsPbBr_3_ nanocubes’ colloidal solution under a 365 nm UV continuous excitation. d) Thermostability of CsPbBr_3_ nanocube film under 80 °C heating. Red and blue lines represent the CsPbBr_3_ nanocubes synthesized by ultrasonication and room temperature SR, respectively. Reproduced with permission.^[^ ^125^ ^]^ Copyright 2018, American Chemical Society.

Ultrasonic‐assisted synthesis has emerged as a green and efficient strategy for fabricating all‐inorganic CsPbBr_3_ perovskites. This method leverages cavitation bubbles generated by ultrasonic waves in precursor solutions, whose rapid collapse creates localized high‐temperature and high‐pressure microenvironments.^[^ ^123^ ^]^ These extreme conditions drive bond cleavage and reformation, accelerating dissolution, nucleation, and crystal growth.^[^ ^124^ ^]^ Rao et al. pioneered this approach in 2018 to synthesize CsPbBr_3_ nanocrystals (**Figure** **9**a), achieving precise morphological control from 3D cubes to 2D nanosheets, 1D nanowires, and 0D quantum dots by tuning ligand ratios and ultrasonic parameters.^[^ ^125^ ^]^ The innovative use of liquid paraffin (a mixture of saturated hydrocarbons with formula R─(CH_2_)_n_─CH_3_, where 16 ≤n ≤20) as a non‐polar solvent significantly enhanced the photothermal and chemical stability of nanocrystals through multiple mechanisms. The stability enhancement mechanism involves a comprehensive protective process initiated by the hydrophobic encapsulation of liquid paraffin, which prevents destructive moisture and oxygen infiltration into the ionic perovskite framework. This protective environment is further reinforced through ultrasonic‐induced dehydrogenation reactions that optimize the [Br···N─H^+^] hydrogen bonding network at the nanocrystal surface, establishing a dual‐layer stabilization system that significantly improves thermal and photochemical resistance. For instance, as validated by comparative their rapid and uniform heating provided by microwave radiation accelerates stability tests (Figure 9c,d), 80% photoluminescence intensity was retained after 150 min at 80 °C, significantly outperforming nanocrystals synthesized via room‐temperature supersaturated recrystallization in polar solvents. However, the method introduced excessive organic ligands, which introduced surface defects, compromised optical uniformity, and limited scalability and batch‐to‐batch reproducibility.

**Figure 10:**
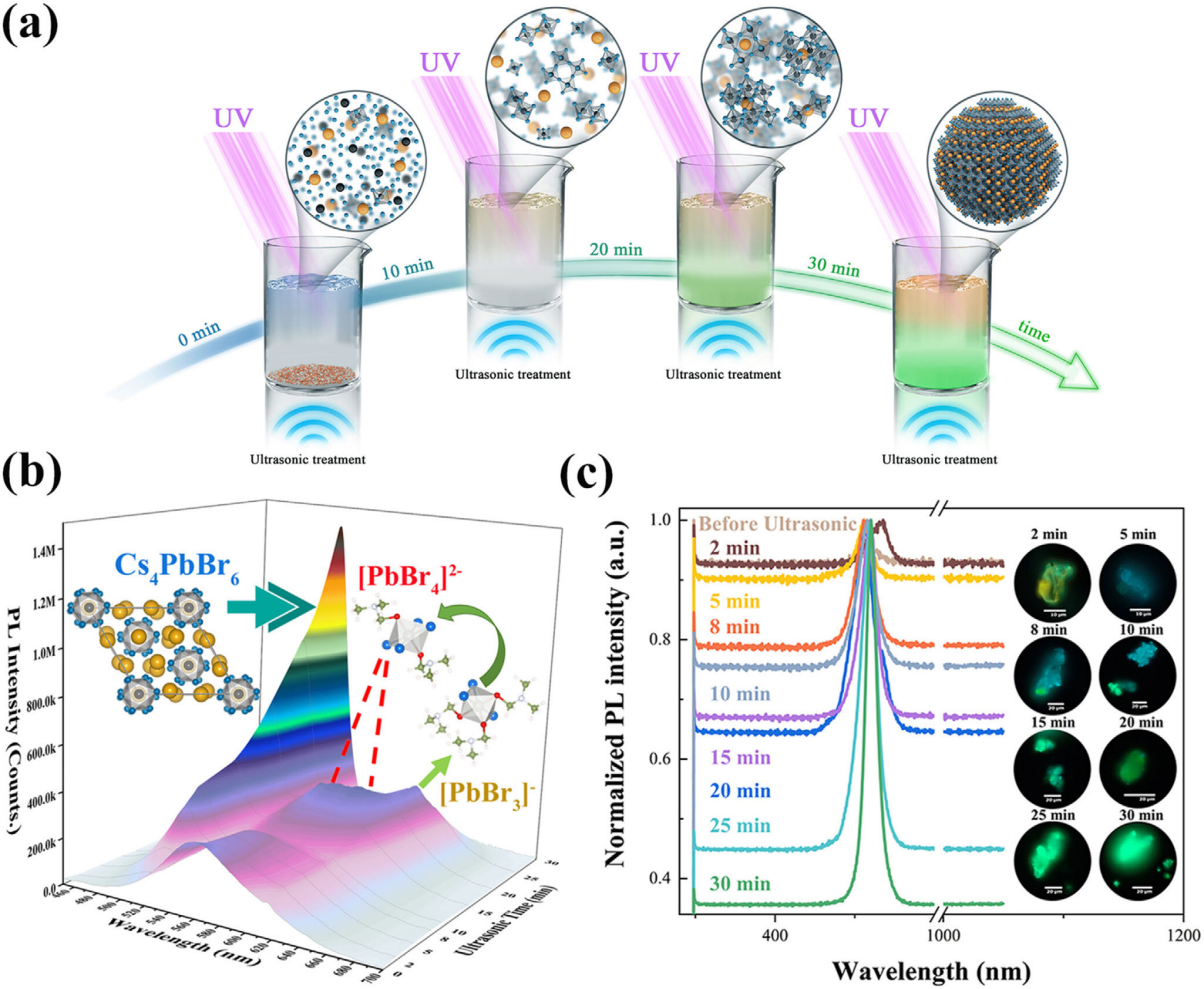
a) Schematic of the ultrasonic preparation process. b) In situ PL spectra of the emulsion with varied ultrasonic time. c) Real‐time monitoring spectra of particle samples extracted from emulsion with the corresponding fluorescence microscope images. Reproduced with permission.^[^ ^126^ ^]^ Copyright 2024, American Chemical Society.

Addressing these limitations, Ning et al. developed a ligand‐free ultrasonic‐assisted synthesis strategy (**Figure** **10**a), directly forming CsPbBr_3_@Cs_4_PbBr_6_ composites.^[^ ^126^ ^]^ This approach eliminated ligand‐induced defects by encapsulating CsPbBr_3_ nanocrystals within an inorganic Cs_4_PbBr_6_ matrix, thereby enhancing stability. Notably, the method enabled the synthesis of a 10 g batch of material in a single run, suggesting potential for scalability. Remarkably, the method enabled single‐batch production of over 10 g of high‐quality material, demonstrating unprecedented scalability and industrial viability. The CsPbBr_3_@Cs_4_PbBr_6_ composites exhibited improved defect tolerance and environmental resilience, overcoming the inherent challenges of conventional ultrasonic‐assisted synthesis.

### 3.7 Microwave‐Assisted Synthesis

**Figure 11:**
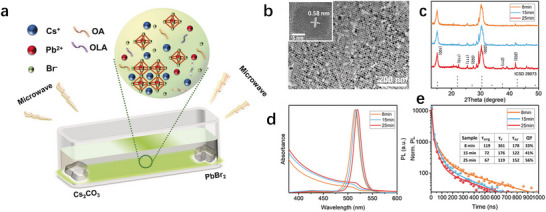
a) Schematic diagram of the microwave‐assisted synthesis method. Characterization of CsPbBr_3_ NCs. b) TEM images of the 25 min sample. Inset: a representative HRTEM image of a CsPbBr_3_ NC with a characteristic lattice plane distance of 0.58 nm. c) XRD patterns of the three samples, as indicated on the frame. Reference card ICSD 29 073 indexes for the cubic phase of CsPbBr_3_ are given for reference. d) Absorption and PL spectra of the three samples, as indicated. e) Time‐resolved PL decays of the three samples fitted with tri‐exponential functions; the inset provides average decay lifetimes (*τ*_avg_), PL quantum yields (QY), radiative recombination lifetimes (*τ*_r_), and effective nonradiative recombination lifetimes (*τ*_nr_). Reproduced with permission.^[^ ^128^ ^]^ Copyright 2018, Wiley‐VCH.

Microwave‐assisted synthesis has emerged as a highly efficient and controllable strategy for producing high‐quality all‐inorganic CsPbBr_3_ perovskites. This method typically involves dissolving Cs^+^ sources and Pb^2+^ precursors in organic solvents, followed by irradiation in a sealed microwave reactor under microwave power ranging from 300 to 800 W.^[^ ^127^, ^128^ ^]^ The rapid and uniform heating provided by microwave radiation accelerates reaction kinetics through dielectric heating, which enables near‐instantaneous temperature ramps.^[^ ^128^ ^]^ This creates a high supersaturation burst of monomers, favoring homogeneous nucleation over heterogeneous pathways.^[^ ^128^, ^129^, ^130^ ^]^ Li et al. pioneered a slow‐paced microwave‐assisted synthesis approach in 2018 (**Figure** **11**a), which allowed real‐time observation of nanocrystal growth dynamics under controlled conditions.^[^ ^128^ ^]^ Their systematic study revealed a three‐stage growth mechanism that begins with the initial formation of bromoplumbate ion frameworks, evidenced by broadened X‐ray diffraction (XRD) peaks and rapid electron‐beam degradation in early‐stage samples due to incomplete structural assembly. This is followed by the gradual incorporation of Cs^+^ ions into interstitial voids within the framework, which is supported by systematic PL lifetime reduction from 119 to 67 ns as trap states are progressively suppressed through Cs^+^ vacancy filling. The process culminates in final crystallization to form robust cubic lattices, confirmed by the emergence of uniform 10 nm cubes in TEM analysis and sharp XRD peaks matching the cubic CsPbBr_3_ structure. The PLQY (Figure 11e) increase from 33% to 56% stems from progressive defect elimination as extended reaction times ensure complete Cs^+^ infilling, converting metastable bromoplumbate scaffolds into defect‐free crystals with reduced non‐radiative recombination pathways. This structural completion is further evidenced by enhanced stability, where 25 min nanocrystals retained 90% PL intensity under light stress compared to complete quenching in shorter reaction samples. However, the extended synthesis duration, while advantageous for laboratory‐scale precision, posed challenges for industrial‐scale production.

**Figure 12:**
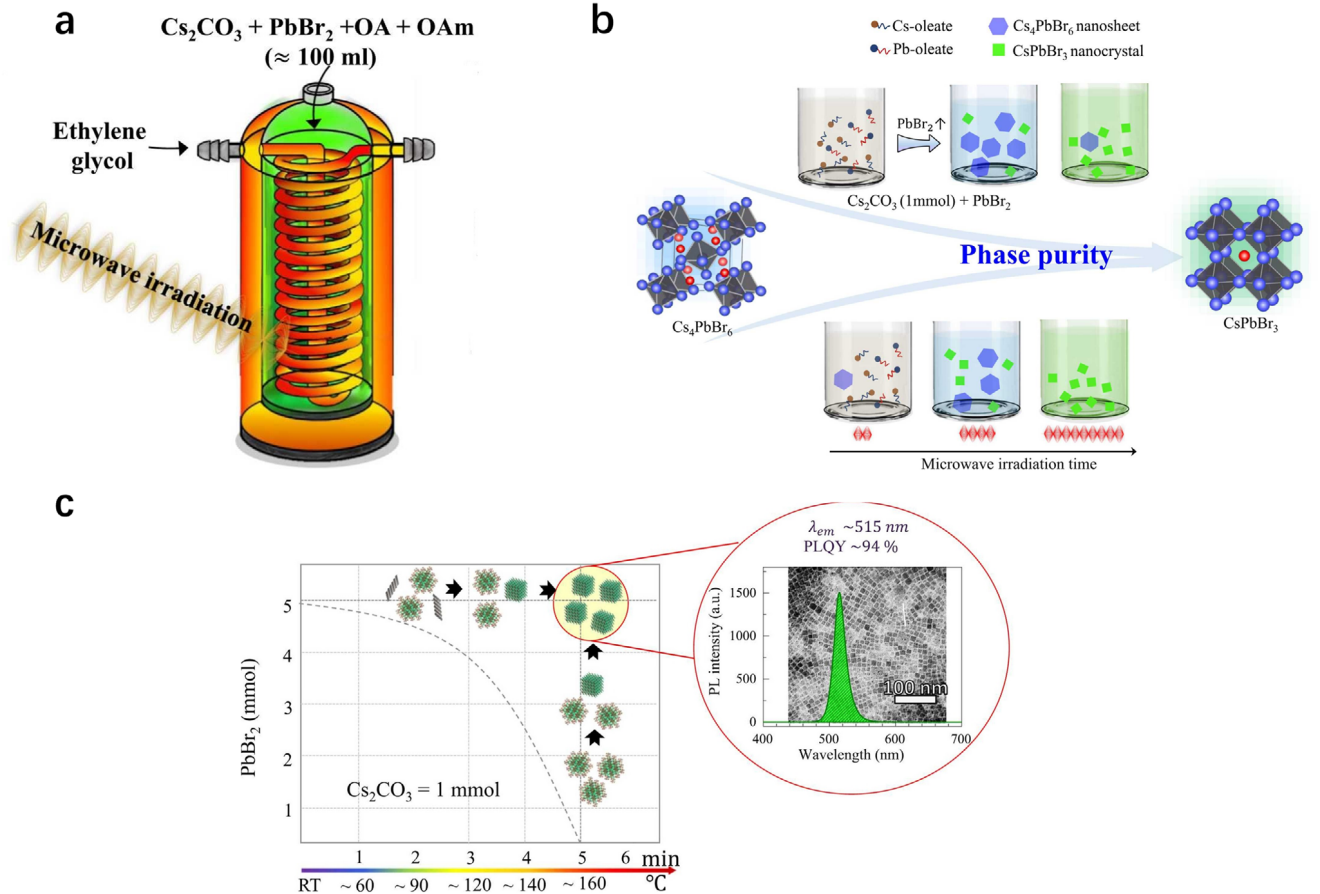
a) Schematic diagram of a specially designed quartz vial. The inner shell contains the reaction mixture. Due to ethylene glycol covering the whole inner shell, the temperature difference across the vial is just 2–3 °C, which is effective for consistent heat transfer in large‐scale NC with uniform size. b) Schematic diagram of the synthesis strategy to mass‐produce uniform and phase‐pure CsPbBr_3_ NCs. c) The transfer results from Cs_4_PbBr_6_ to CsPbBr_3_ with an increase in the Pb precursor and MW irradiation time (800 W). (Inset: TEM images of optimized CsPbBr_3_ NCs and PL spectrum with an emission peak at 515 nm under 365 nm irradiation with a PLQY of 94 %. Reproduced with permission.^[^ ^101^ ^]^ Copyright 2024, Elsevier.

To address these limitations, Cho et al. designed an innovative quartz reactor (**Figure** **12**a) to optimize microwave‐assisted synthesis for scalability and efficiency.^[^ ^101^ ^]^ The reactor features a helical tube structure with dual shells: the outer shell and helical tube are filled with microwave‐absorbing solvents to ensure rapid and uniform heating, while the inner chamber houses the reaction mixture, preventing direct microwave exposure while maintaining temperature homogeneity. This design enabled gram‐scale synthesis of phase‐pure, uniform CsPbBr_3_ nanocrystals. By optimizing precursor ratios, ligand concentrations, and irradiation parameters, the team achieved nanocrystals with a sharp photoluminescence peak at 515 nm (full‐width at half‐maximum of 16.8 nm), a record PLQY of 94% (Figure 12c), and exceptional batch‐to‐batch reproducibility, yielding over 0.16 g per reaction cycle. Compared to conventional methods, this microwave‐assisted strategy demonstrates unparalleled scalability and industrial viability, bridging the gap between laboratory research and large‐scale manufacturing of CsPbBr_3_ perovskites.

### 3.8 Emulsification Synthesis

**Figure 13:**
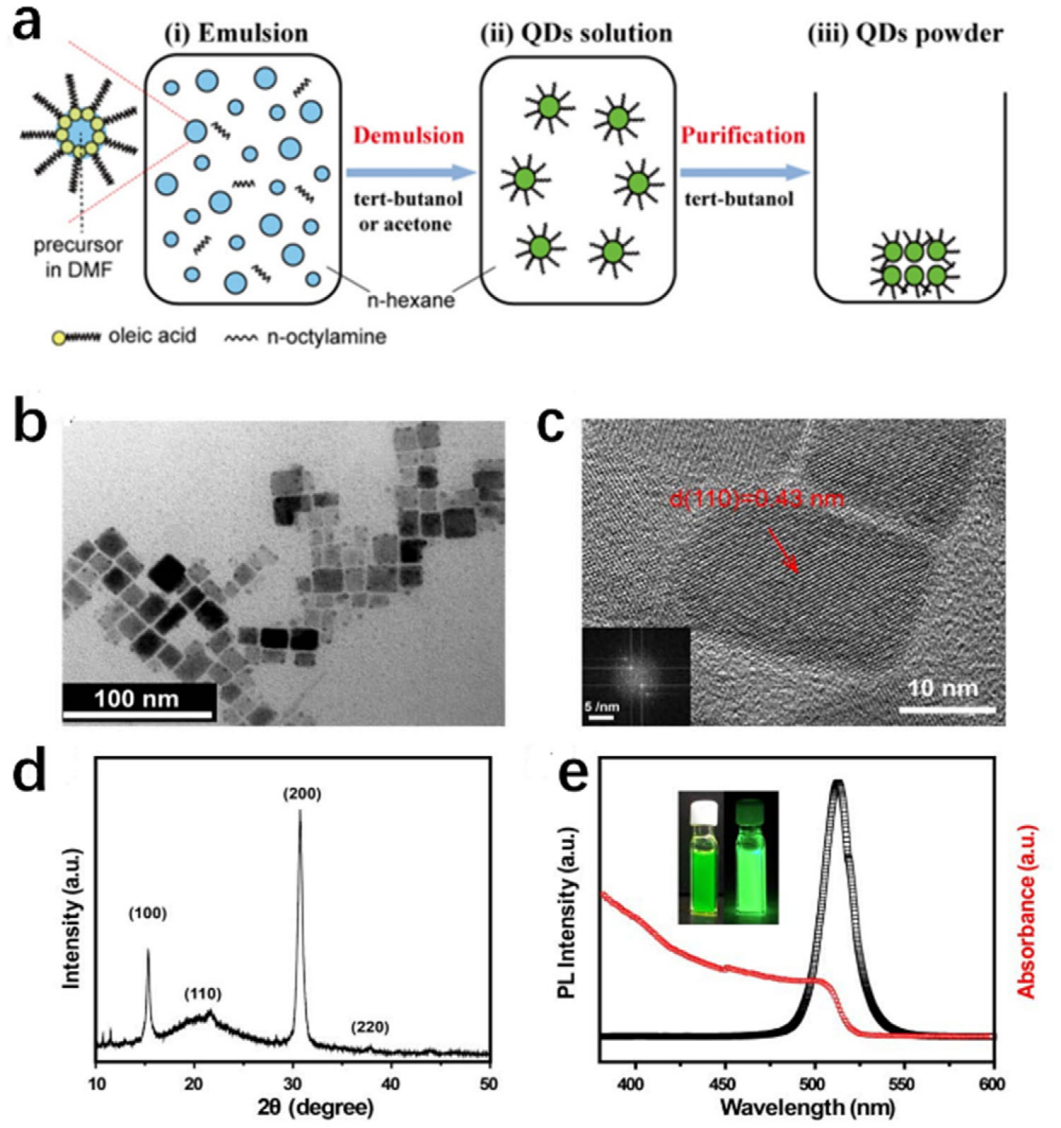
a) Schematic illustration of the QD emulsion synthesis, i) formation of the emulsion, ii) demulsion by adding demulsifier, and redisperse into a colloidal solution, iii) purification into solid‐state powder. b) TEM image and c) HRTEM image of colloidal perovskite QDs. Inset: FFT pattern transformed from the HRTEM image. d) XRD pattern of bulk perovskite QDs. e) UV–vis absorption & PL emission spectra (peak: 521 nm) of a typical perovskite QD solution. Inset: Optical photographs of typical perovskite colloidal solution under the emitterilluminated with ambient light (left) and UV light‐lampcentered at 365 nm (right). Reproduced with permission.^[^ ^133^ ^]^ Copyright 2015, American Chemical Society.

Emulsification synthesis represents a versatile strategy for fabricating CsPbBr_3_ nanocrystals by leveraging interfacial reactions within a biphasic microemulsion system—a thermodynamically stable mixture of immiscible liquids (typically oil and water) stabilized by surfactants, forming nanoscale droplets with a large interfacial area. This approach confines precursor ions at the oil‐water interface, enabling controlled nucleation and growth of CsPbBr_3_ nanocrystals.^[^ ^131^, ^132^ ^]^ Huang et al. pioneered a non‐aqueous emulsion synthesis for perovskite nanocrystals, leveraging a ternary solvent system comprising N,N‐dimethylformamide, n‐hexane, and oleic acid.^[^ ^133^ ^]^ Size tunability was achieved by varying tert‐butanol concentration as the de‐emulsifier. Higher tert‐butanol volumes accelerated solvent mixing, reducing nucleation density and promoting growth‐dominated kinetics, thus enlarging nanocrystal size. The resulting perovskite nanocrystals exhibited monodisperse cubic morphologies and high crystallinity, validated by the HRTEM and XRD results (**Figure** **13**c,d). Optically, they displayed sharp absorption edges (504 nm, 2.46 eV) and narrow emission (510 nm, FWHM ≈18 nm, PLQY: 60%), attributable to suppressed defect states from complete ligand capping during the demulsion process.

**Figure 14:**
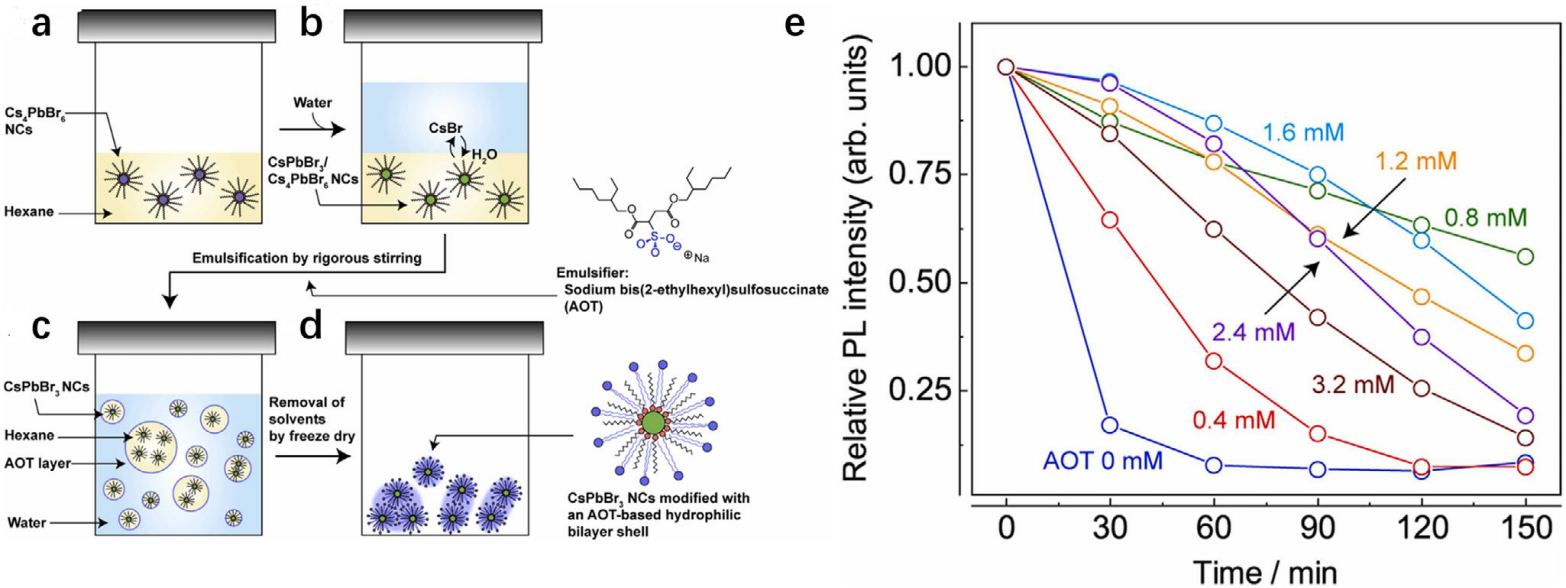
Schematic representation of the preparation of AOT‐modified CsPbBr_3_ NCs: a) Cs_4_PbBr_6_ NC precursor dispersed in hexane. b) phase transition from Cs_4_PbBr_6_ NCs to CsPbBr_3_ NCs by the dissolution of excess CsBr at the hexane/water interface of the emulsion. c) CsPbBr_3_ NC‐encapsulating O/W emulsion. d) AOT‐based hydrophilic bilayer ligand shell bound to the surfaces of CsPbBr_3_ NCs obtained by freeze–drying the prepared O/W emulsion. e) Time evolution of the relative luminescence intensity for AOT‐modified CsPbBr_3_ NCs dispersed in water as a function of the AOT concentration. Reproduced with permission.^[^ ^102^ ^]^ Copyright 2022, Elsevier.

Lu et al. developed a low‐temperature emulsification strategy using sodium bis(2‐ethylhexyl) sulfosuccinate (AOT) to encapsulate CsPbBr_3_ nanocrystals within a hydrophilic bilayer shell (**Figure** **14**).^[^ ^102^ ^]^ The AOT modification enhances chemical stability through the formation of an interdigitated bilayer barrier where hydrophobic chains anchor to native ligands while sulfonate groups provide electrostatic protection against polar solvents. The AOT‐modified nanocrystals retained over 40% of their initial PL intensity after 180 min in water (Figure 14e), compared to complete degradation of unmodified samples within 30 min. Furthermore, the optimized nanocrystals exhibited superior optical properties, including a narrow emission band (515 nm, FWHM ≈18 nm) and a high PL quantum yield (57.2%). This study not only resolved the stability limitations of early emulsification methods but also established a scalable and efficient pathway for synthesizing robust perovskite nanocrystals, unlocking their potential in aqueous photocatalysis, bioimaging, and other polar solvent‐based applications.

## 4 CsPbBr_3_ Perovskite‐Based Heterostructures

### 4.1 Classification of CsPbBr_3_ Perovskite Heterostructures

The design of CsPbBr_3_‐based heterostructures has emerged as a pivotal strategy to address carrier recombination limitations and enhance photocatalytic performance, with three primary configurations dominating the field: Type‐II,^[^ ^134^ ^]^ Z‐scheme,^[^ ^135^ ^]^ and S‐scheme^[^ ^136^ ^]^ heterostructures. These architectures differ in their band alignment mechanisms, charge transfer pathways, and resulting redox capabilities.^[^ ^137^ ^]^ Type‐II heterostructures utilize staggered band edges for spatial carrier separation,^[^ ^138^ ^]^ Z‐scheme systems employ selective interfacial recombination to preserve high‐energy carriers,^[^ ^139^ ^]^ and S‐scheme heterostructures integrate reduction‐ and oxidation‐type semiconductors to optimize both charge separation and redox potential.^[^ ^140^ ^]^ Beyond these established types, emerging configurations such as core–shell heterostructures (e.g., CdS @CsPbBr3^[^ ^141^, ^142^ ^]^ and CsPbBr3@Zeolitic^[^ ^143^ ^]^) and quasi‐Z‐scheme systems^[^ ^144^ ^]^ have shown promise in enhancing stability and energy transfer efficiency through interfacial engineering. Each configuration leverages unique electronic interactions to overcome specific challenges in photocatalysis, such as carrier lifetime, reaction thermodynamics, and material stability.

**Table** **5** summarizes the key characteristics of representative CsPbBr_3_ heterostructure photocatalysts, including synthesis methods, target applications, and performance metrics, providing a comparative overview of recent advancements in this rapidly evolving field.

**Table 5:** Summary of CsPbBr_3_‐based heterostructure photocatalysts.

No.	Sample	Type	Synthesis method	Application	Refs.
1	CsPbBr_3_‐CdS	II	Post‐modified sedimentation method	Ethyl violet reduction	[141]
2	CsPbBr_3_/g‐C_3_N_4_	II	Ligand‐assisted precipitation method	CO_2_ reduction	[52]
3	CsPbBr_3_/TiO_2_	II	Sol–gel	CO_2_ reduction	[50]
4	α‐Fe_2_O_3_/Amine‐rGO/CsPbBr_3_	Z	Solvent evaporation deposition	CO_2_ reduction	[145]
5	Ag/CsPbBr_3_/Bi_2_WO_6_	Z	Electrostatic self‐assembly	Rhodamine B degradation	[53]
6	CsPbBr_3_@TiO_2_	Z	In situ synthesis	Rhodamine B degradation	[146]
7	CsPbBr_3_/TiO_2_	S	Electrostatic assembly	CO_2_ reduction	[147]
8	CsPbBr_3_/GO‐Pt	S	Solution method	Hydrogen generation	[148]
9	BiOBr/Bi‐CsPbBr_3_	S	Hot injection method	CO_2_ reduction	[54]
10	W_18_O_49_/CsPbBr_3_	S	Hot injection method	CO_2_ reduction and Oxidized toluene	[149]

#### 4.1.1 Type‐II Heterostructures

**Figure 15:**
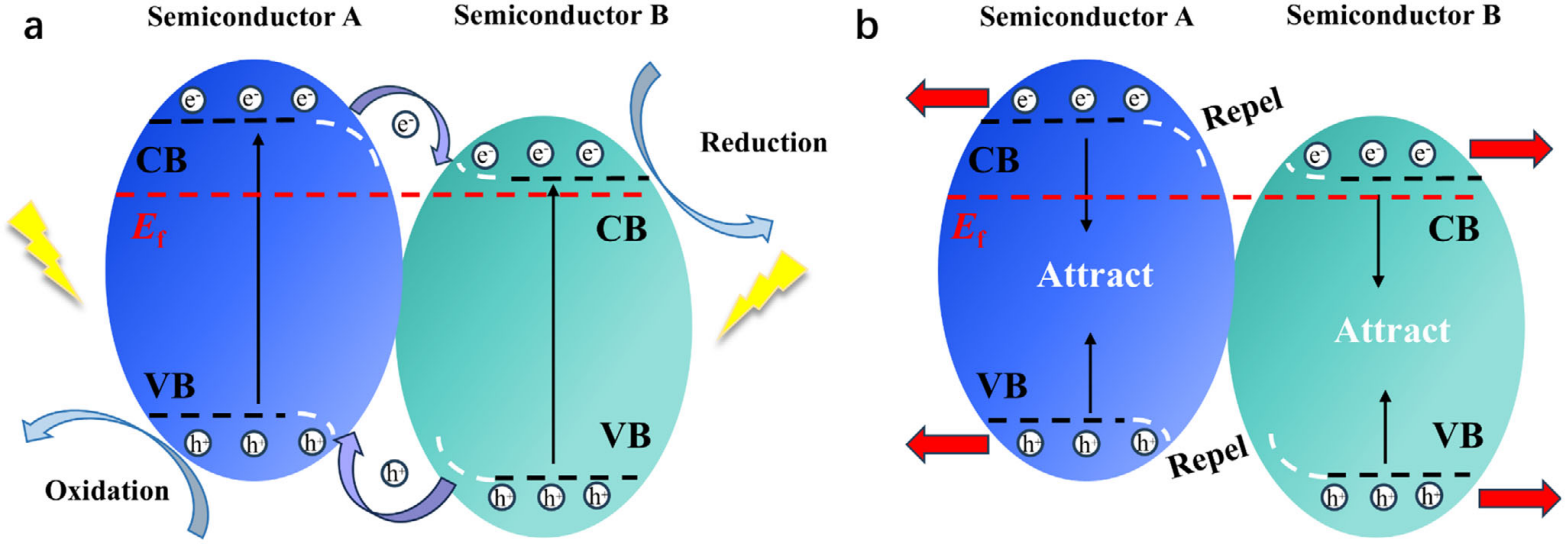
Mechanism and drawbacks of Type‐II heterostructure. a) Type II heterostructure b) like charges repel and opposite charges attract.

Type‐II heterostructures enable spatial separation of photogenerated electrons and holes through staggered band alignments between CsPbBr_3_ and complementary semiconductors.^[^ ^150^ ^]^ As illustrated in **Figure** **15**, this configuration involves two semiconductors with interlocked energy levels. In CsPbBr_3_‐based heterostructures combined with oxides or sulfides (e.g., SnO_2_, ZnO, MoS_2_, CdS), photogenerated electrons migrate to the semiconductor with a lower Fermi level, while holes remain in CsPbBr_3_.^[^ ^151^ ^]^ This mechanism effectively promotes carrier separation, enhances carrier utilization, and reduces energy loss from recombination, significantly boosting photocatalytic efficiency.^[^ ^152^, ^153^ ^]^ The band discontinuity at the heterostructure interface generates a built‐in electric field, which accelerates electron transfer to the CB of the B‐type semiconductor and improves charge transport kinetics. Additionally, the Fermi level difference between semiconductors drives directional carrier migration, establishing a stable charge separation system at the interface. This efficient electron‐hole separation not only minimizes non‐radiative recombination losses but also prolongs carrier lifetimes, drastically enhancing the utilization of photogenerated carriers in photocatalysis.

**Figure 16:**
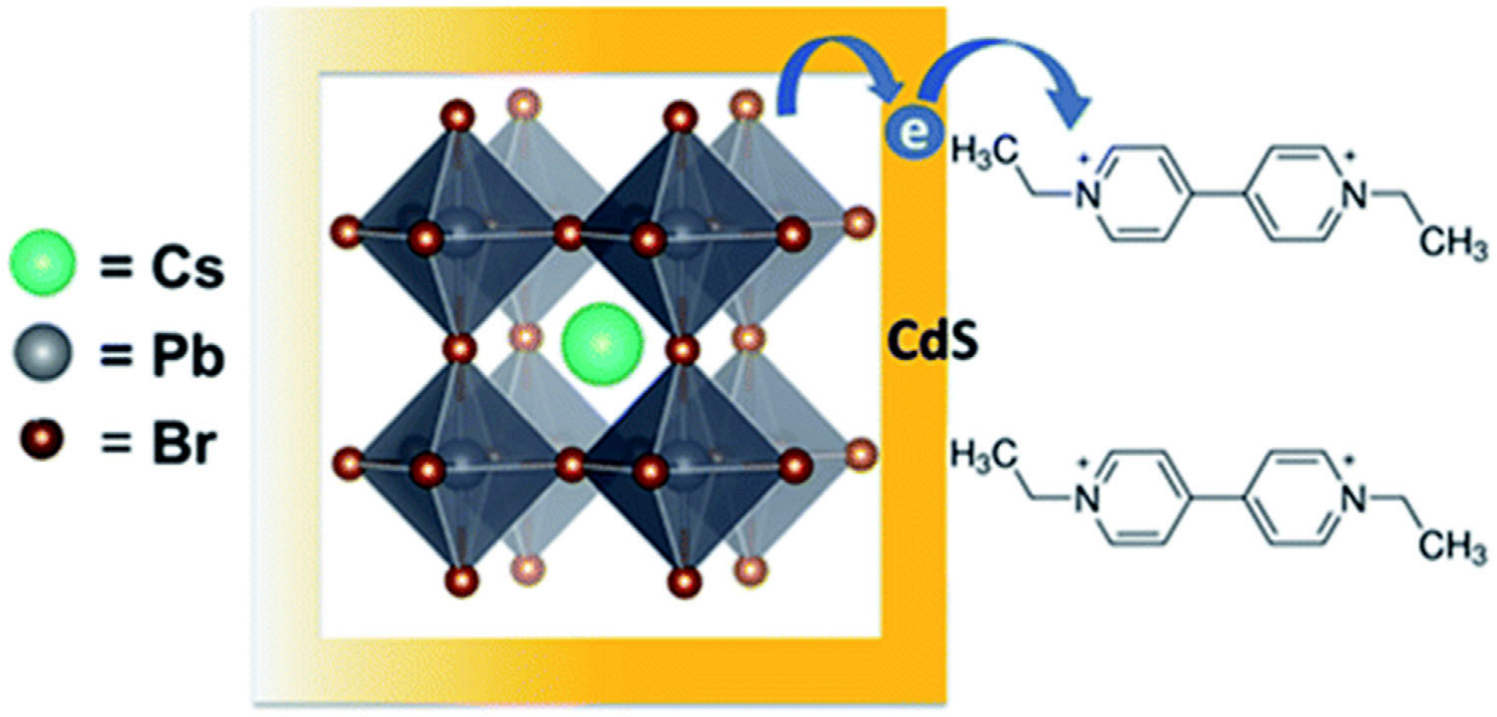
Photoinduced electron transfer between CsPbBr_3_ and viologen mediated by a CdS Layer. Reproduced under the terms of the Creative Commons Attribution 3.0 Unported Licence (CC BY 3.0).^[^ ^141^ ^]^ Copyright 2021, Copyright Kipkorir et al.

**Figure 17:**
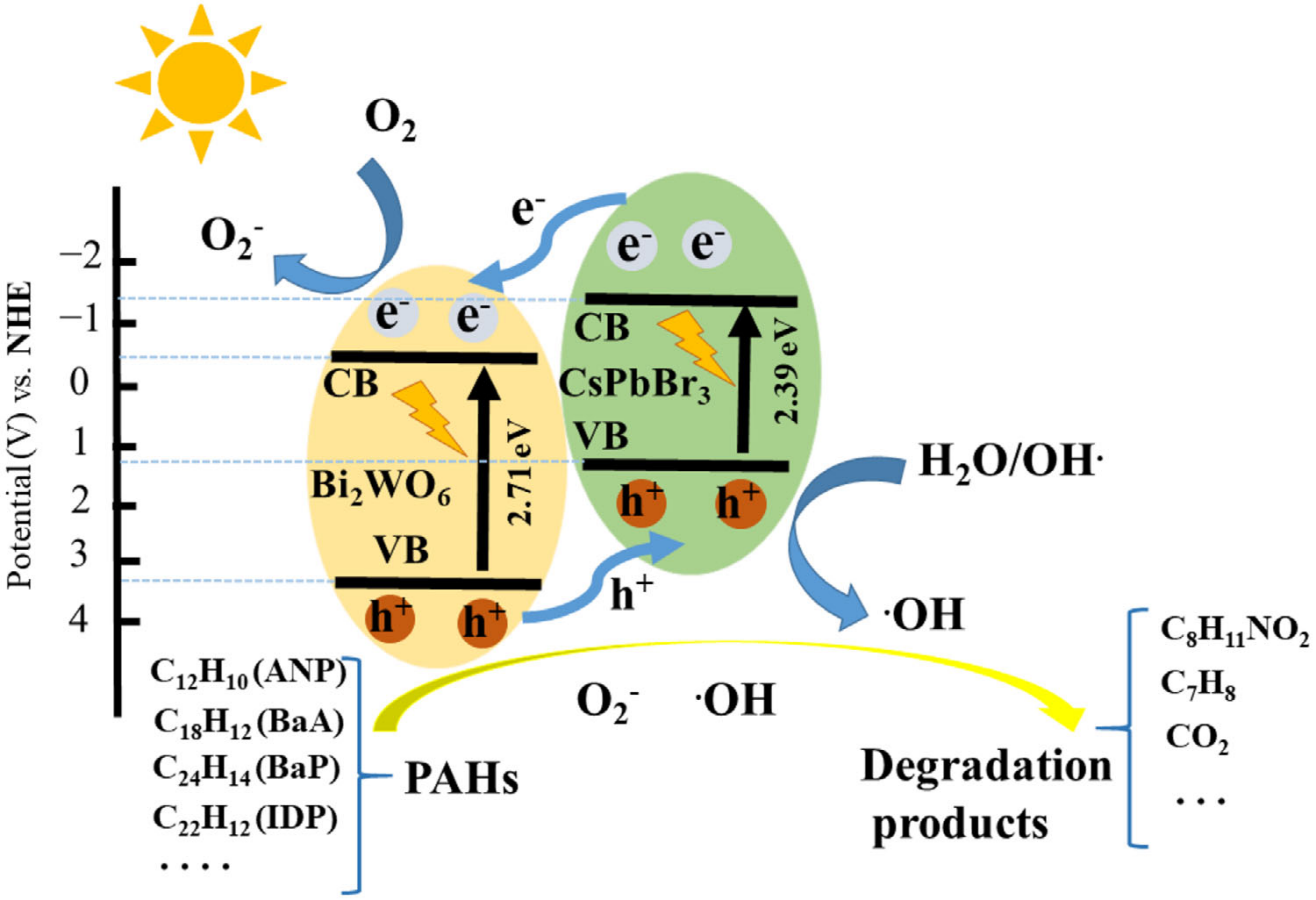
Schematic photocatalytic reaction process and charge transfer of the CsPbBr_3_/BiWO_6_ composite catalyst under solar light irradiation. Reproduced under the terms of the Commons Attribution 4.0 International License (CC BY 4.0).^[^ ^153^ ^]^ Copyright 2021, Copyright Huang et al.

**Figure 18:**
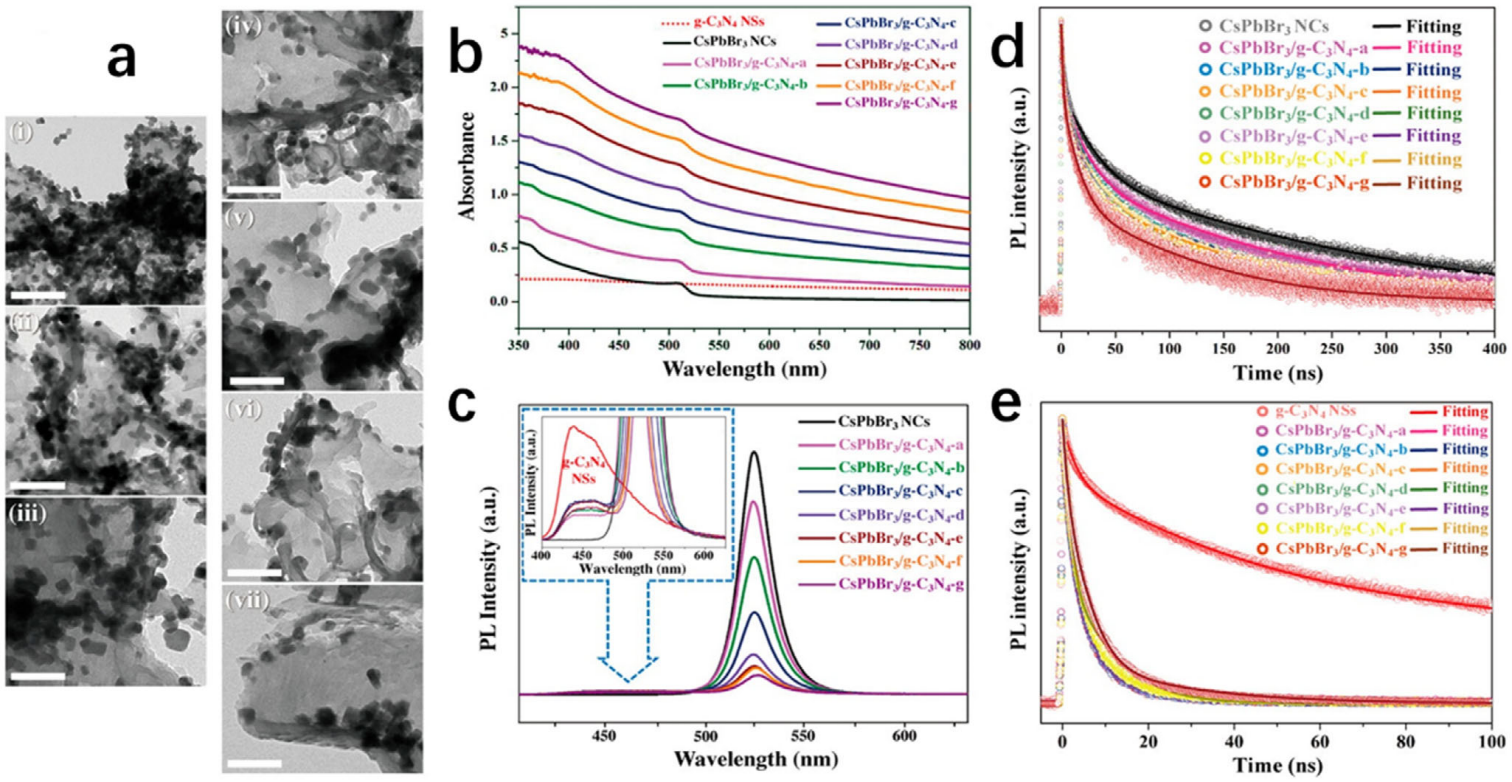
a) TEM images of i) CsPbBr_3_/g‐C3N4‐a, ii) CsPbBr_3_/g‐C_3_N_4_‐b, iii) CsPbBr_3_/g‐C_3_N_4_‐c, iv) CsPbBr_3_/g‐C_3_N_4_‐d, v) CsPbBr_3_/g‐C_3_N_4_‐e, vi) CsPbBr_3_/g‐C_3_N_4_‐f, and vii) CsPbBr_3_/g‐C_3_N_4_‐g (scale bars are 100 nm). b) UV–visible absorption and c) PL spectra of the CsPbBr_3_ NCs, g‐C_3_N_4_ NSs, and CsPbBr_3_/g‐C_3_N_4_ NHS samples. d) TRPL spectra of CsPbBr_3_ NCs and CsPbBr_3_/g‐C_3_N_4_ NHSs at their PL peaks at 525 nm. e) TRPL spectra of g‐C_3_N_4_ NSs and CsPbBr_3_/g‐C_3_N_4_ NHSs at their PL peaks at 440 nm. Reproduced with permission.^[^ ^52^ ^]^ Copyright 2022, American Chemical Society.

Kipkorir et al. reported a Type‐II CsPbBr_3_‐CdS core–shell heterostructure in 2021, where CsPbBr_3_ quantum dots synthesized via hot injection were coated with a CdS shell using chemical deposition.^[^ ^141^ ^]^ This structure leveraged quasi‐Type‐II band alignment to separate photogenerated carriers effectively, suppressing non‐radiative recombination and achieving an 8.4% efficiency in ethyl viologen reduction. The CB of CsPbBr_3_ aligns above that of CdS, facilitating electron transfer to the CdS shell and enabling more efficient carrier separation (**Figure** **16**). Building on similar principles, Huang et al. developed a Type‐II CsPbBr_3_/Bi_2_WO_6_ heterostructure within the same year. By utilizing the significantly higher conduction band potential of CsPbBr_3_ (−1.315 eV) compared to Bi_2_WO_6_ (0.505 eV), they achieved rapid spatial separation of photogenerated charges (**Figure** **17**). Under illumination, electrons migrated promptly from CsPbBr_3_ to Bi_2_WO_6_, while holes transferred in the opposite direction, effectively suppressing electron‐hole recombination. This configuration not only prolongs the lifetime of photogenerated carriers but also significantly enhances the utilization efficiency of active surface sites, facilitating the continuous and efficient generation of reactive oxygen species such as superoxide radicals (·O_2_^−^) and hydroxyl radicals (·OH), particularly hydroxyl radicals (·OH), which facilitated rapid degradation of polycyclic aromatic hydrocarbons (PAHs). The CsPbBr_3_/Bi_2_WO_6_ composite exhibited remarkable photocatalytic performance, achieving over 96% PAH degradation within 50 min of simulated solar irradiation, significantly surpassing the efficiency of pristine Bi_2_WO_6_ (≈32%). These findings further demonstrate the substantial potential of Type‐II heterostructures for improving photocatalytic efficiency and environmental remediation.^[^ ^153^ ^]^ In a separate study, Chen et al. fabricated CsPbBr_3_/g‐C_3_N_4_ Type‐II heterostructures via ligand‐assisted reprecipitation, extending the photogenerated electron lifetime from 32 µs (pure CsPbBr_3_) to 60 µs (**Figure** **18**).^[^ ^52^ ^]^ This prolonged lifetime suppressed carrier recombination, enhancing the photocatalytic CO_2_ reduction efficiency by ≈9 times compared to single‐component CsPbBr_3_. The heterostructure exhibited 100% selectivity toward CO production without byproducts and maintained high activity over multiple reaction cycles, demonstrating excellent long‐term stability. Liu et al. further developed asymmetric CsPbBr_3_/TiO_2_ core–shell structures at the single‐particle level in 2023, using TiO_2_ shells to protect CsPbBr_3_ from moisture‐induced degradation.^[^ ^50^ ^]^ This design reduced charge transfer resistance and enhanced photocurrent responses, highlighting the role of heterostructured interfaces in improving both stability and carrier dynamics.

Despite these advantages, Type‐II heterostructures face challenges such as lattice mismatch and band misalignment between materials, which hinder efficient electron transport and device performance. In addition to lattice mismatch and band misalignment, the inherently low PLQY of Type‐II heterostructures in light‐emitting diode (LED) applications is another significant challenge. The spatial separation of electrons and holes in a Type‐II band alignment reduces their wavefunction overlap, leading to a diminished radiative recombination efficiency.^[^ ^154^ ^]^ This fundamental limitation hampers light emission performance. For instance, Eroglu et al. observed that even strong interfacial bonding and band alignment cannot fully compensate for this overlap reduction, resulting in reduced emission intensity.^[^ ^155^ ^]^ Similarly, Liu et al. used first principles calculations to demonstrate how charge transfer in TypeII van der Waals heterostructures suppresses radiative pathways, further hampering LED performance.^[^ ^156^ ^]^ This conclusion is clearly contrary to the mechanism of improving photocatalytic efficiency. Specifically, LEDs necessitate maximal electron‐hole wavefunction overlap to promote photon generation, whereas photocatalytic applications benefit from prolonged carrier separation, favoring efficient surface‐driven redox reactions. Ultimately, strategic band engineering is essential to tailor carrier dynamics precisely to the demands of the intended energy conversion process. Precise heterostructure construction often requires complex synthesis procedures, increasing production costs and limiting scalability. Thermodynamically, the charge separation efficiency in Type‐II systems is achieved at the expense of reduced redox potential‐photogenerated electrons accumulated in the CB of the B‐type semiconductor possess weaker reducing power, while holes retained in the valence band (VB) of CsPbBr_3_ exhibit diminished oxidizing ability, weakening the thermodynamic driving force for many photocatalytic reactions.^[^ ^137^, ^157^ ^]^ Kinetically, electrostatic repulsion between electrons in the VB of the B‐type semiconductor and holes in the CB of CsPbBr_3_ creates barriers for continuous carrier transfer, complicating charge dynamics and limiting reaction efficiency. These limitations motivate the exploration of Z‐scheme heterostructures, discussed next, as a promising alternative to overcome such challenges.

#### 4.1.2 Z‐Scheme Heterostructures

**Figure 19:**
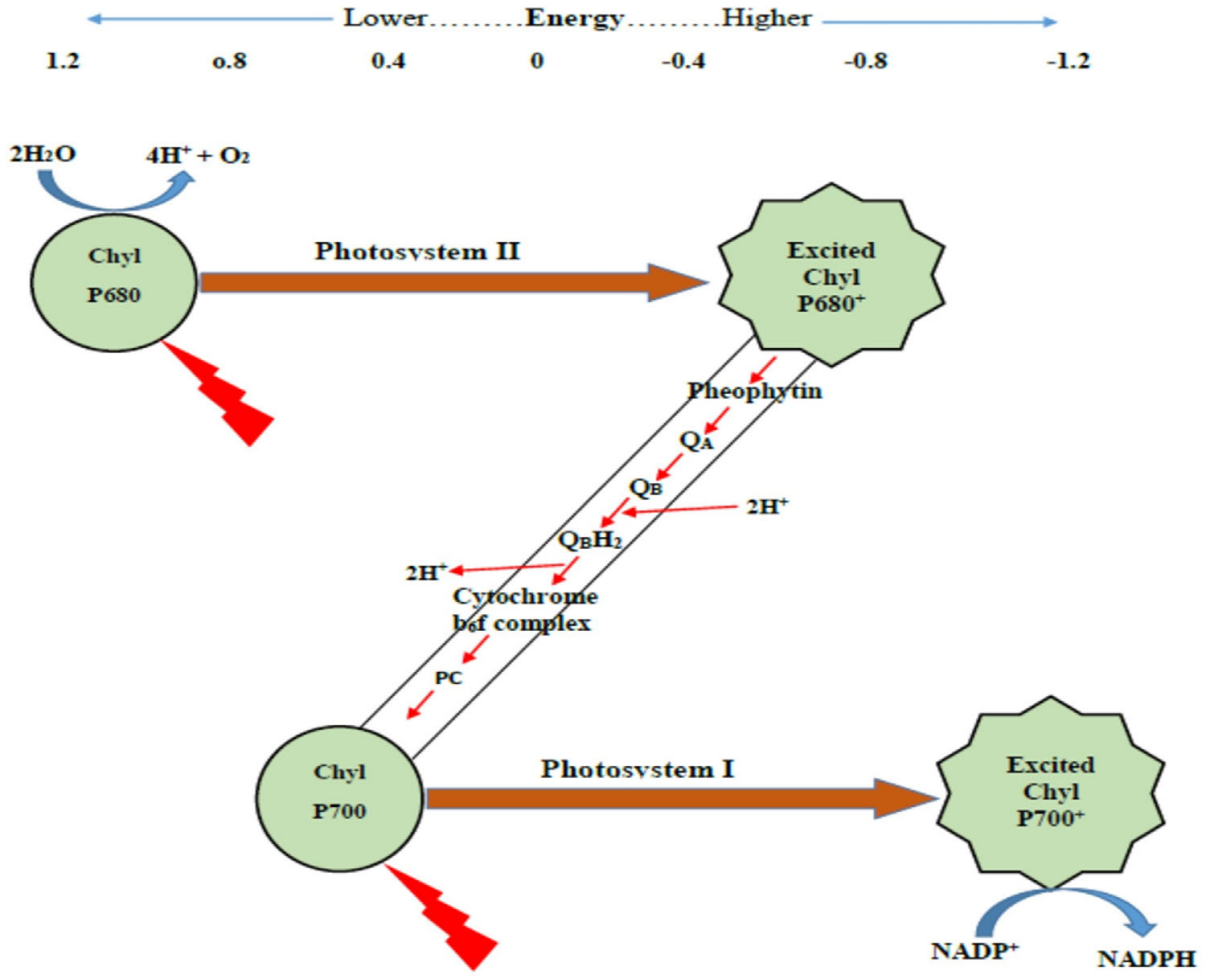
Mechanism of natural photosynthesis. Charge separation and transformation processes, including PS II and PS I. P680: chlorophyll pigment (can absorb 680 nm light), P700: chlorophyll pigment (can absorb 700 nm light). Reproduced with permission.^[^ ^158^ ^]^ Copyright 2019, Elsevier.

The term “Z Scheme” is derived from the electron transfer process in natural photosynthesis^[^ ^158^ ^]^ (**Figure** **19**), which is so named because the path of electron flow resembles the shape of the letter “Z” in English. In green plants, two sequential photoreaction systems (Photosystem II and Photosystem I) absorb light at different wavelengths (around 680 and 700 nm) and work in tandem to drive uphill redox reactions.^[^ ^159^ ^]^ Mimicking this two‐step excitation strategy, artificial Z‐scheme photocatalytic systems were conceived to combine two different semiconductors in series, so that one semiconductor (analogous to PS II) generates high‐energy electrons while the other (analogous to PS I) generates high‐energy holes. Thus, Z‐scheme heterostructures were established as a powerful strategy to replicate the natural photosynthetic Z‐scheme for enhanced redox reactions in artificial photocatalysis.

**Figure 20:**
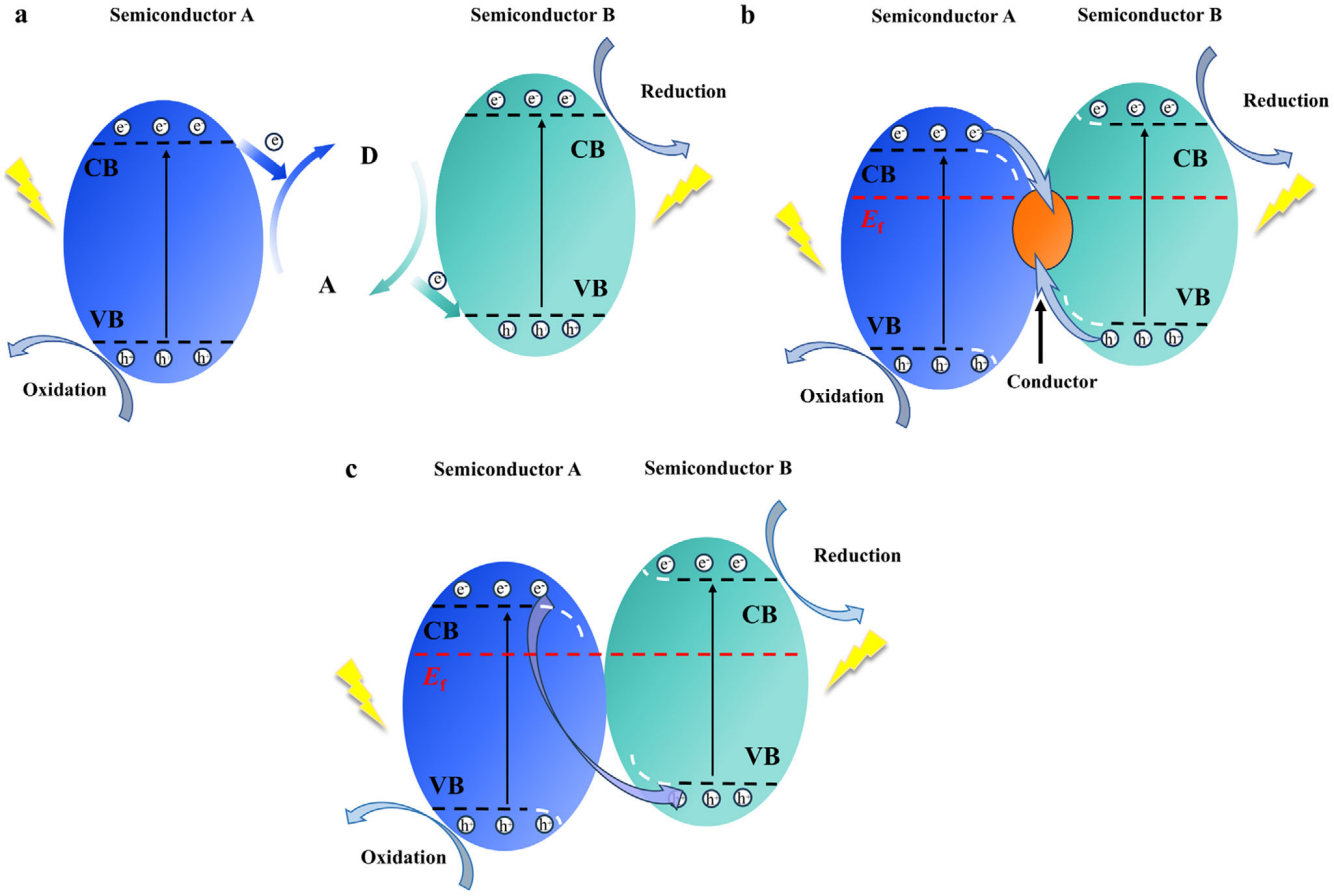
Z‐scheme heterostructure. a) Traditional b) All‐solid‐state c) Direct.

Since their inception, Z‐scheme systems are classified into three generations: traditional (redox‐mediated), all‐solid‐state (conductor‐bridged), and direct (mediator‐free). In a traditional Z‐scheme heterostructure (**Figure** **20**a), the two semiconductor photocatalysts are not in direct contact but are connected through a shuttle redox mediator (typically a reversible electron donor–acceptor pair in solution) Upon illumination, photogenerated holes in the VB of B‐type semiconductor react with electron donors (D), yielding the corresponding electron acceptors (A). Whereas the photogenerated electrons in the CB of A‐type semiconductor react with electron acceptor A, engendering D. Then, the reserved photogenerated electrons in the CB of B‐type semiconductor and holes in the VB of A‐type semiconductor participate in the reduction and oxidation reactions, respectively.^[^ ^135^ ^]^ However, traditional Z‐schemes suffer from drawbacks such as mediator side reactions, light absorption losses, slow diffusion, and instability of redox couples.^[^ ^160^ ^]^ To overcome these issues, the all‐solid‐state Z‐scheme (Figure 20b) was developed, wherein a solid electron mediator (e.g., Au, Ag, reduced graphene oxide) replaces the electron donor–acceptor pair. Under illumination, the photogenerated electrons in the CB of A‐type semiconductor migrate to the solid conductor and further transfer to the VB of B‐type semiconductor.^[^ ^161^, ^162^ ^]^ All‐solid‐state Z‐schemes improve charge transfer kinetics and stability, and they prevent light from being absorbed or scattered by a solution‐phase mediator. However, careful design is required to ensure good electrical contact at interfaces; otherwise, the mediator itself could introduce optical absorption or act as a recombination center if not optimally integrated.^[^ ^163^ ^]^

By contrast, the direct Z‐scheme offers a more integrated and efficient architecture by constructing a tight heterointerface between two semiconductors with staggered band alignments. This configuration allows selective recombination of photogenerated holes from the oxidation photocatalyst and electrons from the reduction photocatalyst at the interface, without relying on any external mediators. Such intimate coupling enhances spatial charge separation, reduces interfacial resistance, and preserves the high redox potentials of both constituents. In this context, direct Z‐scheme heterostructures integrating CsPbBr_3_ perovskites with semiconductors of opposing Fermi level alignments present a particularly promising strategy. The core advantage lies in the selective recombination of photogenerated holes from CsPbBr_3_ and electrons from the partner semiconductor at the interface. This process leaves behind high‐energy electrons in the CB of CsPbBr_3_ and high‐energy holes in the VB of the partner semiconductor, which then participate in reduction and oxidation reactions, respectively (Figure 20c). Such a charge separation mechanism suppresses non‐radiative recombination, prolongs carrier lifetimes, and preserves the intrinsic high redox potentials of both materials, thereby enhancing the thermodynamic driving force for photocatalytic reactions. Additionally, the electrostatic attraction between electrons and holes further facilitates directional carrier migration, with electrons transferring from the CB of CsPbBr_3_ to the VB of the partner semiconductor.^[^ ^164^, ^165^ ^]^

**Figure 21:**
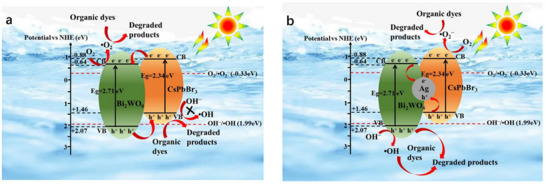
a) Schematic diagram of possible charge separation and photocatalytic mechanism of CsPbBr_3_/Bi_2_WO_6_ composite materials under visible light irradiation. b) Schematic diagram of possible charge separation and photocatalytic mechanism of Ag/CsPbBr_3_/Bi_2_WO_6_ composite materials under visible light irradiation. Reproduced with permission.^[^ ^53^ ^]^ Copyright 2023, Wiley‐VCH.

**Figure 22:**
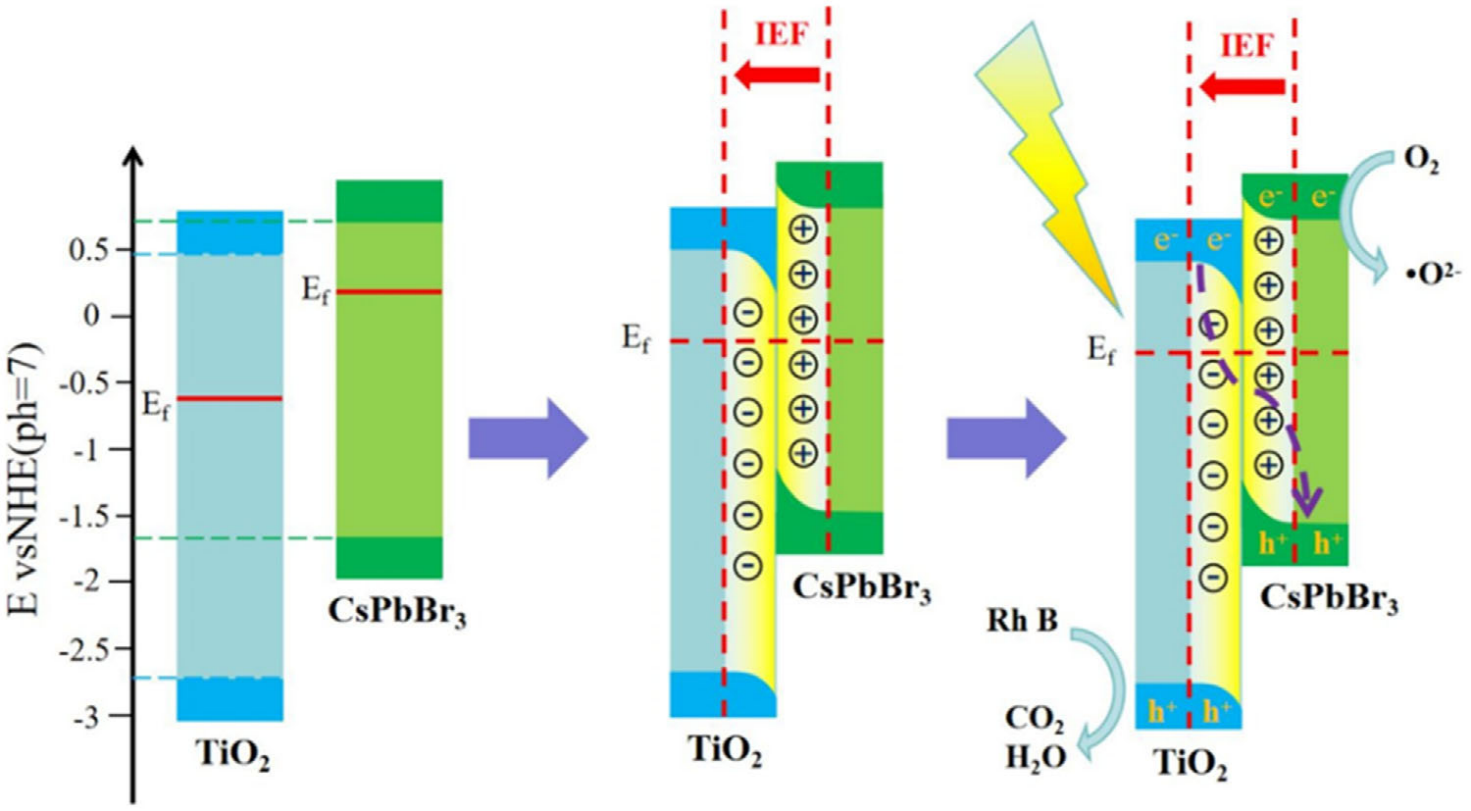
Band structure diagram of CsPbBr_3_@TiO_2_. Reproduced with permission.^[^ ^146^ ^]^ Copyright 2024, Elsevier.

Jiang et al. constructed an all‐solid‐state α‐Fe_2_O_3_/Amine‐rGO/CsPbBr_3_ Z‐scheme heterostructure in 2020, achieving efficient conversion of CO_2_ and H_2_O into CH_4_ and O_2_ under visible light.^[^ ^145^ ^]^ The composite exhibited a total product yield of 469.16 µmol g^−1^ and an electron utilization efficiency of 3132.46 µmol g^−1^. Importantly, it maintained stable performance over 40 h of continuous photocatalysis, demonstrating practical applicability. Xue et al. prepared a composite material composed of 5% Ag and 20% CsPbBr_3_/Bi_2_WO_6_ exhibits a RhB degradation efficiency of 93.9% under simulated sunlight (120 min), which is 4.4 and 1.9 times higher than that of pure Bi_2_WO_6_ and the binary CsPbBr_3_/Bi_2_WO_6_ respectively. This outstanding performance is attributed to the formation of a direct Z‐scheme heterostructure between CsPbBr_3_ and Bi_2_WO_6_, which greatly improves charge separation: in the binary CsPbBr_3_/Bi_2_WO_6_ system (**Figure** **21**a), photogenerated electrons migrate from CsPbBr_3_ to Bi_2_WO_6_ while holes transfer from Bi_2_WO_6_ to CsPbBr_3_, spatially separating the carriers. However, the holes accumulated in CsPbBr_3_ lack sufficient oxidation potential to generate ·OH radicals. Upon introducing Ag nanoparticles as an interfacial charge mediator (Figure 21b), photogenerated electrons from Bi_2_WO_6_ and holes from CsPbBr_3_ recombine on the Ag bridge through a Schottky barrier formed at the metal–semiconductor interface, thereby preserving highly reductive electrons in the CsPbBr_3_ conduction band, which can effectively reduce O₂ to ·O_2_^−^, and strongly oxidative holes in the Bi_2_WO_6_ valence band, which are sufficiently positive to OH^−^ into ·OH. These Ag nanoparticles thus act as charge‐transfer bridges and co‐catalysts that further accelerate carrier separation and reactant activation, ultimately leading to a pronounced synergistic enhancement of photocatalytic RhB degradation efficiency.^[^ ^53^ ^]^ Ding et al. reported a CsPbBr_3_@TiO_2_ Z‐scheme heterostructure in 2024, leveraging mesoporous TiO_2_ encapsulation to enhance stability in aqueous environments, under UV light, and at high temperatures.^[^ ^146^ ^]^ The composite retained 70% of its photoluminescence intensity after two weeks in water (with no Pb^2+^ leakage), 84% after UV irradiation (365 nm, two weeks), and 57% after 120 °C treatment. These outcomes underscore the protective effect of the TiO_2_ shell, which acts as a physical barrier against water ingress, UV‐induced decomposition, and thermal degradation, thereby keeping the embedded CsPbBr_3_ phase active over prolonged periods. Equally important, the Z‐scheme heterostructure architecture in CsPbBr_3_@TiO_2_ led to a substantial boost in photocatalytic activity by enhancing charge separation and utilization. Under visible‐light illumination, the CsPbBr_3_@TiO_2_ catalyst degraded Rhodamine B dye at a rate over five times higher than that achieved with pure TiO_2_. This superior performance is attributed to the effective intra‐heterostructure charge transfer afforded by the Z‐scheme mechanism (**Figure** **22**), which optimizes electron–hole recombination while improving the redox potential of each carrier. The photogenerated electrons are retained in the CsPbBr_3_ component (−0.66 V vs NHE), while the holes accumulate in TiO_2_ (+2.84 V vs NHE). Electrons in CsPbBr_3_ induce the formation of ·O_2_^−^ radicals that oxidize organic pollutants, while holes in TiO_2_ directly oxidize the dye molecules. Collectively, these findings illustrate that the Z‐scheme heterostructure design not only stabilizes the perovskite against environmental stressors but also synergistically enhances charge‐carrier utilization, culminating in markedly higher photocatalytic efficiency than either component alone.

Despite these advancements, Z‐scheme heterostructures face notable challenges. First, significant carrier loss occurs due to interfacial recombination of photogenerated electrons and holes, directly limiting catalytic efficiency.^[^ ^159^, ^166^ ^]^ Second, their design critically depends on precise band alignment: the CB of CsPbBr_3_ must align above the VB of the partner semiconductor to enable electron transfer, a stringent requirement that narrows the pool of suitable materials.^[^ ^137^, ^167^ ^]^ Mismatched band energetics can drastically prevent charge separation and transport efficiency.^[^ ^168^ ^]^ Kinetically, interfacial potential barriers and structural defects often hinder electron‐hole transfer, slowing reaction dynamics.^[^ ^169^ ^]^ Future research should focus on optimizing interface engineering (e.g., defect passivation, ligand modification) and rational band structure design to overcome these limitations, unlocking the full potential of Z‐scheme heterostructures in practical photocatalytic applications.

#### 4.1.3 S‐Scheme Heterostructures

While Type‐II and Z‐scheme heterostructures have contributed significantly to improving charge separation in photocatalysis, they also present notable limitations. Type‐II systems often suffer from weakened redox capacity due to unfavorable band alignments, while Z‐scheme heterostructures require complex synthesis or external mediators to ensure efficient charge transfer. To address these challenges, Yu et al first proposed the concept of S‐scheme heterostructures, offering a new strategy to simultaneously enhance charge separation and maintain strong redox potential.^[^ ^136^ ^]^

**Figure 23:**
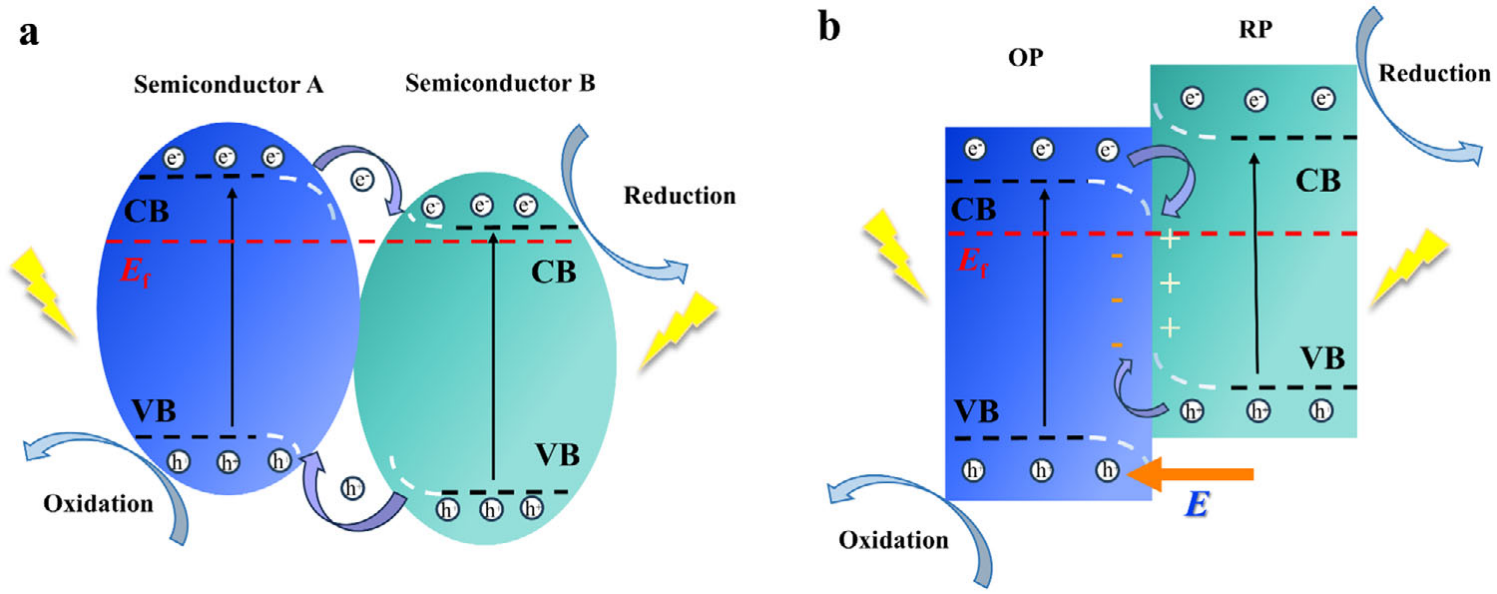
Comparison of charge transfer and band structure between Type‐II and S‐scheme heterostructures a) Type‐II heterostructure b) S‐scheme heterostructure.

S‐scheme heterostructures represent an advanced design integrating both reduction photocatalyst (RP) and oxidation photocatalyst (OP) to optimize both charge separation efficiency and catalytic redox potential. Unlike type‐II heterostructures where electrons and holes migrate to the semiconductor with lower CB and higher VB respectively, leading to reduced oxidation‐reduction capability, S‐scheme heterostructures employ a selective recombination mechanism that preserves carriers with strong redox potentials. As illustrated in **Figure** **23**b, this configuration features RP with higher CB, VB, and Fermi level than OP. In S‐scheme systems, photogenerated electrons accumulate in the CB of RP, while holes remain in the VB of OP, preserving their strong intrinsic redox potentials to drive efficient oxidation‐reduction reactions. The charge transfer pathway resembles a “stepwise jump”, where electrons migrate from the material with a lower Fermi level to that with a higher Fermi level, which is contrary to the traditional type‐II mechanism.^[^ ^170^, ^171^, ^172^ ^]^

**Figure 24:**
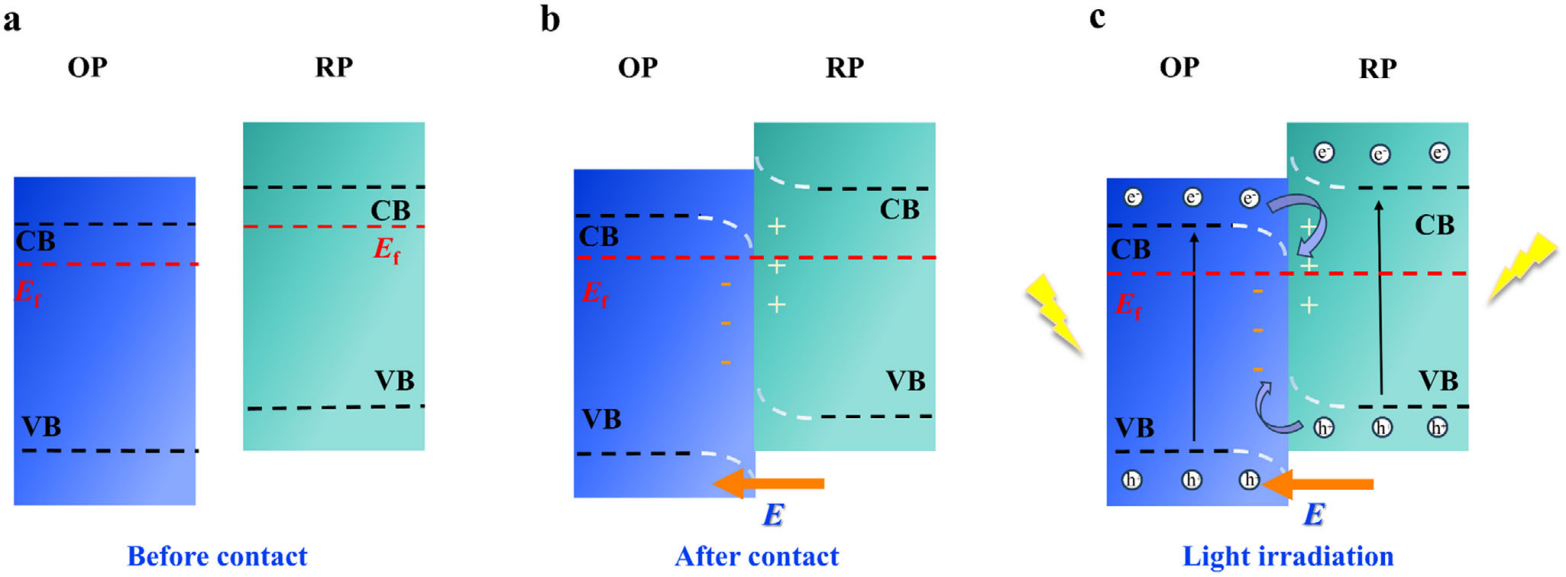
Charge transfer process in S‐scheme heterostructure a) before contact, b) after contact c) light irradiation.

Upon close contact between RP and OP, electrons in RP (with a higher Fermi energy) spontaneously transfer to OP, creating an electron‐depleted layer in RP and an electron‐accumulated layer in OP. This process generates a built‐in electric field at the interface, directed from RP to OP, which accelerates photogenerated electron migration from OP to RP, enhancing carrier separation and suppressing recombination (**Figure** **24**). Concurrently, holes in the RP valence band and electrons in the OP conduction band recombine at the interface via Coulomb attraction, eliminating non‐reactive carriers while retaining high‐energy carriers with robust redox capabilities in both semiconductors. The synergistic effects of the built‐in field, band bending, and Coulombic interaction establish an efficient carrier allocation mechanism, boosting the thermodynamic driving force for photocatalytic reactions.^[^ ^172^, ^173^ ^]^

**Figure 25:**
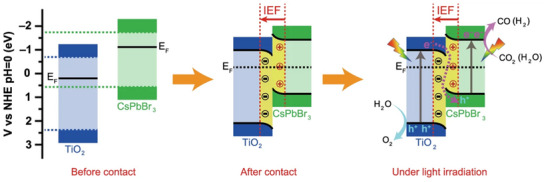
Schematic illustration of TiO_2_/CsPbBr_3_ heterostructure. Reproduced under the terms of the Creative Commons Attribution 4.0 International License (CC BY 4.0).^[^ ^147^ ^]^ Copyright 2020, Copyright Xu et al.

In 2020, Yu et al. reported the first CsPbBr_3_‐based S‐scheme heterostructure, fabricating TiO_2_/CsPbBr_3_ composites via electrostatic self‐assembly to integrate CsPbBr_3_ QDs with TiO_2_ nanofibers (**Figure** **25**).^[^ ^147^ ^]^ The electrostatic self‐assembly process leverages the work function difference between TiO_2_ nanofibers and CsPbBr_3_ QDs, enabling spontaneous electron transfer that creates a built‐in electric field pointing from CsPbBr_3_ to TiO_2_ at the interface. This process ensures intimate contact and establishes the prerequisite energy band bending for S‐scheme charge transfer, while the porous nanofiber structure provides an ideal scaffold for uniform QD anchoring and efficient mass transport. The TiO_2_/CsPbBr_3_ heterostructure achieved a CO_2_ reduction rate of 9.02 µmol g^−1^ h^−1^ (95% CO selectivity) under UV–visible light, which is 93% and 83% higher than pure TiO_2_ (4.68 µmol g^−1^ h^−1^) and CsPbBr_3_ QDs (4.94 µmol g^−1^ h^−1^), respectively. Control experiments also confirmed no CO/H_2_ production in dark or CO_2_‐free conditions, and stability tests showed negligible activity decay after four cycles with preserved structure, further demonstrating the enhanced performance of the S‐scheme TiO_2_/CsPbBr_3_ photocatalyst. Chen et al. advanced this concept in 2022 by designing a CsPbBr_3_/GO‐Pt composite for coupled photocatalytic anaerobic oxidation of aromatic alcohols and H_2_ production.^[^ ^148^ ^]^ Under visible light, the catalyst exhibited a remarkable H_2_ evolution rate (1060 µmol g^−1^ h^−1^) and benzaldehyde (BAD) yield (1050 µmol g^−1^ h^−1^), which are five times higher than those of the CsPbBr_3_‐Pt sample, indicating that the S‐scheme heterostructure can achieve a balance of redox reactions through precise charge carrier management.

**Figure 26:**
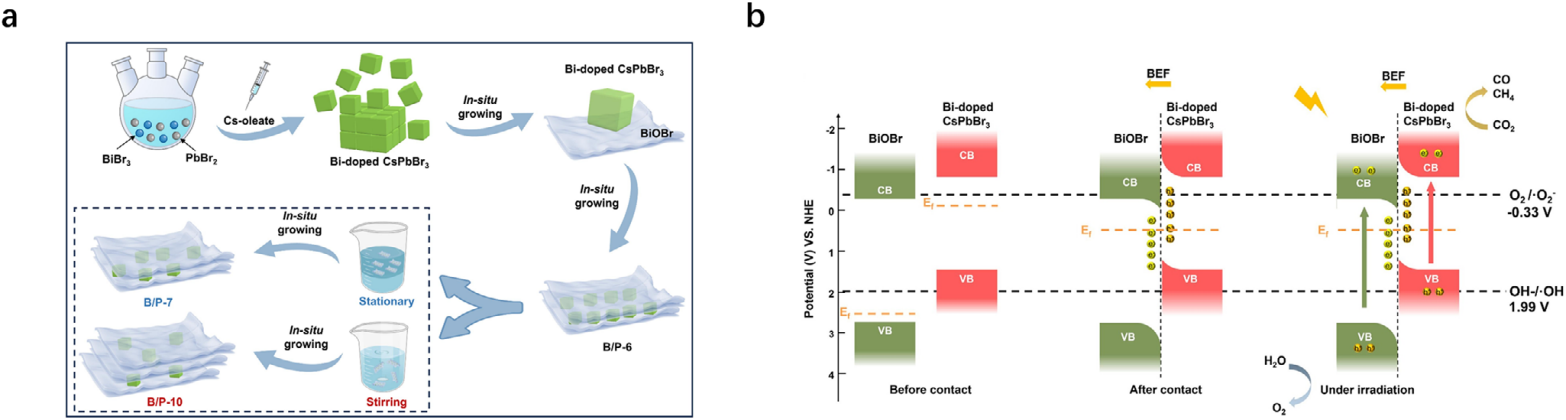
a) Process diagram for preparation of BiOBr/Bi‐doped CsPbBr_3_ (B/P) photocatalysts. b) Schematic diagram of the S‐scheme mechanism of electron transfer for B/P photocatalysts. Reproduced with permission.^[^ ^54^ ^]^ Copyright 2024, Elsevier.

Zhu et al. synthesized a BiOBr/Bi‐doped CsPbBr_3_ S‐scheme heterostructure via one‐step hot injection in 2024, leveraging high‐temperature Bi ion hydrolysis to form tightly bonded interfaces (**Figure** **26**).^[^ ^54^ ^]^ The resulting B/P‐6 catalyst achieved a CO yield of 151.56 µmol g^−1^ h^−1^ with 93.6% selectivity in CO_2_ reduction, which is 3.62 times higher than pure BiOBr, highlighting the role of Bi doping in optimizing band alignment and interface charge transfer.

Despite these advancements, S‐scheme heterostructures face critical challenges: the controlled recombination of low‐energy carriers at the interface requires precise tuning of band positions and material compatibility, a complex task due to the stringent requirement for Fermi level and band edge matching.^[^ ^136^ ^]^ Interfacial defects and potential barriers can hinder efficient carrier transfer, while the rational design of RP/OP pairs remains limited by current material synthesis techniques.^[^ ^137^ ^]^ Future research should focus on developing in situ characterization methods to monitor interfacial charge dynamics and exploring atomic‐level doping/modification to optimize S‐scheme heterostructure performance, bridging fundamental science and practical photocatalytic applications.^[^ ^172^ ^]^

### 4.2 Optimization Strategies for CsPbBr_3_ Heterostructures

The preceding analysis has established that CsPbBr_3_ can be effectively integrated into various heterostructure configurations, including Type‐II, Z‐scheme, and S‐scheme architectures, each offering distinct advantages in addressing its inherent limitations. **Table** **6** provides a comprehensive comparison of the charge transfer mechanisms, redox capabilities, and typical applications of these three heterostructure strategies. However, despite these structural innovations, CsPbBr_3_‐based heterostructures still face challenges that limit their practical photocatalytic applications. Type‐II heterostructures suffer from band mismatch, hampering carrier migration and photocatalytic performance.^[^ ^138^ ^]^ Z‐scheme heterostructures enhance charge separation but face significant interfacial recombination, especially at high loads, affecting long‐term stability and efficiency.^[^ ^164^ ^]^ S‐scheme heterostructures require carefully selected semiconductors with suitable band structures and Fermi levels, are mainly limited to powder photocatalysts, and have issues with carrier recombination and energy release at the heterostructure.^[^ ^136^, ^172^ ^]^ To address these challenges, optimization strategies have been systematically developed with two primary focuses. The first is activity modulation, which involves interface engineering, elemental doping, and porous structure design to enhance charge separation and reaction kinetics. The second is stability enhancement, which includes inorganic encapsulation, ligand passivation, and heterostructure confinement to mitigate degradation.

**Table 6:** Comparative analysis of charge transfer mechanisms and applications in CsPbBr_3_‐based heterostructures.

Characteristic	Type‐II	Z‐Scheme	S‐Scheme
Charge Transfer Pathway	**Electrons**: CB_A_→ CB_B_ **Holes**: VB_B_→ VB_A_	**Electrons**: CB_B_→Mediator→ VB_A_ (recombination) **Retained**: VB_A_ holes + CB_B_ electrons	**Electrons**: CB_OP_→VB_RP_ (recombination) **Retained**: CB_RP_ electrons + VB_OP_ holes
Redox Capability	Weakened	Strong	Strong
Interface Mechanism	**Staggered band alignment**	**Mediator‐dependent charge bridge**	**Built‐in electric field**
Typical Applications	CO_2_ reduction^[^ ^50^, ^52^ ^]^	CO_2_ reduction,^[^ ^145^ ^]^ pollutant degradation^[^ ^53^ ^]^	Water splitting,^[^ ^148^ ^]^ organic synthesis^[^ ^149^ ^]^

#### 4.2.1 Activity Modulation Strategies

Activity modulation strategies for CsPbBr_3_ heterostructures aim to enhance photocatalytic performance by optimizing charge dynamics, light absorption, and reaction kinetics. One of the primary strategies for activity modulation is interface engineering, which lies at the core of enhancing charge transfer pathways. This approach addresses critical challenges in electron transport at the CsPbBr_3_/partner semiconductor interface, primarily by optimizing interface structures to facilitate carrier migration and suppress recombination. By modulating band alignment and reducing interfacial defects, interface engineering enhances charge separation and stabilizes catalytic sites, thereby improving both reaction efficiency and material durability. Specifically, interface engineering can extend light absorption ranges through band bending effects that generate sub‐bandgap states, interfacial state formation that introduces new electronic transitions, and strain‐induced bandgap modulation that shifts absorption edges to longer wavelengths, collectively broadening the spectral response while maintaining efficient carrier extraction.^[^ ^174^ ^]^

**Figure 27:**
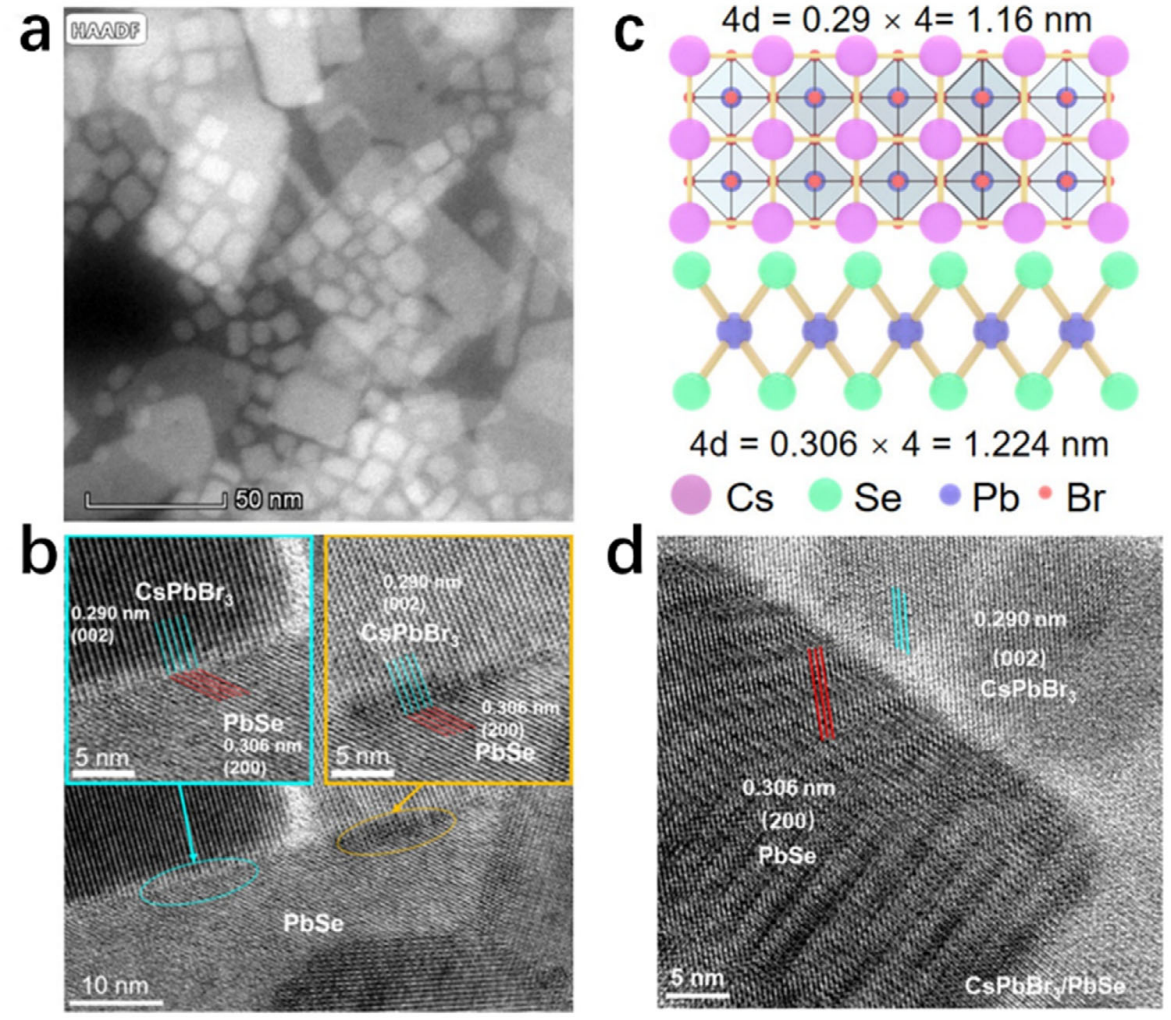
a) High‐angle annular dark‐field TEM image of CsPbBr_3_/PbSe; b) HR‐TEM image of the semicoherent interfaces between purified CsPbBr_3_ QDs and 2D‐PbSe. c) Scheme of the semicoherent interface formed between purified CsPbBr_3_ QDs (blue) and 2D‐PbSe (red), which is directly resolved in (b). d) HR‐TEM images of CsPbBr_3_ QDs/2D‐PbSe without purification. Reproduced with permission.^[^ ^176^ ^]^ Copyright 2022, American Chemical Society.

In 2017, Qian et al. designed two CsPbBr_3_/TiO_2_ heterostructures with distinct interfacial compositions, demonstrating that the CsBr/TiO_2_ interface exhibited stronger visible‐light absorption than PbBr_2_/TiO_2_ due to interfacial state formation and reduced bandgap.^[^ ^175^ ^]^ Time‐resolved photoluminescence measurements revealed a carrier extraction time of 15.33 ns for CsBr/TiO_2_, which is significantly faster than 54.90 ns for PbBr_2_/TiO_2_, highlighting improved charge separation kinetics at the optimized interface. Zhang et al. advanced this concept in 2022 by converting incoherent interfaces in CsPbBr_3_/PbSe heterostructures into semicoherent interfaces via methyl formate (MeOAc) purification of CsPbBr_3_ quantum dots (**Figure** **27**).^[^ ^176^ ^]^ High‐resolution transmission electron microscopy (HRTEM) revealed clear atomically semicoherent interfaces spanning several unit cells, with lattice spacing measurements showing CsPbBr3 ([002] = 0.290 nm) and PbSe ([200] = 0.306 nm) corresponding to a low interfacial mismatch factor of 5.23% (Figure 27b). The semicoherent interface formation was directly evidenced by the intimate lattice matching visible in the HRTEM images, where discrete interfacial dislocations accommodate the small lattice mismatch between the cubic CsPbBr3 and cubic PbSe phases (Figure 27c). This structural refinement enhanced lattice matching, accelerating interfacial charge transfer and reducing electron‐hole recombination. The optimized heterostructure achieved a CO evolution rate of 322.4 µmol g^−1^ h^−1^ in CO_2_ reduction with 91% selectivity, representing nearly a three‐fold enhancement compared to unmodified counterparts, demonstrating the critical role of semicoherent interface engineering in boosting catalytic performance through improved electronic delocalization and efficient charge separation. Deng et al. further exploited interface engineering in 2023 by integrating CsPbBr_3_ quantum dots with Co_3_O_4_ nanocages, introducing oxygen vacancies on Co_3_O_4_ surfaces to enhance interfacial interactions.^[^ ^177^ ^]^ These vacancies provided additional electron traps, optimizing charge separation and enabling rapid electron transfer via Cs─O bond formation. The resultant CsPbBr_3_/Co_3_O_4_ heterostructure achieved CO and CH_4_ yields of 55.82 and 5.98 µmol g^−1^ h^−1^ without the use of photosensitizers or sacrificial agents. This represents a significant improvement over the unoptimized system, suggesting that defect engineering at the interface can facilitate carrier migration to active sites.

**Figure 28:**
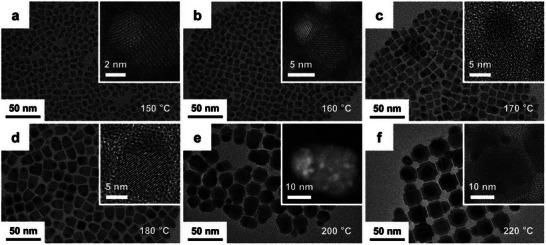
Effect of reaction temperatures on the synthesis of Janus NCs. a–f) TEM images of samples synthesized at different temperatures, ranging from 150 to 220 °C. The insets show the magnified view of a single Janus NC. In all cases, CdS hemispheres grow exclusively on a single facet of CsPbBr_3_. Reproduced under the terms of the Commons Attribution 4.0 International License (CC BY 4.0).^[^ ^38^ ^]^ Copyright 2023, Copyright Qiu et al.

Beyond direct structural optimization, interface modification using conductive materials such as carbon nanotubes, graphene oxide, and conductive polymers is a key strategy for enhancing charge transport.^[^ ^178^, ^179^, ^180^ ^]^ These materials provide additional electron transfer pathways, reducing recombination by facilitating rapid extraction of photogenerated electrons from CsPbBr_3_. For example, Chakraborty et al. incorporated In‐doped CdO into CsPbBr_3_ heterostructures via a digestion‐ripening method, a colloidal synthesis technique that involves the controlled growth and size selection of nanoparticles by thermal treatment in the presence of ligands and solvents. This process enhances the monodispersity of the particles and optimizes their optical properties. The method boosted the PLQY of CsPbBr_3_ quantum dots by 24% through improved carrier recombination pathways.^[^ ^181^ ^]^ The In‐doped CdO layer also enhanced light absorption in the mid‐infrared region (0.43 eV) via local surface plasmon resonance (LSPR), demonstrating dual benefits in both charge transfer and light harvesting. Qiu et al. demonstrated epitaxial growth of CsPbBr_3_/CdS Janus NCs in 2023, confining CdS growth to the {220} facet of CsPbBr_3_ to create highly coherent interfaces (**Figure** **28**).^[^ ^38^ ^]^ This design enabled ultrafast interfacial electron transfer (≈9 ps) and a three‐order‐of‐magnitude increase in photoresponsivity (up to 11.2 A W^−1^) compared to pure CsPbBr_3_, showcasing the potential of precise structural engineering in optimizing carrier dynamics.

Elemental doping also emerges as a pivotal strategy, enabling precise tailoring of band structures and carrier lifetimes to further enhance photocatalytic activity in CsPbBr_3_ heterostructures. By modulating the electronic properties of CsPbBr_3_, dopants such as manganese (Mn)^[^ ^182^ ^]^ and rubidium (Rb)^[^ ^183^ ^]^ extend the lifetime of photogenerated carriers and boost photocatalytic activity, while titanium (Ti)^[^ ^184^ ^]^ and nickel (Ni)^[^ ^185^ ^]^ doping improve charge mobility, reducing electron‐hole recombination and enhancing carrier separation efficiency. Most significantly, the synergy between elemental doping and heterostructure formation creates a dual effect: doping not only adjusts the intrinsic band structure of CsPbBr_3_ but also optimizes band alignment at the heterostructure interface with partner semiconductors, facilitating efficient carrier migration to catalytic active sites.^[^ ^186^, ^187^ ^]^

**Figure 29:**
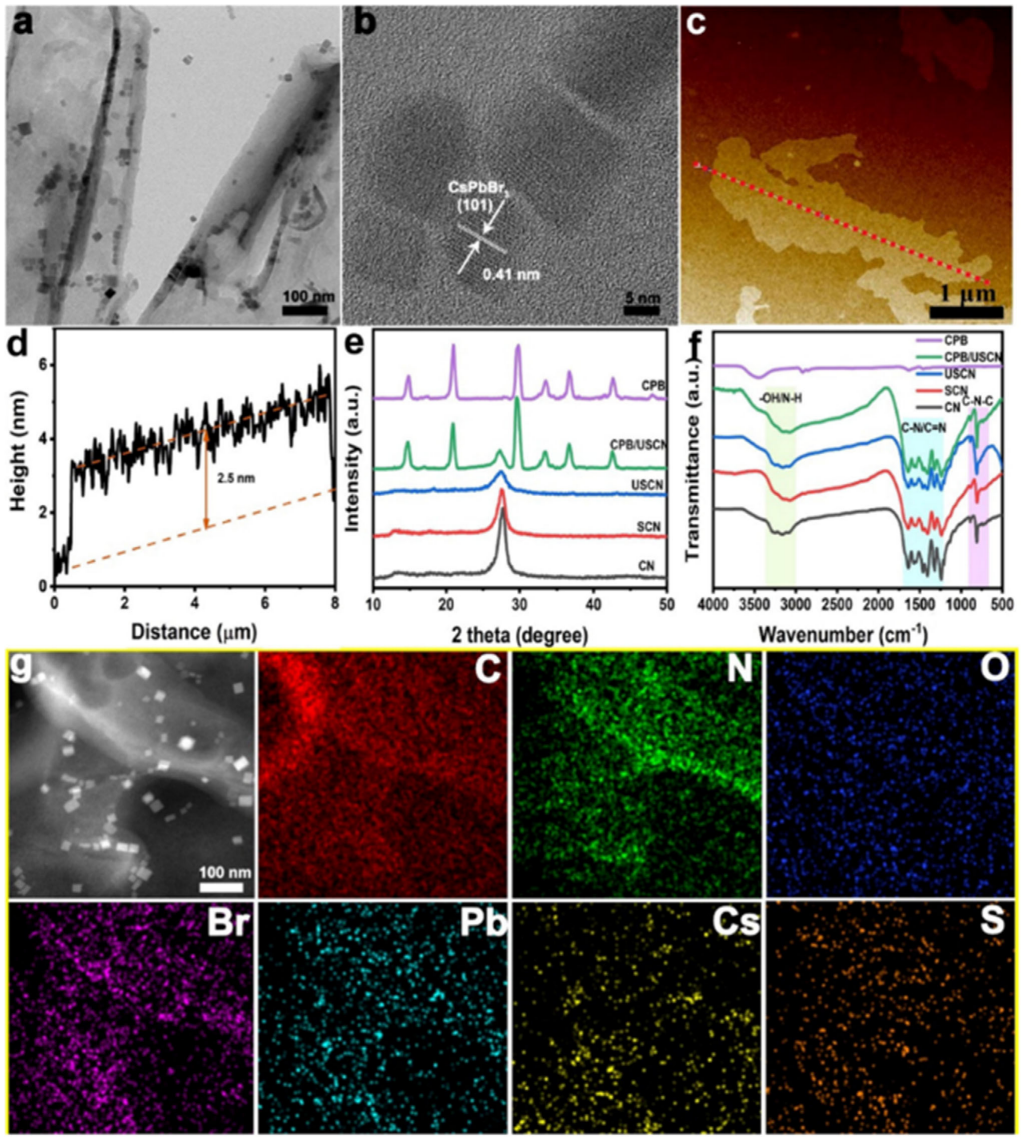
TEM of CPB/USCN a) and HRTEM image of CPB in CPB/USCN b). AFM of S doping g‐C_3_N_4_ ultra‐thin nanosheet c) with the thickness shown as a step height d). XRD patterns e) and FTIR spectra f) of CN, SCN, USCN, CPB/USCN, and CPB. g) Elemental mapping of CPB/USCN. Reproduced with permission.^[^ ^188^ ^]^ Copyright 2022, Elsevier.

Building upon the interface engineering example mentioned above, Chakraborty et al.’s In‐doped CdO/CsPbBr_3_ system exemplifies how elemental doping synergizes with heterostructure formation.^[^ ^181^ ^]^ In this system, In^3+^ doping into the CdO matrix generates multiple beneficial effects: the incorporation of In^3+^ ions enhances the electrical conductivity of CdO, facilitating more efficient charge transfer pathways between CsPbBr₃ and the partner semiconductor. Additionally, the In‐doped CdO exhibits strong LSPR at 0.43 eV in the mid‐infrared region, which improves both light absorption and carrier generation efficiency. These synergistic doping effects resulted in a remarkable 24% enhancement in PLQY compared to undoped counterparts. Notably, this work demonstrates the dual functionality often achieved in well‐designed systems, where the CdO matrix simultaneously provides protective encapsulation for stability enhancement. Zhao et al. advanced this concept in 2022 by integrating sulfur‐doped graphitic carbon nitride (S‐g‐C_3_N_4_) with CsPbBr_3_ to form S‐g‐C_3_N_4_/CsPbBr_3_ heterostructures (**Figure** **29**).^[^ ^188^ ^]^ Sulfur doping introduced impurity levels in the heterostructure, causing a red shift in the light absorption edge and expanding the visible‐light response. The spin‐polarization effect of sulfur reduced carrier recombination rates, increasing the utilization efficiency of photogenerated charges. Under synergistic doping and heterostructure engineering, the photocatalyst achieved a CO_2_ reduction rate of 83.6 µmol g^−1^ h^−1^, which is 16 times higher than single‐component materials, demonstrating the amplified effect of combined strategies on catalytic performance. Wang et al. further explored Ni‐doped CsPbBr_3_/Bi_3_O_4_Br heterostructures via electrostatic assembly in the same year.^[^ ^189^ ^]^ Nickel doping broadened the spectral response of the CsPbBr_3_/Bi_3_O_4_Br heterostructure, enhancing visible‐light absorption and creating new energy levels that optimized electron transport pathways. The Ni sites acted as electron‐rich active centers, facilitating CO_2_ adsorption and activation while lowering the formation energy of key intermediates, thus accelerating reaction kinetics. The resultant catalyst yielded 387.57 µmol g^−1^ of CO, which is 12.3 times higher than undoped CsPbBr_3_ and 3.7 times higher than pure Bi_3_O_4_Br, and exhibited improved CH_4_ selectivity, marking a significant advance in multi‐product photocatalysis.

#### 4.2.2 Stability Enhancement Strategies

**Figure 30:**
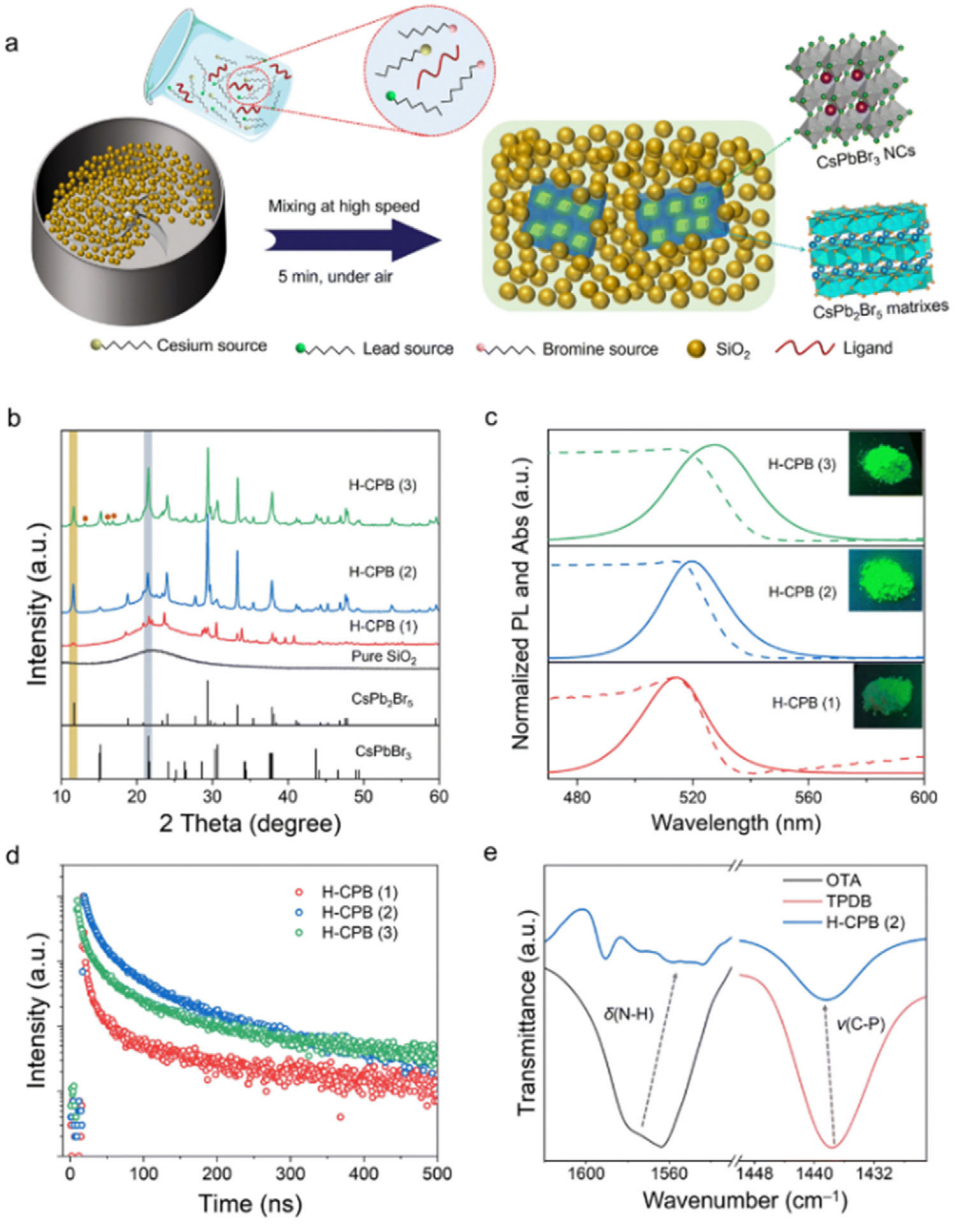
Preparation and performance of H‐CPB composites. a) Schematic illustration of the fabrication process for H‐CPB composites. b) Powder XRD patterns of pure SiO_2_ and H‐CPB composites. c) UV–vis absorption and PL spectra of H‐CPB composites. Inset: Photographs of H‐CPB composites under the irradiation of UV light (365 nm). d) PL decay kinetics for H‐CPB composites. e) FTIR spectra of OTA, TPDB, and the H‐CPB (2). Reproduced with permission.^[^ ^193^ ^]^ Copyright 2024, Royal Society of Chemistry.

Stability enhancement strategies for CsPbBr_3_ heterostructures aim to address the intrinsic degradation vulnerabilities of the material, primarily hydrolysis, ion migration, and lattice collapse, through structural confinement and surface modification. Inorganic encapsulation represents a robust strategy to enhance the stability of CsPbBr_3_ heterostructures. Xu et al. reported a CsPbBr_3_ QD/graphene oxide (GO) composite, where the GO layer facilitated efficient electron extraction and transport.^[^ ^22^ ^]^ Under AM 1.5G simulated sunlight, this composite achieved a CO_2_ reduction rate of 29.8 µmol g^−1^ h^−1^, with a 25.5% increase in electron consumption rate relative to bare CsPbBr_3_ QDs. The material also maintained 78% of its initial PL intensity after immersion in water for 2 h, highlighting the role of GO in suppressing hydrolysis. Su et al. demonstrated another effective inorganic encapsulation approach through in situ coating CsPbBr_3_ nanocrystals with graphdiyne (GDY), achieving remarkable stability and activity improvements.^[^ ^190^ ^]^ The GDY coating provided a robust protective layer that significantly enhanced the structural stability of CsPbBr_3_ in water‐containing photocatalytic systems. The cobalt‐doped CsPbBr_3_@GDY composite exhibited exceptional CO_2_ reduction performance with CO production rates of 27.7 µmol g^−1^ h^−1^, representing an eight‐fold enhancement compared to pristine CsPbBr_3_ (3.3 µmol g^−1^ h^−1^). Hou et al. constructed a CPB QDs/Cu‐BTC heterostructure via in situ growth, where the Cu‐BTC shell served as a protective barrier against moisture‐induced degradation.^[^ ^191^ ^]^ The composite retained 90% of its photoluminescence intensity after 6 h of light irradiation, and its CO production rate reached 47.82 µmol g^−1^ h^−1^, demonstrating a 2.2‐fold enhancement compared to pristine CsPbBr_3_ QDs. Zhu et al. developed a Z‐scheme CsPbBr_3_/oxygen‐doped g‐C_3_N_4_ (OCN) heterostructure, where OCN's porous structure provided both physical confinement and electronic coupling.^[^ ^192^ ^]^ The composite exhibited negligible yield decay after five consecutive reaction cycles, with its XRD pattern remaining unchanged post‐catalysis. This structure enabled the selective sulfoxidation of alkenes with yields up to 89%, showcasing the synergistic effect of inorganic encapsulation on stability and catalytic activity. Yao et al. demonstrated this approach by embedding CsPbBr_3_ into a CsPb_2_Br_5_ matrix using SiO_2_ as a reaction medium, yielding highly stable CsPb_2_Br_5_/CsPbBr_3_ heterostructures (**Figure** **30**a).^[^ ^193^ ^]^ The SiO_2_ nanospheres regulated the nucleation and growth of CsPb_2_Br_5_/CsPbBr_3_, preventing particle agglomeration and ensuring structural uniformity. By tuning the SiO_2_ loading (Cs: Pb: Br: SiO_2_ = 3: 5: 15: 70), the photoluminescence properties were optimized, achieving a PLQY of 83%, which is significantly higher than the 22% observed without SiO_2_. These results highlight the role of inorganic encapsulation in preventing CsPbBr_3_ from contacting harmful substances and maintaining its structural integrity.

**Figure 31:**
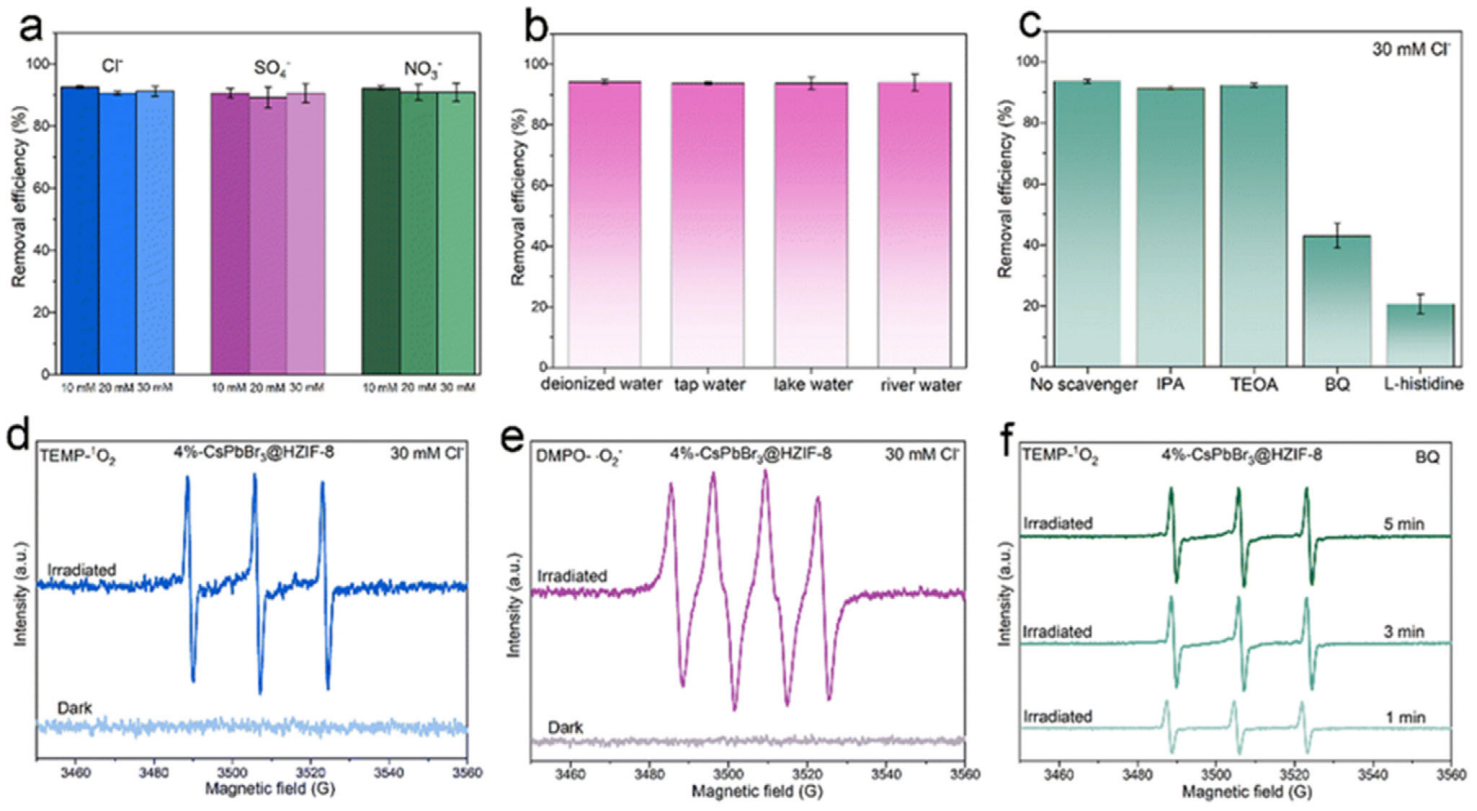
a) The effect of different water matrix on 4%‐CsPbBr3@HZIF‐8 and the degradation of TCH in: Cl^−^, SO^4−,^ and NO^3−^, different water bodies. b) Impacts of water sources on TCH photo‐degradation performance by 4%‐CsPbBr_3_@HZIF‐8. c) The influence of different scavengers on TCH degradation. EPR signals of 4%‐CsPbBr_3_@HZIF‐8 in the presence of DMPO and TEMP for d) ˙O^2−^ and e) ^1^O_2_, respectively. f) EPR spectra for the detection of ^1^O_2_ in the presence of TEMP and BQ. Reproduced with permission.^[^ ^198^ ^]^ Copyright 2023, Royal Society of Chemistry.

Notably, porous structure engineering, such as embedding CsPbBr_3_ within covalent organic frameworks (COFs) or metal‐organic frameworks (MOFs), enhances stability by creating physical barriers against solvent intrusion and ion migration.^[^ ^194^, ^195^, ^196^ ^]^ Kour et al. advanced porous heterostructure via a modified mechanochemical method, synthesizing CsPbBr_3_/Cs_4_PbBr_6_@COF nanocomposites under ambient pressure.^[^ ^197^ ^]^ The COF layer functioned as a protective shell that significantly suppressed water‐induced decomposition of both CsPbBr_3_ and Cs_4_PbBr_6_. When immersed in deionized water for 60 min, the COF‐encapsulated composite maintained its original crystal structure, while the uncoated material underwent complete degradation into CsBr and PbBr_2_. The porous COF matrix increased specific surface area, exposing more active sites and enabling complete degradation of a 10 mL, 100 ppm methyl orange (MO) solution within 25 min, achieving a record‐high degradation rate constant of 0.245 min^−1^ for perovskite‐based photocatalysts. The π‐conjugated electron channels in COF facilitated efficient electron transfer from CsPbBr_3_, reducing carrier recombination and enhancing photocatalytic efficiency. Hou et al. integrated CsPbBr_3_ with the MOF HZIF‐8 via sequential deposition, forming CsPbBr_3_@HZIF‐8 heterostructures with superior resistance to ionic interference.^[^ ^198^ ^]^ The 4%‐CsPbBr_3_@HZIF‐8 composite achieved 94% degradation of tetracycline hydrochloride (TCH) within 40 min, driven by reactive oxygen species (ROS) such as singlet oxygen (^1^O_2_) generated via efficient carrier separation (**Figure** **31**). Notably, the catalyst demonstrated sustained high activity in high‐salinity environments containing Cl^‐^, SO_4_^2−^, NO_3_^−^ ions, as well as in real water matrices including tap, river, and lake water, achieving a degradation efficiency of 93.3%. These studies show that porous engineering with COFs/MOFs stabilizes CsPbBr_3_ heterostructures against solvent/ion attack while boosting photocatalysis via active site exposure and electron transfer, promising for environmental and energy applications. However, despite the advantages offered by these structures, several challenges persist in practical application. First, the increased surface exposure makes the material more susceptible to environmental factors such as moisture and oxygen, which can lead to performance degradation.^[^ ^199^ ^]^ Second, precise control over porosity remains a technical challenge, with difficulties in achieving uniform porosity distribution and structural stability under varying operational conditions.^[^ ^200^ ^]^ Therefore, further optimization of porous structure design strategies is necessary to achieve a balance between performance and durability in practical applications.

## 5 Photocatalytic Applications

Photocatalysis plays a critical role in solar‐to‐chemical energy conversion and pollutant degradation, addressing global energy and environmental challenges.^[^ ^201^ ^]^ This section provides an overview of the development status of CsPbBr_3_ heterostructures in photocatalytic degradation of organic pollutants,^[^ ^202^ ^]^ water splitting for hydrogen production,^[^ ^203^ ^]^ CO_2_ reduction,^[^ ^204^ ^]^ and photocatalytic organic synthesis.^[^ ^73^ ^]^

### 5.1 Organic Pollutant Degradation

The rapid industrialization and urbanization have led to widespread contamination of water and soil by persistent organic pollutants, including dyes (eosin B,^[^ ^205^ ^]^ rhodamine B (RhB),^[^ ^53^ ^]^ methyl orange (MO),^[^ ^206^ ^]^ sudan red III (SRIII)),^[^ ^207^ ^]^ antibiotics (tetracycline (TCH),^[^ ^208^ ^]^ norfloxacin^[^ ^209^ ^]^), and phenolic compounds.^[^ ^210^ ^]^ The photocatalytic degradation of these pollutants relies fundamentally on the generation of reactive oxygen species (ROS), which necessitates precise thermodynamic alignment of the electronic structure of the photocatalyst.^[^ ^211^ ^]^ For effective pollutant degradation, the CBM must be positioned more negative than the O^2^/·O^2‐^ reduction potential (−0.33 V vs NHE) to enable superoxide radical formation, while the VBM should exceed +1.23 V versus NHE for hydroxyl radical (·OH) generation through water oxidation.^[^ ^41^, ^212^ ^]^ CsPbBr_3_ demonstrates excellent thermodynamic suitability for superoxide generation, with its CBM positioned at −1.3 V versus NHE, providing substantial driving force for ·O^2−^ formation. However, its VBM at +1.0 V versus NHE presents limitations for direct ·OH radical generation and complete mineralization processes.^[^ ^41^ ^]^ This thermodynamic constraint necessitates strategic heterostructure engineering to enhance the oxidizing capability while maintaining the inherent advantages of the strong reducing power of CsPbBr_3_. Consequently, the construction of well‐designed heterostructures emerges as the most effective strategy to overcome these limitations and achieve comprehensive pollutant degradation performance.

For instance, Xue et al. fabricated an Ag/CsPbBr_3_/Bi_2_WO_6_ Z‐scheme photocatalyst via electrostatic assembly in 2023, achieving a 93.9% degradation efficiency for 10 mg L^−1^ RhB within 120 min under a 300 W xenon lamp, which is 4.41 times higher than pure Bi_2_WO_6_.^[^ ^53^ ^]^ This performance was attributed to the Z‐scheme charge transfer mechanism, which preserved high redox potentials for efficient ROS generation. The following year, Ding et al. developed a CsPbBr_3_/TiO_2_ Z‐scheme heterostructure via in situ growth, embedding CsPbBr_3_ in mesoporous TiO_2_ to enhance carrier separation. This design achieved a 94.8% RhB degradation efficiency in just 60 min under an AM 1.5G solar simulator, outperforming many state‐of‐the‐art perovskite‐based catalysts.^[^ ^146^ ^]^

**Figure 32:**
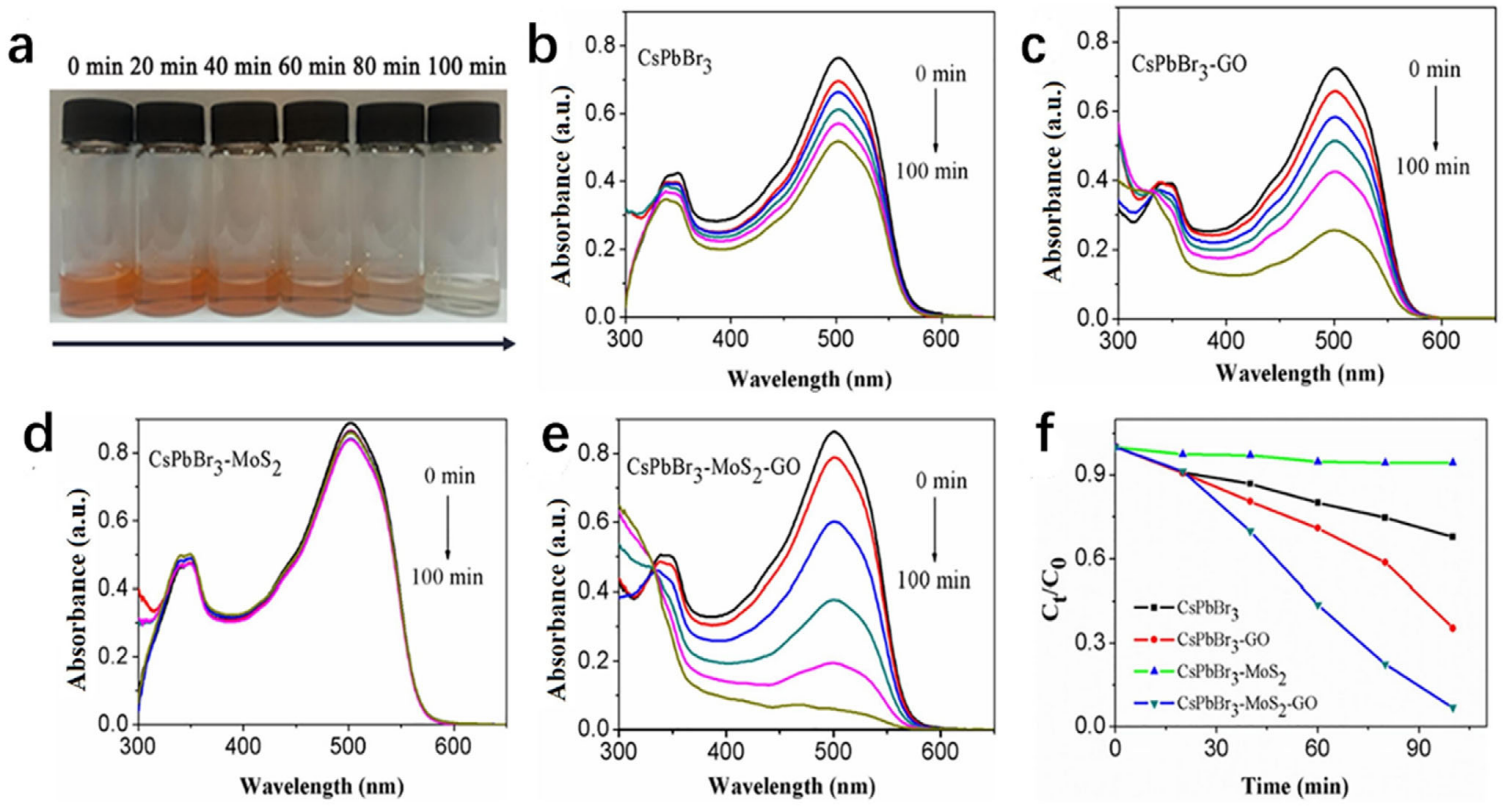
a) Picture of Sudan Red III solution degraded by CsPbBr_3_‐MoS_2_‐GO nanocomposites with different durations time. Absorption spectra of Sudan Red III degraded by. b) CsPbBr_3_ QDs. c) CsPbBr_3_‐GO nanocomposites. d) CsPbBr_3_‐MoS_2_ nanocomposites and e) CsPbBr_3_‐MoS_2_‐GO nanocomposites with illumination time. f) Concentration (C_t_/C_0_) changes of Sudan Red III with different catalysts under visible light irradiation. Reproduced with permission.^[^ ^213^ ^]^ Copyright 2021, Elsevier.

Zhang et al. demonstrated the synergistic effect of porous MOF integration by synthesizing CsPbBr_3_/UiO‐66 Z‐scheme heterostructures through in situ loading. The composite degraded over 90% of 10 mg L^−1^ MO within 90 min under 300 W xenon light, which is significantly higher than the 57.7% efficiency of pure CsPbBr_3_, highlighting the role of UiO‐66 in expanding light absorption and providing additional active sites.^[^ ^165^ ^]^ Ju et al. further expanded the application scope to SRIII degradation using a CsPbBr_3_‐MoS_2_‐GO heterostructure prepared by ultrasonic dispersion (**Figure** **32**). The nanocomposite achieved a 93.1% degradation efficiency for 10 mg L^−1^ SRIII in 100 min, a 3.1‐fold improvement over pure CsPbBr_3_ QDs, demonstrating the benefits of graphene oxide (GO) in enhancing charge transfer.^[^ ^213^ ^]^

Hou et al. addressed real‐world applicability by creating a hierarchical porous CsPbBr_3_@HZIF‐8 heterostructure via an in‐ship bottle sedimentation method, which degraded 94% of TCH in 40 min even under high‐salinity conditions.^[^ ^198^ ^]^ The catalyst retained over 93% efficiency in diverse aqueous environments, including tap, river, and lake water, demonstrating exceptional resistance to ionic interference, a critical requirement for practical wastewater remediation applications. Other notable systems include CsPbBr_3_‐rGO/Bi_2_WO_6_ for norfloxacin degradation,^[^ ^209^ ^]^ CsPbBr_3_/MOF‐808 for ceftriaxone removal,^[^ ^196^ ^]^ and CsPbBr_3_/Bi_2_WO_6_ for polycyclic aromatic hydrocarbons (PAHs) degradation,^[^ ^153^ ^]^ all demonstrating high efficiency under simulated solar light.

These studies collectively highlight the versatility of CsPbBr_3_ heterostructures in degrading diverse organic pollutants, with performance metrics summarized in **Table** **7**. The combination of rational heterostructure design, efficient carrier separation, and robust stability positions CsPbBr_3_‐based materials as promising solutions for environmental remediation, bridging laboratory innovation and real‐world pollution control.

**Table 7:** Summary of photocatalytic performance of different CsPbBr_3_ based photocatalysts for the degradation of organic pollutants.

No.	Photocatalyst	Synthesis method	Light source	Photocatalytic performance	Refs.
1	Ag/CsPbBr_3_/Bi_2_WO_6_	Electrostatic assembly method	300 W xenon lamp	Degradation efficiency of RhB is 93.9% (10 mg L^−1^ RhB, 120 min)	[53]
2	CsPbBr_3_/TiO_2_	In situ synthesis	AM 1.5G solar simulator (100 mW cm^−^^2^)	Degradation efficiency of RhB is 94.8% (10 mg L^−1^ RhB, 60 min)	[146]
3	CsPbBr_3_/UiO‐66	In situ loading method	300 W xenon lamp	Degradation efficiency of MO is 90% (10 mg L^−1^ MO, 90 min)	[165]
4	CsPbBr_3_‐MoS_2_‐GO	Ultrasonic Dispersed Method	300 W xenon lamp	Degradation efficiency of SRIII is 93.1% (10 mg L^−1^ SRIII, 100 min)	[213]
5	CsPbBr_3_@HZIF‐8	In ship bottle sedimentation method	300 W xenon lamp	Degradation efficiency of TCH is 94% (10 mg L^−1^ TCH, 40 min)	[198]
6	CsPbBr_3_‐rGO/Bi_2_WO_6_	Electrostatic assembly method	300 W xenon lamp	Degradation efficiency of Norfloxacin is 66.79% (10 mg L^−1^ Norfloxacin, 120 min)	[209]
7	CsPbBr_3_/MOF‐808	Solvent‐free mechanochemical method	500 W xenon lamp	Degradation efficiency of Ceftriaxone is 97% (10 mg L^−1^ Ceftriaxone, 120 min)	[196]
8	CsPbBr_3_/Bi_2_WO_6_	Ultrasonic Dispersed Method	500 W xenon lamp	Degradation efficiency of PAHs is 96% (10 mg L^−1^ PAHs, 120 min)	[153]

Abbreviations: RhB, rhodamine B; MO, Methyl Orange; SRIII, Sudan Red III; TCH, Tetracycline; PAHs, Polycyclic Aromatic Hydrocarbons.

### 5.2 Water Splitting

Photocatalytic water splitting represents a cornerstone technology for sustainable hydrogen production, offering a direct solar‐to‐fuel conversion pathway to address the growing demand for clean energy alternatives.^[^ ^214^, ^215^, ^216^ ^]^ The thermodynamic requirements for overall water splitting impose stringent constraints on photocatalyst design. For the hydrogen evolution reaction (HER), the CBM must be positioned more negative than the H^+^/H_2_ potential (0 V vs NHE), while the OER necessitates a valence band maximum (VBM) more positive than the O_2_/H_2_O potential (+1.23 V vs NHE).^[^ ^40^, ^41^, ^217^ ^]^ Beyond these fundamental thermodynamic criteria, successful water splitting photocatalysts must demonstrate exceptional aqueous stability to withstand prolonged exposure to water without structural degradation,^[^ ^164^, ^169^ ^]^ alongside efficient charge transport properties to minimize electron‐hole recombination losses and maximize surface redox reactions. CsPbBr_3_ presents a mixed thermodynamic profile for water splitting applications. Its CBM at −1.3 V versus NHE provides substantial thermodynamic driving force for hydrogen evolution, significantly exceeding the minimum requirement and ensuring efficient electron transfer for H_2_ generation.^[^ ^218^ ^]^ However, the material faces a critical limitation with its VBM positioned at +1.0 V versus NHE, which falls short of the +1.23 V threshold required for direct water oxidation to oxygen.^[^ ^219^ ^]^ On the other hand, CsPbBr_3_ undergoes rapid degradation in aqueous environments, primarily due to ion leaching and photocorrosion, which limit its practical application.^[^ ^77^, ^78^ ^]^ This thermodynamic mismatch, combined with the inherent instability of lead halide perovskites in aqueous environments, necessitates innovative heterostructure engineering approaches or the development of alternative oxidation pathways to realize efficient and stable water splitting performance.

**Figure 33:**
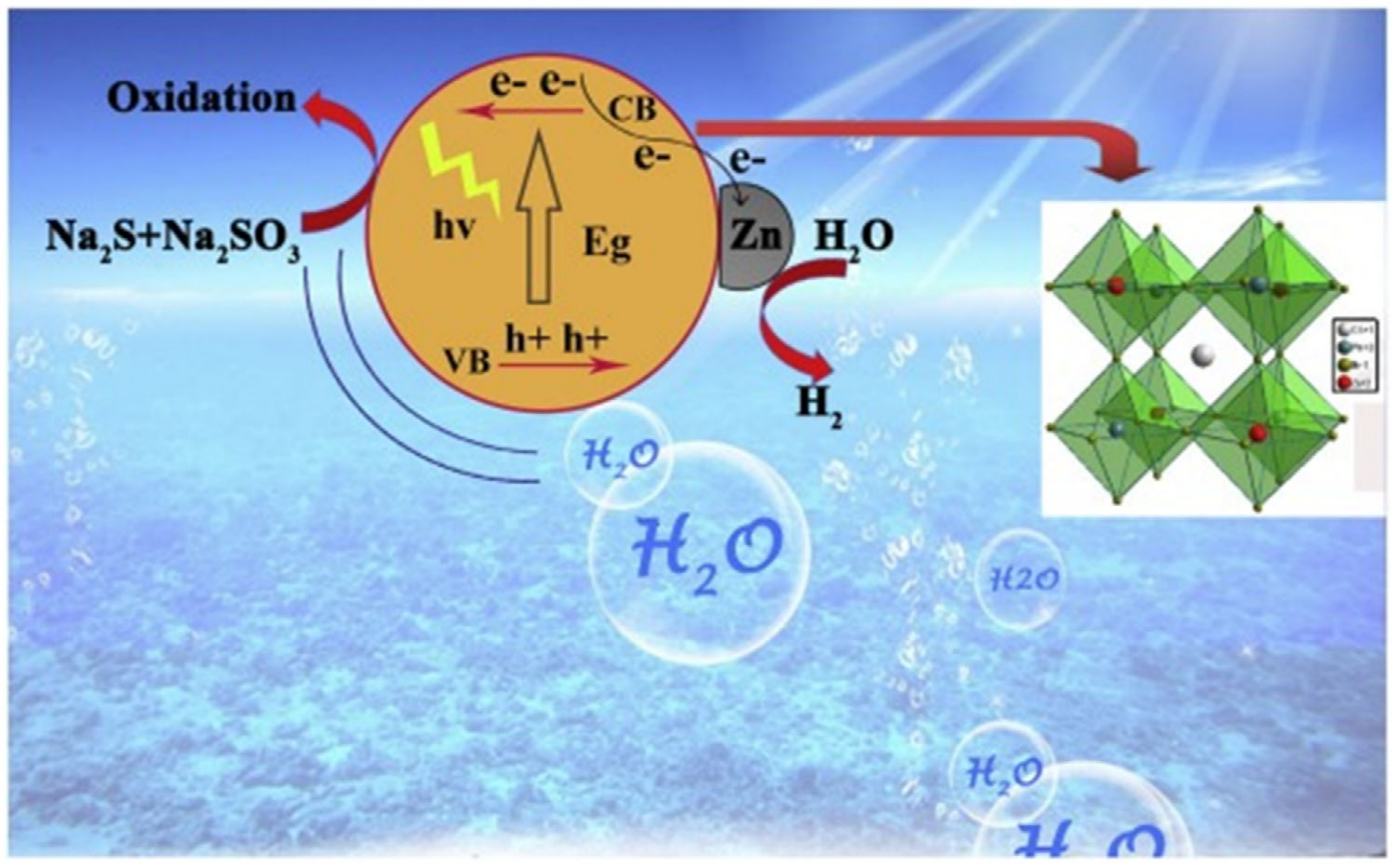
Schematic diagram of photocatalyst mechanism for carrier transfer and hydrogen evolution. Reproduced with permission.^[^ ^220^ ^]^ Copyright 2020, Elsevier.

Ding et al. synthesized Zn^2+^‐doped CsPbBr_3_ nanocrystal glass via a molten‐quenching method in 2020, achieving notable advancements in both hydrogen evolution and material stability.^[^ ^220^ ^]^ The 0.5 mol% Zn^2+^‐doped sample exhibited a hydrogen evolution rate of 127.04 µmol g^−1^ over 10 h, a 64% increase compared to undoped CsPbBr_3_ (77.23 µmol g^−1^. Importantly, Zn doping mitigated the typical activity decay of CsPbBr_3_ in water splitting, as evidenced by sustained performance without significant degradation (**Figure** **33**). This work demonstrated that cation doping enhances photocatalytic efficiency while suppressing material degradation under harsh reaction conditions. Song et al. further improved water stability in 2021 by coating CsPbBr_3_ nanocrystals with the conductive polymer polyaniline (PANI), forming CsPbBr_3_@PANI nanoparticles.^[^ ^221^ ^]^ The optimized CsPbBr_3_@PANI nanoparticles achieved a remarkable hydrogen production rate of 4.81 mmol h^−1^ g^−1^ under xenon lamp irradiation, with a catalytic lifetime exceeding 120 h, significantly surpassing previous reports for unprotected CsPbBr_3_. This work highlighted the potential of conductive polymer coatings in enabling halide perovskites for long‐term photocatalytic applications in aqueous environments. Additionally, Gao et al. demonstrated a breakthrough approach in 2024 by confining CsPbBr_3_ nanocrystals within extra‐large‐pore zeolite ZEO‐1, achieving exceptional hydrogen evolution performance of 1734 µmol h^−^¹ g^−^¹ (CsPbBr_3_), representing a two‐order‐of‐magnitude enhancement over bulk CsPbBr_3_ (11 µmol h^−^¹ g^−^¹).^[^ ^71^ ^]^ The nanoconfinement effect led to band gap narrowing from 2.23 to 2.19 eV, enabling enhanced visible light utilization, while the zeolite matrix provided exceptional stability in acidic HI solution for 36 h. This pioneering work demonstrates the great potential of inorganically encapsulated, confined perovskite nanocrystals for robust and efficient photocatalytic water splitting applications.

Despite these advancements, CsPbBr_3_‐based photocatalysts for water splitting still face challenges, particularly degradation in aqueous environments and the need for further efficiency improvements to enable large‐scale hydrogen production. Current heterostructure designs focus on surface passivation and interface engineering to suppress dissolution, but the potential of novel material combinations, such as 2D materials^[^ ^222^ ^]^ and metal‐organic frameworks,^[^ ^223^, ^224^ ^]^ and structural configurations remains underexplored. Future research should prioritize rational design of heterostructures with optimized band alignment and robust protective layers, aiming to balance catalytic activity, stability, and scalability for practical energy conversion applications.

### 5.3 CO_2_ Reduction

As a pivotal strategy for achieving carbon neutrality, photocatalytic CO_2_ reduction has garnered significant attention for its ability to convert CO_2_ into high‐value chemical fuels (e.g., CO, CH_4_, CH_3_OH), simultaneously addressing environmental pollution and energy shortages by reducing greenhouse gas emissions and producing renewable energy carriers.^[^ ^225^, ^226^, ^227^, ^228^, ^229^ ^]^ The photocatalytic conversion of CO_2_ presents significant thermodynamic and kinetic challenges due to the inherent stability of the CO_2_ molecule and the complexity of multi‐electron transfer processes.^[^ ^230^, ^231^ ^]^ Unlike single‐electron reactions, CO_2_ reduction typically involves multiple electron‐proton coupled steps, with reduction potentials ranging from −0.52 V versus NHE for CO_2_‐to‐CO conversion to ‐0.24 V versus NHE for CO_2_‐to‐CH_4_ formation.^[^ ^230^ ^]^ Moreover, the inherent complexity of CO_2_ reduction stems from the need for both sufficient thermodynamic driving force and effective catalytic sites to facilitate the multi‐electron transfer processes.^[^ ^232^ ^]^ These demanding reduction potentials necessitate photocatalysts with highly negative conduction band positions to provide sufficient thermodynamic driving force. CsPbBr_3_ demonstrates exceptional promise for CO_2_ photoreduction applications due to its highly negative conduction band minimum at −1.3 V versus NHE, which provides substantial thermodynamic driving force for various CO_2_ reduction pathways.^[^ ^22^, ^164^ ^]^ This favorable band alignment, combined with its strong light absorption and efficient charge generation properties, positions CsPbBr_3_ as an ideal platform for CO_2_ conversion when integrated with appropriate co‐catalysts or heterostructure partners to address surface activation and selectivity challenges.^[^ ^22^, ^233^, ^234^, ^235^ ^]^

**Figure 34:**
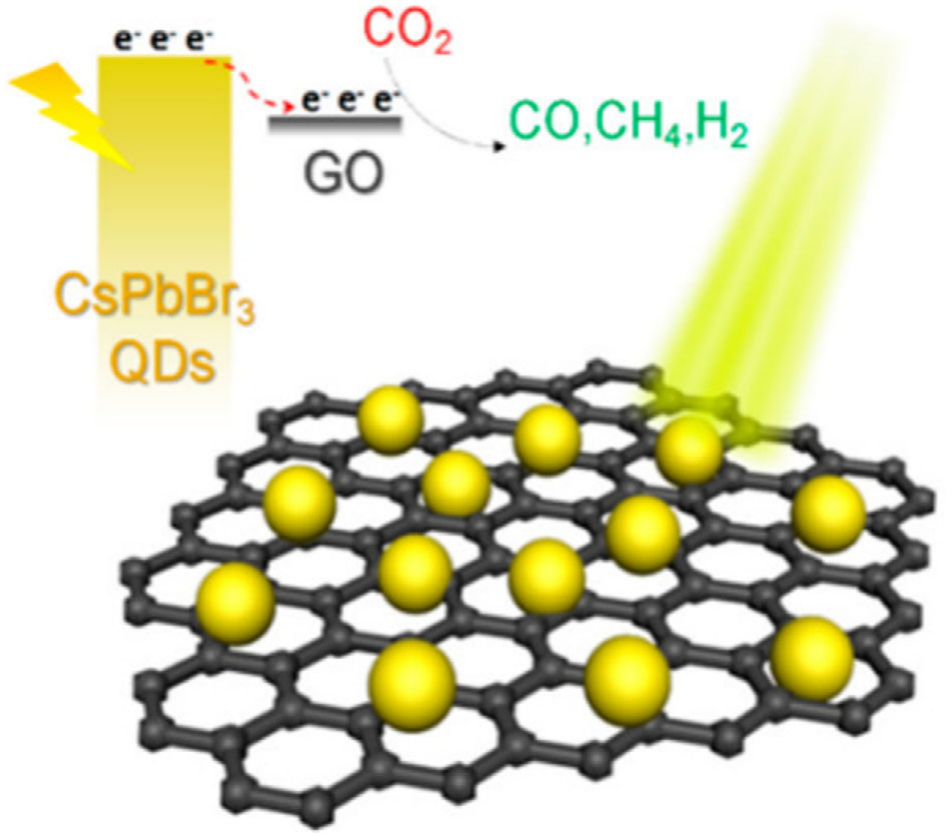
Schematic diagram of CO_2_ photoreduction over the CsPbBr_3_ QD/GO photocatalyst. Reproduced with permission.^[^ ^22^ ^]^ Copyright 2017, American Chemical Society.

The application of CsPbBr_3_‐based materials in photocatalytic CO_2_ reduction was first reported by Xun et al. in 2017 through the development of CsPbBr_3_ QDs/graphene oxide (GO) heterostructures via a facile physical adsorption strategy.^[^ ^22^ ^]^ The composite achieved a CO_2_ reduction rate of 29.8 µmol g^−1^ h^−1^, significantly outperforming pure CsPbBr_3_, owing to enhanced electron consumption facilitated by GO, as shown in **Figure** **34**. Under AM 1.5G solar illumination, CsPbBr_3_ QDs generated electron‐hole pairs, with electrons rapidly migrating to the GO surface via the heterostructure interface. As a highly conductive material with a Fermi level below the CB of CsPbBr_3_, GO not only accelerated charge separation but also enhanced CO_2_ adsorption through its abundant surface functional groups. The reaction mechanism involves the following steps:

1. Photogeneration of carriers in CsPbBr_3_: $\mathrm{CsPbB}r_{3}\mathrm{QDs}+h\nu \to e^{-}\left(\mathrm{CB}\right)+h^{+}\left(\mathrm{VB}\right),$
2. Electron transfer from CsPbBr_3_ to GO: $e^{-}\left({\mathrm{CsPbBr}}_{3}\mathrm{CB}\right)\to e^{-}\left(\mathrm{GO}\right)$
3. CO_2_ adsorption and activation on GO: $CO_{2}+e^{-}\to CO_{2}^{-}$
4. Reduction of CO_2_ to products:
5. 2‐electron pathway for CO formation: $CO_{2}+2H^{+}+2e^{-}\to CO+H_{2}O$
6. 8‐electron pathway for CH_4_ formation: $CO_{2}+8H^{+}+8e^{-}\to CH_{4}+2H_{2}O$

**Figure 35:**
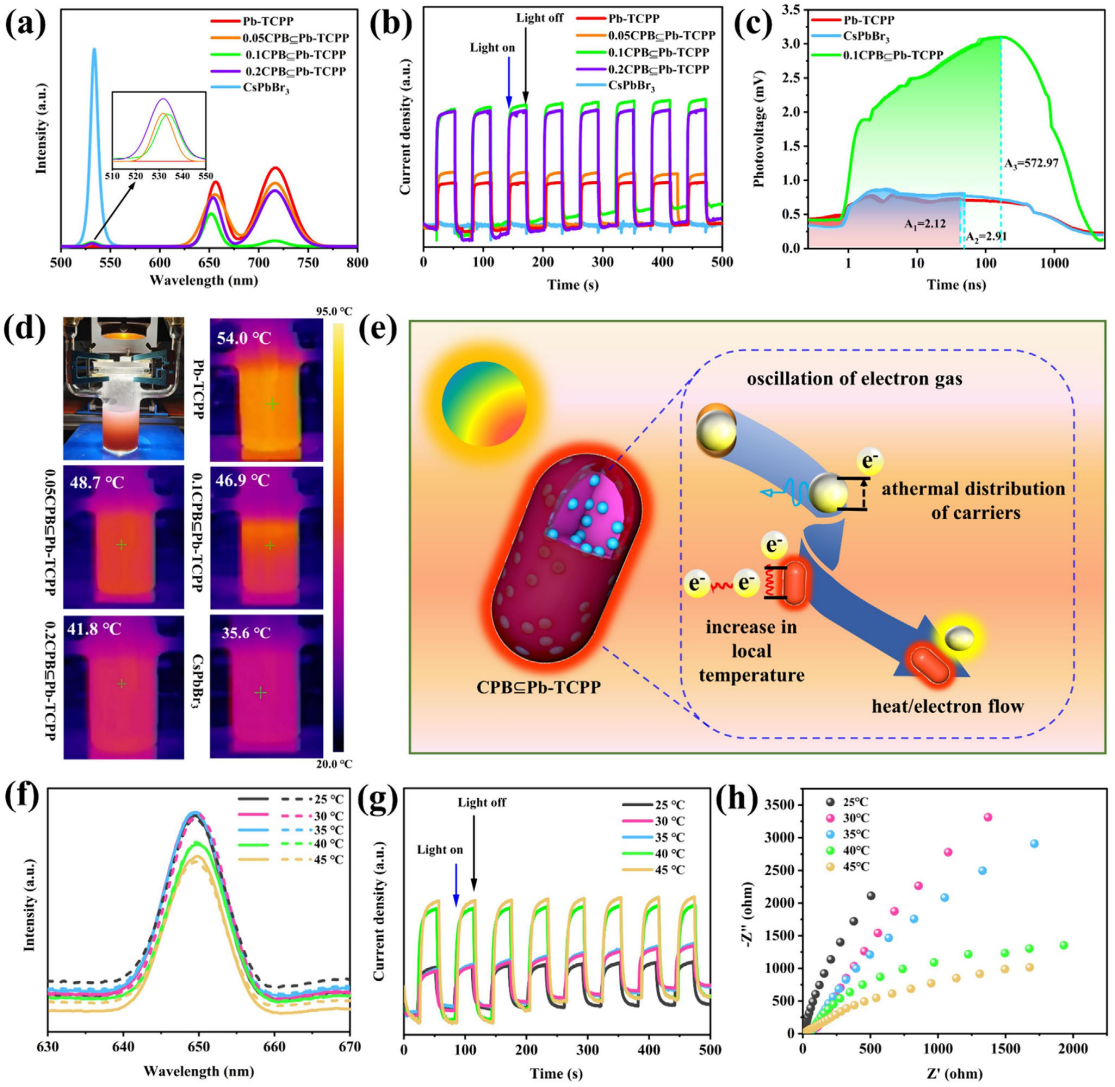
With or without exogenous heating: a,f) PL spectra and b,g) photocurrent density curves; c) TPV curves, d) Photothermal IR images during the reaction process, e) The proposed scheme photoinduced heat and electron flow effect, h) EIS plots over 0.1CPB ⊆ Pb‐TCPP under exogenous heating. Reproduced with permission.^[^ ^235^ ^]^ Copyright 2024, Elsevier.

Subsequent studies have further advanced CsPbBr_3_‐based heterostructures for CO_2_ reduction. Jiang et al. developed a 2D/2D CsPbBr_3_/Bi_2_WO_6_ Z‐scheme heterostructure, which achieved an electron consumption rate of 324.0 µmol g^−1^ h^−1^ and a CO selectivity of 95.5% due to the synergistic effect between the narrow band gap of Bi_2_WO_6_ and the strong light absorption of CsPbBr_3_.^[^ ^233^ ^]^ Wang et al. constructed a PCN‐222/CsPbBr_3_ heterostructure, leveraging the built‐in electric field of the MOF to direct electron transfer, resulting in a formic acid (HCOOH) production rate of 189.9 µmol g^−1^ h^−1^ with 100% selectivity.^[^ ^234^ ^]^ In 2024, Luo et al. integrated CsPbBr_3_ nanocrystals into a Pb‐TCPP MOF to form an S‐scheme heterostructure, achieving enhanced CO (143.3 µmol g^−1^ h^−1^) and CH_4_ (18.6 µmol g^−1^ h^−1^) yields through photothermal synergy (local temperature elevation to 45 °C), as illustrated in **Figure** **35**, which promoted C═O bond polarization and carrier migration.^[^ ^235^ ^]^

Current research on all‐inorganic CsPbBr_3_ perovskite‐based heterostructures for photocatalytic CO_2_ reduction has identified two primary optimization strategies. The first is charge dynamics regulation, where band alignment engineering in heterostructures (e.g., S‐scheme or Z‐scheme) creates built‐in electric fields to drive the directional separation of photogenerated carriers. For example, the TiO_2_‐(101)/CsPbBr_3_ heterostructure shows strong band bending due to Fermi level differences, resulting in a 15.6‐fold increase in CO production compared to pure TiO_2_.^[^ ^236^ ^]^ The second strategy is enhancing CO_2_ adsorption and activation. Porous materials like MOFs and GO increase CO_2_ adsorption through their high specific surface areas and surface functional groups. The confinement effect of PCN‐222, for instance, promotes the preferential growth of CsPbBr_3_ QDs within its pores, optimizing electron transfer pathways.^[^ ^234^ ^]^ As summarized in **Table** **8**, these approaches have enabled diverse product yields across different heterostructures, including CO (19.49–192.3 µmol g^−1^ h^−1^), CH_4_(16.0–86.0 µmol g^−1^ h^−1^), and HCOOH (34.2–189.9 µmol g^−1^ h^−1^), showcasing the effectiveness of heterostructure design in boosting CO_2_ reduction performance. However, despite these advancements, significant challenges remain. CsPbBr_3_ suffers from poor stability in humid environments, and there is limited selectivity toward multi ‐carbon products. Future research should focus on stability enhancement strategies such as MOF encapsulation and polymer passivation, as well as the precise control of multi‐electron reactions (e.g., dual‐copper site designs for C−C coupling). Moreover, exploring the synergistic effects between photothermal and plasmonic properties could enable the selective conversion of CO_2_ into high‐value hydrocarbons, bringing CsPbBr_3_‐ based catalysts closer to practical applications.

**Table 8:** Summary of CO_2_ Reduction Performance of Different CsPbBr_3_‐Based Photocatalysts.

No.	Photocatalyst	Synthesis method	Light source	Products and yield [µmol g^−1^ h^−1^]	Refs.
1	CsPbBr_3_/GO	Physical adsorption method	100 W xenon lamp	CO (58.7) CH_4_ (29.6) H_2_ (1.58)	[22]
2	CsPbBr_3_/Bi_2_WO_6_	Electrostatic self‐assembly	150 W xenon lamp	CO (56.3) CH_4_ (86.0) H_2_ (10.9)	[233]
3	PCN‐222/CsPbBr_3_	Electrostatic self‐assembly	300 W xenon lamp	HCOOH (189.9)	[234]
4	CsPbBr_3_⊆Pb‐TCPP	Sacrificial Conversion Method	300 W xenon lamp	CO (143.3) CH_4_ (18.6)	[235]
5	CsPbBr_3_@Ag‐CN	Photoreduction method	300 W xenon lamp	CO (19.49)	[237]
6	CsPbBr_3_/Ti_3_C_2_Tx	Freeze drying self‐assembly method	300 W xenon lamp	CO (78.4) HCOOH (34.2)	[238]
7	CsPbBr_3_@PANI	Sequential Deposition Method	300 W xenon lamp	CO (26.1)	[239]
8	CsPbBr_3_@SnO_2_	In situ synthesis	300 W xenon lamp	CO (192.3) CH_4_ (16.0)	[195]

### 5.4 Photocatalytic Organic Synthesis

Photocatalytic organic synthesis represents a transformative approach that enables bond formations through selective electron and energy transfer processes under remarkably mild conditions, which preserves sensitive organic substrates and allows for late‐stage functionalization of complex molecules with excellent functional‐group tolerance.^[^ ^240^, ^241^ ^]^ The fundamental requirements for effective photocatalytic organic transformations center on achieving precise control over reaction selectivity and the ability to activate specific functional groups without compromising other structural elements. Critical material properties include the capacity for selective activation of particular C─H, C─C, or C═C bonds while maintaining the integrity of other functional groups, which demands carefully tuned redox potentials that can facilitate targeted electron transfer processes. The photocatalyst must demonstrate controlled oxidation state management to achieve selective transformation levels while operating under ambient conditions to preserve sensitive organic substrates, with chemical inertness toward organic solvents and reaction intermediates ensuring catalyst stability and suppression of competing side reactions. CsPbBr_3_ demonstrates exceptional suitability for photocatalytic organic synthesis through its optimal band positions that provide sufficient driving force for diverse transformations.^[^ ^242^ ^]^ The material exhibits ultrafast charge transfer kinetics with recombination rates much slower than substrate transfer, ensuring efficient radical generation.^[^ ^73^, ^243^ ^]^ Its remarkable air tolerance, with negligible oxygen quenching effects, enables high‐yield reactions under ambient conditions.^[^ ^73^ ^]^ Furthermore, the dynamic halide exchange capability allows bandgap tuning while surface ligand engineering expands substrate scope and enhances selectivity control.^[^ ^108^, ^244^ ^]^

Recent advances in perovskite heterostructure photocatalysis have revolutionized organic synthesis by leveraging unique carrier dynamics to control reaction pathways. In 2018, Schünemann et al. pioneered the use of CsPbBr_3_/TiO_2_ heterostructures for the oxidation of benzyl alcohol under visible light, achieving over 99% selectivity for benzaldehyde with a 50% formation rate.^[^ ^245^ ^]^ At the same time, the production rate of benzaldehyde reached 50%, which was twice the reaction rate of pure TiO_2_, with minimal benzoic acid byproducts. This work highlighted the potential of perovskites for highly selective transformations. Building on this foundation, Zhao et al. developed an innovative anion‐exchange strategy to fabricate asymmetric CsPbBr_x_Cl_3‐x_/TiO_2_ heterostructures, achieving remarkable C(sp3)‐H bond activation in toluene oxidation with a benzaldehyde production rate of 1874 µmolg^−1^ h^−1^ (≈4 times that of naked CsPbBr_3_ nanocrystals), demonstrating how asymmetric halide distribution creates funnel‐like band structures that enhance interfacial charge transfer.^[^ ^244^ ^]^ Subsequently, Dong et al. employed electrostatic assembly to construct CsPbBr_3_/Ni_4_P_2_ heterostructures, achieving 100% benzaldehyde selectivity and 53.2% conversion with negligible byproducts, demonstrating how interfacial engineering can synergistically enhance both reaction efficiency and selectivity.^[^ ^246^ ^]^

**Figure 36:**
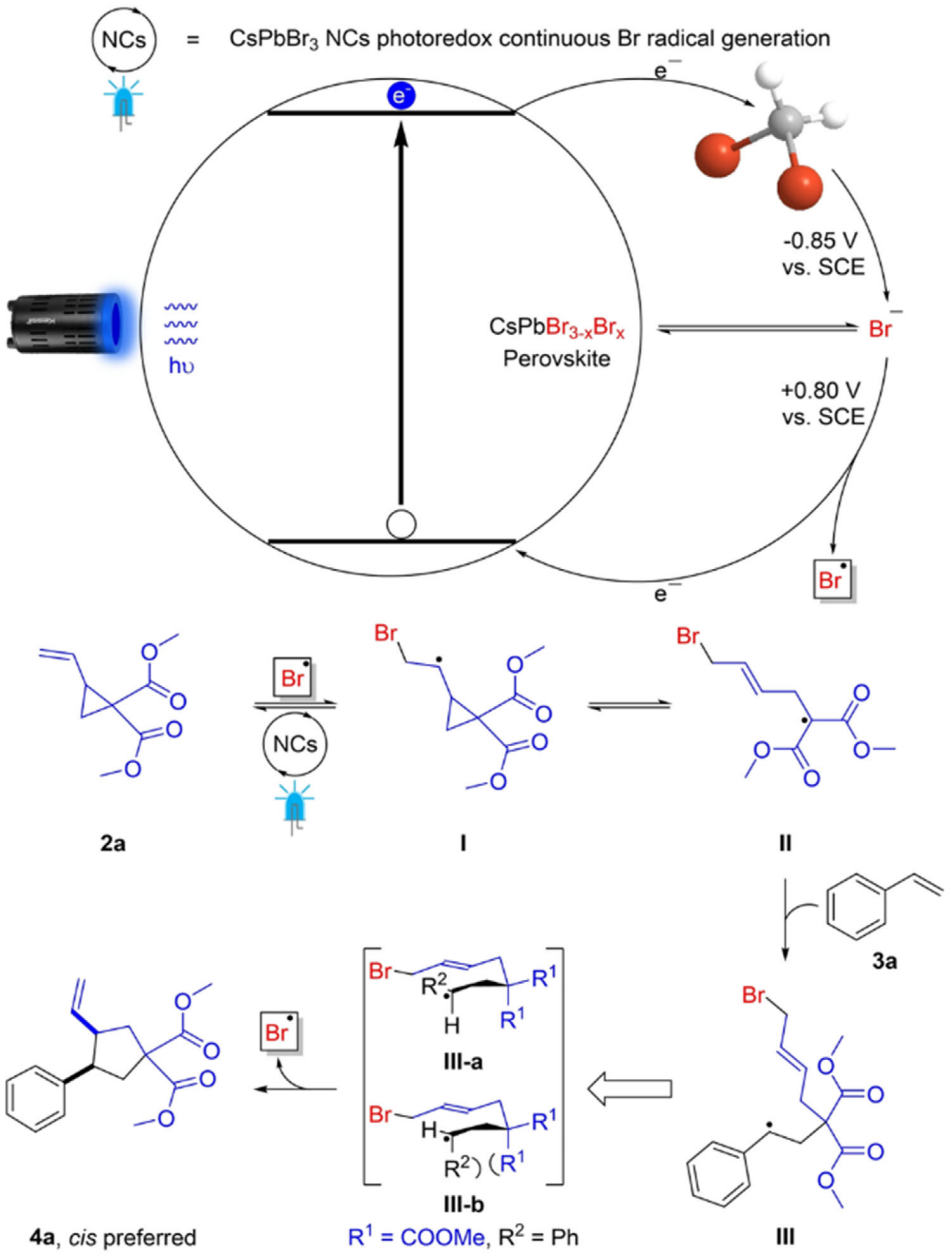
Proposed mechanism for NCs‐photocatalyzed Br‐mediated [3+2] cycloaddition. Reproduced with permission.^[^ ^243^ ^]^ Copyright 2023, Wiley‐VCH.

Notably, the scope of CsPbBr_3_‐based photocatalysts has expanded to complex organic conversions. Ling et al. demonstrated visible‐light‐induced bromine radical generation by CsPbBr_3_ nanocrystals in dibromomethane, driving [3+2] cycloaddition reactions to synthesize pharmaceutically relevant vinyl cyclopentane with 94% yield and a 2:1 trans/cis isomer ratio (**Figure** **36**), highlighting their unique capability for stereoselective synthesis.^[^ ^243^ ^]^ Fan et al. further demonstrated the versatility of CsPbBr_3_ by establishing a visible‐light‐driven C═C bond cleavage system under oxygen, efficiently converting diverse olefins such as electron‐donor/acceptor‐substituted aromatic olefins and α‐methyl olefins into corresponding carbonyl compounds with exceptional substrate generality.^[^ ^247^ ^]^ These studies collectively establish a multi‐dimensional reaction framework spanning simple oxidations to complex bond rearrangements.

In conclusion, CsPbBr_3_‐based photocatalysts show remarkable versatility across reaction types, with composites achieving > 99% selectivity in redox reactions and pristine CsPbBr_3_ delivering excellent yields (>94%) in addition and pyrolysis reactions (**Table** **9**). Despite these breakthroughs, key challenges such as an incomplete understanding of key activation mechanisms and persistent selectivity issues hinder rational catalyst design. The design of stereoselective addition systems remains in its early stages, and the mechanisms of functional group activation are still unclear. Future research must address these gaps through synergistic catalyst engineering and mechanistic investigation. Strategies such as gradient heterostructure design for optimizing charge separation, surface ligand modification for tailoring molecular adsorption, and in situ characterization for decoding dynamic interfacial processes are crucial for establishing structure‐activity relationships and overcoming bottlenecks.

**Table 9:** Summary of Organic Synthesis Performance of Different CsPbBr_3_ Based Photocatalysts.

Reaction type	Photocatalyst	Synthesis method	Light source	Photocatalytic performance	Refs.
Redox reaction	CsPbBr_3_/TiO_2_	Wet‐impregnation method	300 W xenon lamp	The conversion rate of BzOH is 50% and the selectivity is 99% (240 min)	[245]
	CsPbB_3_/Ni_4_P_2_	Electrostatic assembly method	300 W xenon lamp	The conversion rate of BzOH is 53.2% the selectivity of BzH is100% (240 min)	[246]
Addition reaction	CsPbBr_3_	Hot injection method	40 W Light emitting diode	Vinyl cyclopentane generation rate is 94%	[243]
Pyrolysis reaction	CsPbBr_3_	In situ bottle sedimentation method	40 W Light emitting diode	The total production of aldehydes and ketones is 95%	[247]

Abbreviations: BzOH, Benzyl Alcohol; BzH, Benzaldehyde,

## 6 Conclusion

This review comprehensively explores CsPbBr_3_‐based heterostructures in photocatalysis, integrating crystal structure analysis, synthesis methodologies, heterostructure classification, and multifunctional applications. The orthorhombic stability and band alignment dynamics of CsPbBr_3_ are dissected to unravel how Type‐II, Z‐scheme, and S‐scheme architectures address intrinsic limitations such as charge recombination and aqueous degradation. Mechanistic insights into interfacial charge transfer, redox potential retention, and defect tolerance engineering are detailed, alongside practical strategies for activity modulation and stability enhancement. Through this integrated analysis, key synergies between structural design and photocatalytic performance are highlighted, yet several critical challenges persist that hinder widespread implementation.

Despite these breakthroughs, key challenges still remain: 1) Long‐term operational stability remains insufficient for industrial requirements, as ion migration, lattice degradation, and environmental stressors continue to compromise material integrity under realistic operating conditions including humidity fluctuations, prolonged illumination, and temperature cycling; 2) Atomic‐level understanding of heterogeneous interface charge transfer mechanisms, particularly the role of specific point defects including bromide vacancies, lead interstitials, and antisite defects at the CsPbBr_3_/paired‐semiconductor interface, hinders the rational design of efficient heterostructures. These defects act as charge carrier traps and recombination centers, creating energy barriers that impede efficient electron‐hole separation and transport across the heterostructure interface, thereby limiting photocatalytic performance; 3) Scalable synthesis of heterostructures with uniform interfaces, controlled nanostructures, and reproducible performance metrics remains a significant technological bottleneck limiting commercial viability.

To address these challenges, future research needs to further deepen the fundamental understanding of the dynamic structural evolution of CsPbBr_3_ heterostructures under in situ operating conditions, as well as the defect‐mediated charge transfer processes. Advanced characterization techniques, such as in situ grazing‐incidence XRD and time‐resolved cathodoluminescence, are crucial for elucidating real‐time phase transitions, defect migration pathways, and interfacial charge dynamics during photocatalytic cycling. Concurrently, theoretical studies should integrate density functional theory (DFT) with machine learning (ML) to establish predictive frameworks that correlate specific structural descriptors, including bromide vacancy dynamics, lead‐bromide octahedral distortion modes, and interfacial lattice strain parameters, with carrier recombination losses and photocatalytic efficiency metrics. This approach operates through a systematic workflow where DFT calculations generate electronic structure datasets that train ML algorithms to identify correlations between atomic‐scale defect configurations and macroscopic performance outcomes.^[^ ^248^ ^]^ Recent advances in ML‐guided perovskite design have demonstrated the successful identification of optimal substituents that create shallow trap states and optimize band alignment, achieving significant photocatalytic enhancement.^[^ ^249^, ^250^ ^]^ Such integrated computational strategies enable high‐throughput screening of heterostructure combinations, establishment of quantitative structure‐activity relationships, and stability prediction under operational conditions, thereby providing atomic‐scale insights into defect‐tolerance mechanisms while offering practical guidance for rational heterostructure design optimization.

## Conflict of Interest

The authors declare no conflict of interest.
